# Multiple quantum ^19^F electron-nuclear double resonance (ENDOR) for distance measurements with multifluorinated spin probes

**DOI:** 10.1126/sciadv.aeg7753

**Published:** 2026-09-16

**Authors:** Sergei Kuzin, Lucca Sielaff, Karoline Goldschmidt, Marina Bennati

**Affiliations:** ^1^ Research Group EPR Spectroscopy, Max Planck Institute for Multidisciplinary Sciences, Am Faßberg 11, 37077 Göttingen, Germany.; ^2^ Institute of Physical Chemistry, Georg August University of Göttingen, Tammannstraße 6, 37077 Göttingen, Germany.

## Abstract

Mims electron–nuclear double resonance (ENDOR) spectroscopy with ^19^F spin labels is emerging as a powerful tool to access molecular distances in the 5 to 20 Å range. A time domain (TD) version of the experiment allows to manipulate nuclear spin coherences, similarly to NMR, and thus to increase distance resolution. Nevertheless, Mims ENDOR sensitivity strongly decays with the inter spin distance *r*, and measurements of longer distances benefit from labels with multiple ^19^F nuclei, such as CF_3_ probes. Here, we demonstrate that TD Mims ENDOR with two or three ^19^F nuclei reveals multiple-quantum effects and homonuclear dipolar couplings. We present a general theoretical framework for TD Mims ENDOR with multiple like nuclei (i.e., ^19^F), enabling analysis of spin evolution and the controlled experimental generation of single- and multiple-quantum nuclear coherences. Our results directly report not only on electron-nuclear distances but also on measurements and correlation of internuclear distances. Overall, TD ENDOR potentially offers a toolbox for molecular structure investigations.

## INTRODUCTION

Determination of inter-spin distances by electron paramagnetic resonance (EPR) and nuclear magnetic resonance (NMR) spectroscopies is essential for elucidating molecular structure and dynamics at the atomic and nanometer scale. Pulsed electron-nuclear double resonance (ENDOR) has been historically established as a powerful technique for probing the local structural environment of paramagnetic centers (*1*–*3*). Here, hyperfine interactions in the MHz range are usually detected, which enabled the characterization of transition metal complexes, radicals or biological macromolecules (*4*–*13*). In principle, ENDOR might be viewed as EPR-detected NMR spectroscopy; however, analogy to NMR spectroscopy in experimental design is complicated by the much larger electron magnetic moment as compared to nuclear spins (|γ_e_/γ_H_| ≈ 660), which leads to much larger magnetic interactions, including higher resonance frequencies and faster relaxation times, and requires different and more demanding instrumentation in the 10 to 300 GHz range.

For this reason, contrary to NMR, most ENDOR experiments are still performed in the so-called frequency domain (FD), probing hyperfine couplings by scanning radio frequency (RF) excitation frequencies. A few years ago, ^19^F Mims ENDOR has emerged as a powerful complementary tool in structural biology, providing access to distances in the range of 5 to 20 Å (*14*–*23*). It has been implemented with several paramagnetic labels, like nitroxides, trityls, Gd^3+^, and Cu^2+^, with applications to enzymes (*24*), nucleic acids (*17*, *18*, *20*, *21*), proteins (*16*, *19*, *23*), and in cells (*25*). Recently, we demonstrated the potential of time domain (TD) ENDOR experiments (*26*–*28*) in the context of ^19^F distance measurements. First, detection of the ENDOR spectrum via Fourier transform of the nuclear free-induction decay (FID) eliminates power broadening, which is one major source of broadening in ^19^F-FD ENDOR spectra (*29*). Second, the ability to refocus, and thereby separate, the ^19^F heteronuclear dipolar couplings to protons in vicinity via a nuclear spin echo, combined with the introduction of a similar coherence transfer scheme as in pulsed electron-electron double resonance/double electron-electron resonance (DEER) (*30*), allowed us to acquire electron-nuclear dipolar traces with substantial resolution enhancement, down to a few kilohertz (*31*).

While TD ENDOR improves resolution, recent reports by other authors pointed out the intrinsic sensitivity issue in Mims ENDOR (*19*, *22*), which currently limits the accessible distances to about *r* ≲ 2 nm in a biological matrix. The Mims ENDOR signal at small hyperfine frequencies (<<1 MHz) is attenuated by a blind spot that scales with ∼1/*r*^6^ (*27*). Thus, longer distances become increasingly difficult to detect due to the reduction of the ENDOR efficiency. While most of ^19^F Mims ENDOR experiments are performed using mono-fluorinated labels, it has been shown that sensitivity benefits from spin labels with multiple ^19^F nuclei, such as the well-known CF_3_ or SF_5_ probes, because of their combined contribution to the ENDOR signal (*22*). Therefore, it would be important to combine the increased sensitivity of multiple-^19^F spin probes with the increased resolution of TD ENDOR to access longer distances.

Multispin probes introduce a layer of complexity in TD ENDOR experiments, as homonuclear couplings are not refocused in a nuclear echo, and multiple-spin orders are created. Previous seminal work in pulse ENDOR spectroscopy established that multiple-spin orders of an electron spin coupled to several, almost equivalent protons, can be used to perform so-called multiple quantum (MQ) ENDOR spectroscopy (*32*, *33*). Excitation of MQ coherences was demonstrated to map the delocalization of the soliton wave function in the polyacetylene radical (*34*, *35*), or to count the number of equivalent protons in water molecules coordinated in a Cu^2+^ hexaaqua complex (*36*). The principle of these experiments relies on the time evolution properties of multiple-spin orders, which depend either on the nutation angle of the ENDOR RF pulse (*36*) or on a phase incrementation experiment in TD ENDOR (*34*). The phase incrementation approach separates MQ coherences using their rotational symmetry about the *z* axis (*34*, *37*). This idea is closely related to the well-known technique of MQ NMR (*37*–*39*).

MQ EPR spectroscopy with two or more electron spins has been established for distance measurements between paramagnetic centers (*40*–*42*). However, MQ ENDOR spectroscopy, proposed more than 30 years ago, has not been broadly implemented yet. Practical realization has been hindered by hardware requirements, including the need for high-power coherent RF irradiation circuits. ENDOR setups are generally nonresonant, resulting in a reduced available RF power and thus a limited pulse band width [≲200 kHz, at Q band (*31*)]. Moreover, sufficiently long electron relaxation times as well as more involved theoretical modeling including nuclear-nuclear interactions are required to understand the spin dynamics. The emerging scientific case of ^19^F Mims ENDOR distance measurements using multiple ^19^F probes, facilitated by the small detected hyperfine couplings, provides an opportunity to revisit and expand previous work on MQ, multispin effects in ENDOR.

**Fig. 1.**
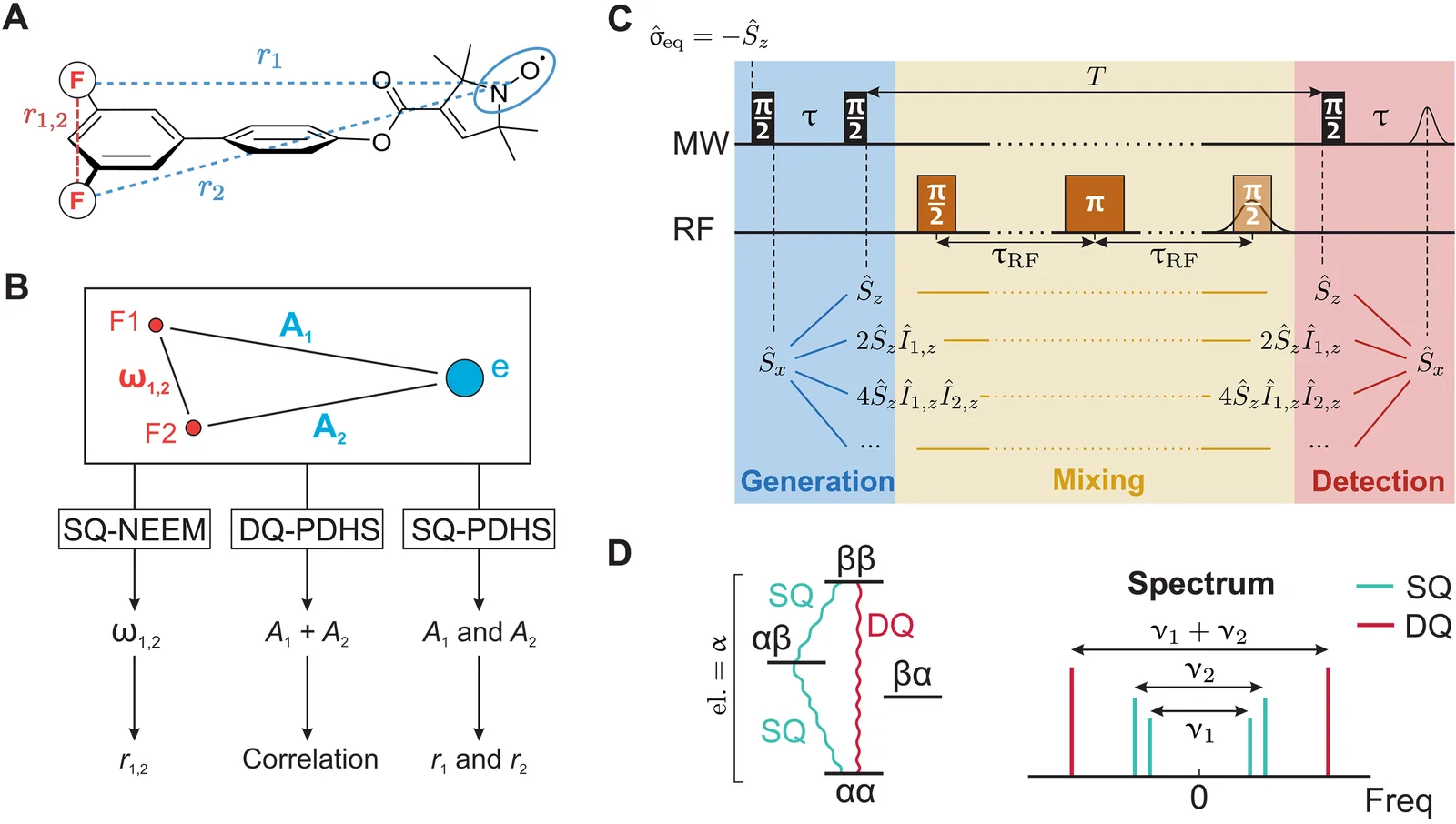
Principle of TD ENDOR in a multispin system*.* (**A**) Representative molecule studied in this work: A nitroxide radical carrying an electron spin (blue oval) is magnetically coupled to two fluorine nuclei, which are also mutually interacting. (**B**) The three- spin system from (A) is investigated by different experiments in this work. Single-quantum (SQ) pulse dipolar hyperfine spectroscopy (PDHS) reports two electron-^19^F distances, double-quantum (DQ) PDHS correlates the two hyperfine couplings *A*_1_ and *A*_2_, SQ NEEM measures the nuclear dipolar coupling $\omega_{1,2}$. (**C**) General pulse scheme of Mims TD ENDOR. Colors highlight generation, mixing and detection of (multi-)spin orders. The Mims generation block converts electron coherence into various spin order terms. They are manipulated during the mixing part, which includes changing their weights in density matrix and eventually interconversion (mixing). Ultimately, the detection part, which is symmetric to the generation, reconverts spin orders into electron coherence, followed by detection as an electron spin echo. The third RF pulse is transparent to show the nuclear spin echo. (**D**) Sublevels for two coupled nuclei in the electron α manifold. Turquoise lines indicate SQ nuclear transitions with frequencies $\nu_{1}$ and $\nu_{2}$, the red line corresponds to a DQ nuclear transition with frequency $\nu_{1}+\nu_{2}$.

Here, we extend TD Mims ENDOR distance measurements to multinuclear ^19^F spin probes. We first present a theoretical, analytical framework involving multiple coupled nuclear spins of the same kind (homonuclear case) to describe TD ENDOR, and then apply it to analyze two types of experiments, pulsed dipolar hyperfine spectroscopy (PDHS) (*31*) and nuclear echo envelope modulation (NEEM). To investigate the capabilities of these methods, we employ rationally designed model systems containing two and three fluorine atoms. The concept of the work is shown in Fig. 1 for the two-fluorine case (Fig. 1, A and B). The general pulse scheme of TD Mims ENDOR is illustrated in Fig. 1C. The EPR observer sequence is a stimulated echo (1), and all RF pulses are assumed strong and nonselective to excite the entire hyperfine spectrum. The basic experiment consists of creation, refocusing and read-out (or detection) of nuclear coherences, while its variants differ in additional RF and microwave (MW) pulses during the mixing block. For instance, in a system with two coupled nuclei (*N* = 2, Fig. 1C), the generation block creates electron-nuclear two-spin order terms $2{\hat{S}}_{z}{\hat{I}}_{1,z}$ and $2{\hat{S}}_{z}{\hat{I}}_{2,z}$ as well as a three-spin order term $4{\hat{S}}_{z}{\hat{I}}_{1,z}{\hat{I}}_{2,z}$, which are converted into nuclear coherence by the first RF π/2 pulse. Nuclear coherences evolve, and eventually also interconvert, until the last RF π/2 read-out pulse converts them back to nuclear polarization and spin-orders, which can be observed in the electron stimulated echo (Fig. 1C).

In the simplest implementation of TD ENDOR, only two RF pulses are employed and the delay between them is incremented, to read-out the nuclear FID (*26*–*29*). By inserting one more RF π pulse, a nuclear spin echo is formed, allowing for refocusing interactions that are linear in the nuclear spin operators, such as chemical shift, hyperfine coupling, and heteronuclear dipolar coupling. The intensity of the nuclear echo depends on the inter pulse delay τ_RF_, permitting measurements of nuclear *T*_2_ (*26*, *29*, *43*). Here, we show that for systems containing several like nuclear spins (*N* > 1), the intensity of the nuclear echo as a function of τ_RF_ is additionally modulated by homonuclear couplings, giving rise to an experiment we call NEEM. Moreover, additional MQ coherences are generated (*33*), which lead to combination frequencies (Fig. 1, B and D). Our results for *N* = 2 and 3 nuclei reveal detection of pure double and triple quantum nuclear coherences as well as homonuclear ^19^F dipolar couplings in PDHS and NEEM experiments, respectively (Fig. 1B). This additional spectral information enabled extraction of internuclear spin distances and their correlations to electron-nuclear distances. This work paves the way for introducing multiple-spin probes in high-resolution, high-sensitivity ^19^F ENDOR distance measurements.

## RESULTS AND DISCUSSION

### Spin Hamiltonian

We present a theoretical model to explain the observed TD ENDOR signal for a system of an electron spin coupled to *N* nuclei of spin 1/2 (e.g., ^1^H, ^19^F, ^31^P, etc.) as exemplified in Fig. 1A. We assume that the nuclei are of the same kind (homonuclear case) and can be excited simultaneously by strong RF pulses. The spin Hamiltonian includes the following four interactions

$$\begin{array}{c} \hat{H}={\hat{H}}_{\text{EZ}}+{\hat{H}}_{\text{HF}}+{\hat{H}}_{\text{CS}}+{\hat{H}}_{\text{ND}}=\Omega {\hat{S}}_{z}\\ +\sum_{j=1}^{N}A_{j}{\hat{S}}_{z}{\hat{I}}_{j,z}+\sum_{j=1}^{N}{\text{Δω}}_{j}{\hat{I}}_{j,z}+\sum_{j<k}\omega_{j,k}{\hat{I}}_{j,z}{\hat{I}}_{k,z} \end{array}$$ (1)

where EZ stands for electron Zeeman, HF for hyperfine, CS for chemical shift, and ND for nuclear dipolar interaction. This Hamiltonian is written in a rotating frame associated with both electron spin and nuclear spins. $\Omega =\omega_{e}-\omega_{\text{MW}}$ and ${\text{Δω}}_{j}=\omega_{\text{Larmor},j}-\omega_{\text{RF}}$ are the electron and nuclear resonance offsets, respectively. $A_{j}$ are the hyperfine coupling constants, and $\omega_{j,k}$ are nuclear-nuclear coupling constants. Given the measurement conditions used in this work (static solids, cryogenic temperatures, and electron-^19^F distances longer than 1 nm) we neglect the Fermi-contact and the *J*-coupling and assume a dipolar model for both hyperfine and nuclear interactions. We apply the high-field approximation to nuclear spins, which sets the external magnetic field (*z* direction) as the spin quantization axis. Consequently, the homonuclear interaction appears in truncated form, which is feasible if the difference in hyperfine couplings exceeds the coupling constant, i.e., $|\omega_{j,k}|\ll |A_{j}-A_{k}|$. We explicitly note that the nuclear resonance offsets can be different due to a possible distribution of nuclear chemical shifts (*44*). Besides, as argued in section S1.1, nuclear offsets can include the effects of heteronuclei, e.g., molecular protons and solvent deuterons.

### Generation block

The first MW π/2 pulse in the Mims ENDOR experiment (Fig. 1C) converts the equilibrium electron spin polarization into coherence described by a density matrix $\hat{\sigma }={\hat{S}}_{x}$. After excitation, the coherence evolves under the hyperfine coupling with *N* nuclei (*I* = ½) simultaneously. We provide a comprehensive analytical description of this evolution step in section S1, facilitated by conducting the analysis in the spherical basis (*45*). By applying the second π/2 pulse, the electron magnetization is turned to *z* direction. However, because of the interaction with nuclei, the density matrix contains ${\hat{S}}_{z}$ and electron-nuclear longitudinal spin order operators. For an electron spin and two nuclei (*N* = 2) we find

$$\begin{array}{c} \hat{\sigma }\left(\tau \right)=\text{cos}\text{Ωτ cos}\frac{A_{1}\tau }{2}\text{cos}\frac{A_{2}\tau }{2}{\hat{S}}_{z}-\text{sin}\text{Ωτ sin}\frac{A_{1}\tau }{2}\text{cos}\frac{A_{2}\tau }{2}2{\hat{S}}_{z}{\hat{I}}_{z,1}\\ -\text{sin}\text{Ωτ cos}\frac{A_{1}\tau }{2}\text{sin}\frac{A_{2}\tau }{2}2{\hat{S}}_{z}{\hat{I}}_{z,2}-\text{cos}\text{Ωτ sin}\frac{A_{1}\tau }{2}\text{sin}\frac{A_{2}\tau }{2}4{\hat{S}}_{z}{\hat{I}}_{z,1}{\hat{I}}_{z,2} \end{array}$$ (2)

Here, the presence of the electron spin frequency offset ( Ω ) is important as it allows to generate both one-nucleus terms ${\hat{S}}_{z}{\hat{I}}_{1,z}$, ${\hat{S}}_{z}{\hat{I}}_{2,z}$, and the two-nuclei term ${\hat{S}}_{z}{\hat{I}}_{z,1}{\hat{I}}_{z,2}$ with nonzero amplitudes. Despite of the explicit τ dependence, a typically broad distribution of Ω causes that each term in Eq. 2 contributes with equal weight 1/2 to the final ENDOR signal. This is discussed more detailed in section S1.6. As a result, factors cosΩτ and sinΩτ can be formally omitted in further analysis. This said, generalization of Eq. 2 for *N* nuclei shows that the density matrix after preparation block is a sum of the following product terms

$$c_{g}\left(X,\tau \right)2^{|X|}{\hat{S}}_{z}{\hat{I}}_{X,z}\left(X\subseteq \bar{1,N}\right)$$ (3)

**Fig. 2.**
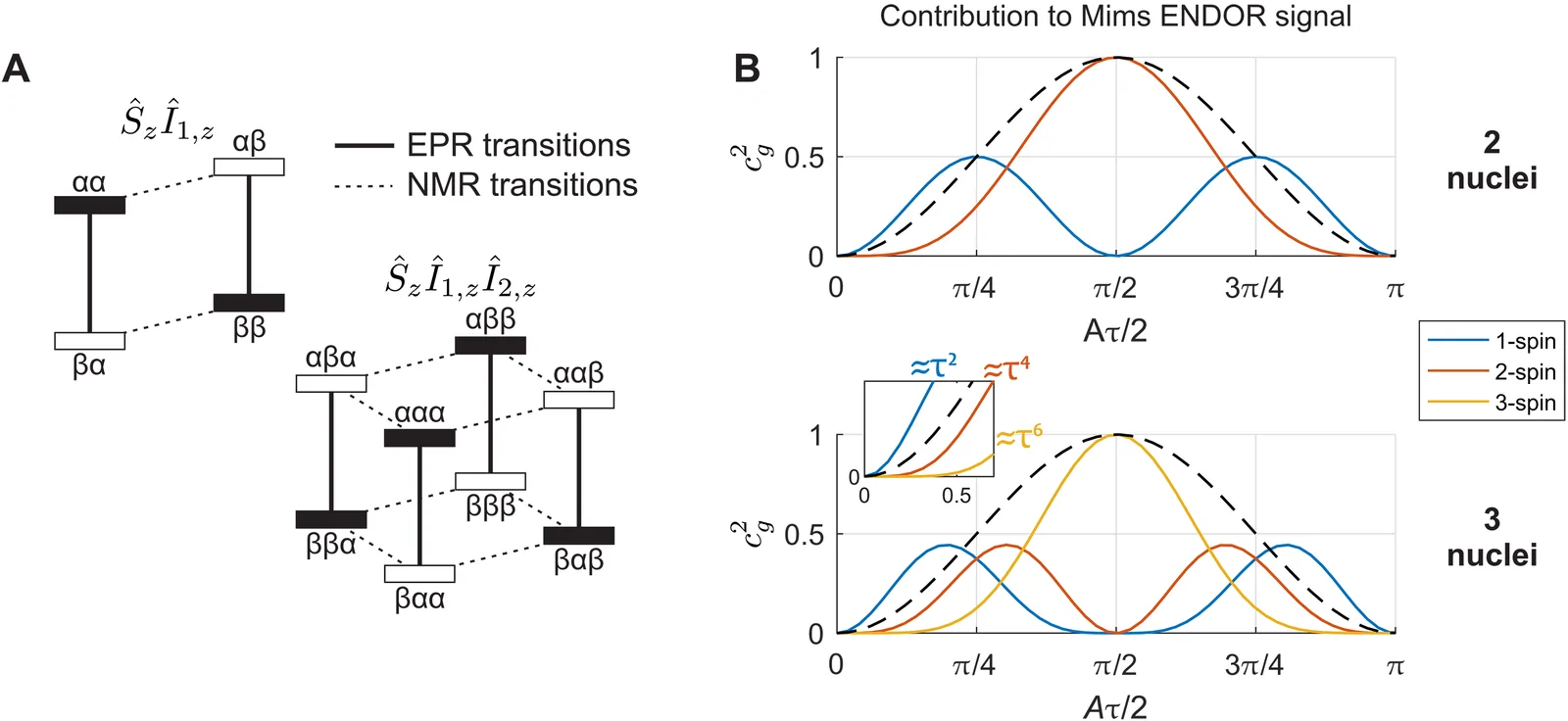
Nuclear orders in Mims ENDOR. (**A**) Spin orders in the density matrix describe the nonequilibrium population of spin levels. The first symbol is reserved for the electron spin. Black and white boxes represent the higher and the lower population of levels, respectively. (**B**) Weight factors $c_{g}{\left(X;\tau \right)}^{2}$ of 1-, 2- and 3-nuclear orders after the generation block in a two- (top, *N* = 2) and three-spin system (bottom, *N* = 3). The squared form of $c_{g}$ was chosen according to their contribution to ENDOR signals (see Eq. 15). The dashed line is the ENDOR efficiency in standard single-nucleus ENDOR, ${\text{sin}}^{2}\frac{A\tau }{2}$. The inset demonstrates the suppression of the high-order terms at short preparation periods, compared to the ${\hat{S}}_{z}{\hat{I}}_{z}$ term (blue line). Here, for simplicity, hyperfine coupling constants of all nuclei are set equal to the same constant *A*.

In this expression, $\bar{1,N}$ is a set of integers from 1 to *N*, *X* is a subset of $\bar{1,N}$, ∣X∣ is the size of *X*, $2^{|X|}$ is the normalization constant and ${\hat{I}}_{X,z}$ denotes a product of spin *z* operators indexed by subset X (by definition, this product equals the identity operator if X is empty). We note that the longitudinal order number here is ∣X∣+1. Longitudinal spin orders describe nonequilibrium (non-Boltzmann) populations of the spin states, as shown in Fig. 2A. Under conditions used in this work, nuclear polarization can be neglected; therefore, in thermal equilibrium, all states within the same electron spin manifold are populated equally. The Mims preparation block thus creates a nonequilibrium initial condition for the NMR part of the ENDOR pulse sequence.

$c_{g}\left(X,\tau \right)$ in Eq. 3 is a τ-dependent factor with which a generated multispin order $2^{|X|}{\hat{S}}_{z}{\hat{I}}_{X,z}$ is weighted. These coefficients are expressed by a general formula

$$c_{g}\left(X,\tau \right)={\left(-1\right)}^{\left\lceil \frac{|X|}{2}\right\rceil }\left(\prod_{j\in X}\text{sin}\frac{A_{j}\tau }{2}\right)\left(\prod_{k\notin X}\text{cos}\frac{A_{k}\tau }{2}\right)$$ (4)

Note that for *N* = 1, $c_{g}\left(\{1\},\tau \right)=-\text{sin}\frac{A\tau }{2}$, which leads to the classical blind spot function in ENDOR spectroscopy. For *N* = 2, the weights of the product operator term $2{\hat{S}}_{z}{\hat{I}}_{1,z}$ ( X={1} and ∣X∣=1 ) is

$$c_{g}\left(\{1\},\tau \right)=-\text{sin}\frac{A_{1}\tau }{2}\text{cos}\frac{A_{2}\tau }{2}$$ (5)

where the index 1 brings a sine factor and every other index (here: 2) enters with a cosine-factor. τ modulation of the multispin terms in several cases is exemplified in Fig. 2B. Equation 4 predicts that the τ modulation of the simplest spin orders (e.g., ${\hat{S}}_{z}{\hat{I}}_{1,z}$ ) involves a sine factor, as anticipated from the well-described case *N* = 1 (3), and cosine factors from all other nuclei as well. As a result, the generation of single-nuclear terms is now correlated with other nuclei, and the τ dependence deviates from the pure sin-pattern (black dashed lines in Fig. 2B). We refer to this phenomenon as the multispin effect in ENDOR spectroscopy.

### Additive approximation at short τ

*n*-Nuclear order terms contain *n* sine factors; hence, they are suppressed at short τ, namely, as $\approx \tau^{n}$ (see Fig. 2B). Accordingly, at sufficiently short Mims preparation blocks, such that $A_{j}\tau \ll \pi$, we can neglect products of sine functions and approximate $\text{cos}\left(\frac{A_{j}\tau }{2}\right)\approx 1$. Therefore, the density matrix after the preparation block is simply

$$\hat{\sigma }\approx {\hat{S}}_{z}\left(\text{cos}\text{Ωτ}\hat{1}-\text{sin}\text{Ωτ}\sum_{j}\text{sin}\frac{A_{j}\tau }{2}2{\hat{I}}_{j,z}\right)$$ (6)

In this regime, the generation of the multispin orders is suppressed [$c_{g}\left(X\right)\approx 0$ if ∣X∣>1], which is illustrated in Fig. 2B. The generated single-nuclear terms are uncorrelated, i.e., the multispin effect also disappears. The ENDOR signal then simplifies to

$$V=\sum_{j=1}^{N}V_{j}$$ (7)

where $V_{j}$ is the signal assuming only one coupled nucleus. We refer to this regime as additive approximation.

### Mixing block and principles of MQ ENDOR spectroscopy

The mixing block starts with a broadband coherent RF π/2 pulse that nutates the nuclear spins. In the multinuclear spin orders, nutation of each spin operator happens independently, e.g., in the two-nuclear spin case

$$\begin{array}{c} {\hat{S}}_{z}{\hat{I}}_{1,z}{\hat{I}}_{2,z}\overset{\frac{\pi }{2}\left({\hat{I}}_{1,\phi }+{\hat{I}}_{2,\phi }\right)}{⟶}{\hat{S}}_{z}\left({\hat{I}}_{1,x}\text{sin}\phi -{\hat{I}}_{1,y}\text{cos}\phi \right)\times \\ \left({\hat{I}}_{2,x}\text{sin}\phi -{\hat{I}}_{2,y}\text{cos}\phi \right) \end{array}$$ (8)

Here, ϕ is the phase of the RF pulse (0 for “+x,” π/2 for “+y,” etc.). After multiplication and application of trigonometric identities, the density operator exhibits double-quantum coherences that respond to the phase of the excitation pulse as cos2ϕ and sin2ϕ. In the following, we are going to show how evolution of these terms is generally computed.

As it was done for the derivation of Eq. 3, further analytical calculations again benefit from representing the density matrix in the spherical basis. An excitation of a general spin order with an RF pulse of phase ϕ reads

$$2^{|X|}{\hat{S}}_{z}{\hat{I}}_{X,z}\overset{\frac{\pi }{2}{\hat{F}}_{\phi }}{⟶}\sum_{s\left(X\right)}e^{-\mathrm{ip}\left(\phi -90^{\circ }\right)}{\hat{S}}_{z}{\hat{I}}_{X}^{s}$$ (9)

Here, operator ${\hat{F}}_{\phi }$ means a sum of nuclear operators with the same phase ϕ, and ${\hat{I}}_{X}^{s}$ is a shorthand notation for a product of raising and lowering operators with indices from the subset *X*. **s** is an *N*-dimensional array whose elements *s_i_* attain ±1 or 0. *s_i_* = 0, if nucleus *i* is absent in *X*; +1 and −1 correspond to ${\hat{I}}^{+}$ and ${\hat{I}}^{-}$, respectively. The sum of all *s_i_*, denoted by *p*, is the total nuclear coherence order. For example, if *N* = 2 and **s** = (+1, +1), then ${\hat{I}}_{\left\{1,2\right\}}^{s}$ is short for ${\hat{I}}_{1}^{+}{\hat{I}}_{2}^{+}$ which represents a two-spin nuclear coherence of order *p* = +2. In addition, ${\hat{I}}_{\left\{\right\}}^{s}$ (*X* is an empty subset) is defined as a unity operator $\hat{1}$. Summation in Eq. 9 is done over all arrays **s** that can be generated for a given nuclear subset *X*.

It is convenient to arrange the interaction constants as vectors $A=\left(A_{1},\ldots ,A_{N}\right)$, $∆\omega =\left(∆\omega_{1},\ldots ,∆\omega_{N}\right)$ and $\omega_{k}=\left(\omega_{k,1},\ldots ,\omega_{k,N}\right)$. With this notation, the free evolution of a coherence ${\hat{S}}_{z}{\hat{I}}_{X}^{s}$ under Hamiltonian (1) for time *t* is written as (section S1.7)

$${\hat{S}}_{z}{\hat{I}}_{X}^{s}\overset{\hat{H}t}{⟶}e^{-i∆\omega \cdot st}e^{-iA\cdot st{\hat{S}}_{z}}\left(\prod_{k\notin X}e^{-i\omega_{k}\cdot st{\hat{I}}_{k,z}}\right){\hat{S}}_{z}{\hat{I}}_{X}^{s}$$ (10)

The three operator exponentials arise from the evolution under chemical shift, hyperfine coupling and nuclear coupling, respectively. In this formula, the product of zero factors is 1 by definition (if $X=\bar{1,N}$ ). The scalar products are simply linear combinations of coupling constants, e.g., $s\cdot A\equiv \sum_{j\in X}s_{j}A_{j}$. This formula predicts that the hyperfine coupling affects the spins in the subset *X*. The nuclear-nuclear coupling does not affect the coherence propagation if both coupled nuclei are simultaneously involved or not involved in a given coherence. Further, application of MW π pulses leads to transformation ${\hat{S}}_{z}\to -{\hat{S}}_{z}$. Application of nonselective RF π pulses is equivalent to inversion of all ${\hat{I}}_{z}\to -{\hat{I}}_{z}$ and the sign flip of vector **s**. Combining these rules with the free evolution in Eq. 10 allows to design ENDOR experiments to read out either the hyperfine interaction or the nuclear-nuclear interaction. Furthermore, varying the phase of RF pulses enables the manipulation of coherence phases. This applies to RF π/2 pulses for coherence generation (Eqs. 8 and 9) as well as to π pulses for state inversion

$${\hat{S}}_{z}{\hat{I}}_{X}^{s}\overset{\pi {\hat{F}}_{\phi }}{⟶}e^{i2p\phi }{\hat{S}}_{z}{\hat{I}}_{X}^{-s}$$ (11)

By the time of the last RF π/2 pulse application (Fig. 1C), the coherence term under consideration obtains a phase factor exp(−ipΦ), where Φ is a sequence-specific linear combination of RF pulses’ phases. This phase dependence is at the basis of the time proportional phase increment experiment and phase cycling protocols to separate different MQ orders (*38*, *40*, *41*, *46*–*48*). We refer to the latter as *p*-quantum filters. The quadrature detection of the nuclear echo can be achieved by performing the phase cycling with Φ and Φ + 90° (*27*, *46*). Here, we emphasize the difference between the multispin and multiple-quantum effects in our treatment. Excitation of multispin orders generated in the Mims preparation block leads to a mixture of coherences of various orders. For example, excitation of the term $4{\hat{S}}_{z}{\hat{I}}_{z,1}{\hat{I}}_{z,2}$ results in $4{\hat{S}}_{z}{\hat{I}}_{x,1}{\hat{I}}_{x,2}$, which can be rewritten as ${\hat{S}}_{z}\left({\hat{I}}_{1}^{+}{\hat{I}}_{2}^{+}+{\hat{I}}_{1}^{-}{\hat{I}}_{2}^{-}\right)+{\hat{S}}_{z}\left({\hat{I}}_{1}^{+}{\hat{I}}_{2}^{-}+{\hat{I}}_{1}^{-}{\hat{I}}_{2}^{+}\right)$, i.e., a sum of double-quantum and zero-quantum coherences. Also, for this work, the three-nuclear product term $4{\hat{S}}_{z}{\hat{I}}_{x,1}{\hat{I}}_{x,2}{\hat{I}}_{x,3}$ is a mixture of coherences of order three ${\hat{S}}_{z}\left({\hat{I}}_{1}^{+}{\hat{I}}_{2}^{+}{\hat{I}}_{3}^{+}+{\hat{I}}_{1}^{-}{\hat{I}}_{2}^{-}{\hat{I}}_{3}^{-}\right)$ and one ${\hat{S}}_{z}\left({\hat{I}}_{1}^{+}{\hat{I}}_{2}^{+}{\hat{I}}_{3}^{-}+\ldots \right)$. Hence, the coherences of order ±1 in *N* = 3 are represented by both one-spin and three-spin terms.

### Detection

Spin manipulation during the mixing part shapes a new multispin state that can be read out via the intensity of the electron spin echo (detection part in Fig. 1C). Since the detection part is symmetric with the generation part, when disregarding the first MW excitation pulse, only those elements of the nuclear density matrix can be detected that have been generated at the beginning. In our case, these are the longitudinal spin orders as in Eq. 3. On the basis of this, we represent the signal generation multispin Mims ENDOR by independent paths

$${\hat{S}}_{x}\to 2^{|X|}{\hat{S}}_{z}{\hat{I}}_{X,z}\to 2^{|Y|}{\hat{S}}_{z}{\hat{I}}_{Y,z}\to {\hat{S}}_{x}$$ (12)

Such paths correspond to different lines connecting the starting and final ${\hat{S}}_{x}$ operators in Fig. 1C. The signal contribution from such a path (where spin order *X* is generated and spin order *Y* is detected) is a product of three factors

$$V_{X,Y}=c_{g}\left(X\right)m\left(X\to Y\right)c_{d}\left(Y\right)$$ (13)

where m(X→Y) is the mixing amplitude from spin order *X* to spin order *Y* and it depends on the manipulation history in the mixing block. $c_{d}\left(Y\right)$ is the detection amplitude of the spin order *Y*, i.e., the coefficient with which spin order *Y* is converted to the observable electron coherence during the detection block. Thanks to the mirror symmetry of the detection with respect to generation, we find $c_{d}\left(Y,\tau \right)=c_{g}\left(Y,\tau \right)$. The total signal is the sum over all possible paths

$$V=\sum_{X,Y}V_{X,Y}$$ (14)

This is a general expression describing an arbitrary TD ENDOR experiment with *N* manipulated nuclei. In NEEM and PDHS, nuclear coherences are affected by an RF π pulse that does not change the absolute value of the coherence order, although it flips its sign. As a result, nuclear spin orders are not interconverted, i.e., only m(X→X) are nonzero, and Eq. 14 can be simplified

$$V_{\text{NEEM}/\text{PDHS}}=\sum_{X}V_{X,X}=\sum_{X}c_{g}{\left(X\right)}^{2}m\left(X\to X\right)$$ (15)

Because of this specific form, we plotted squares of $c_{g}$-coefficients in Fig. 2B.

### Model compounds

**Fig. 3.**
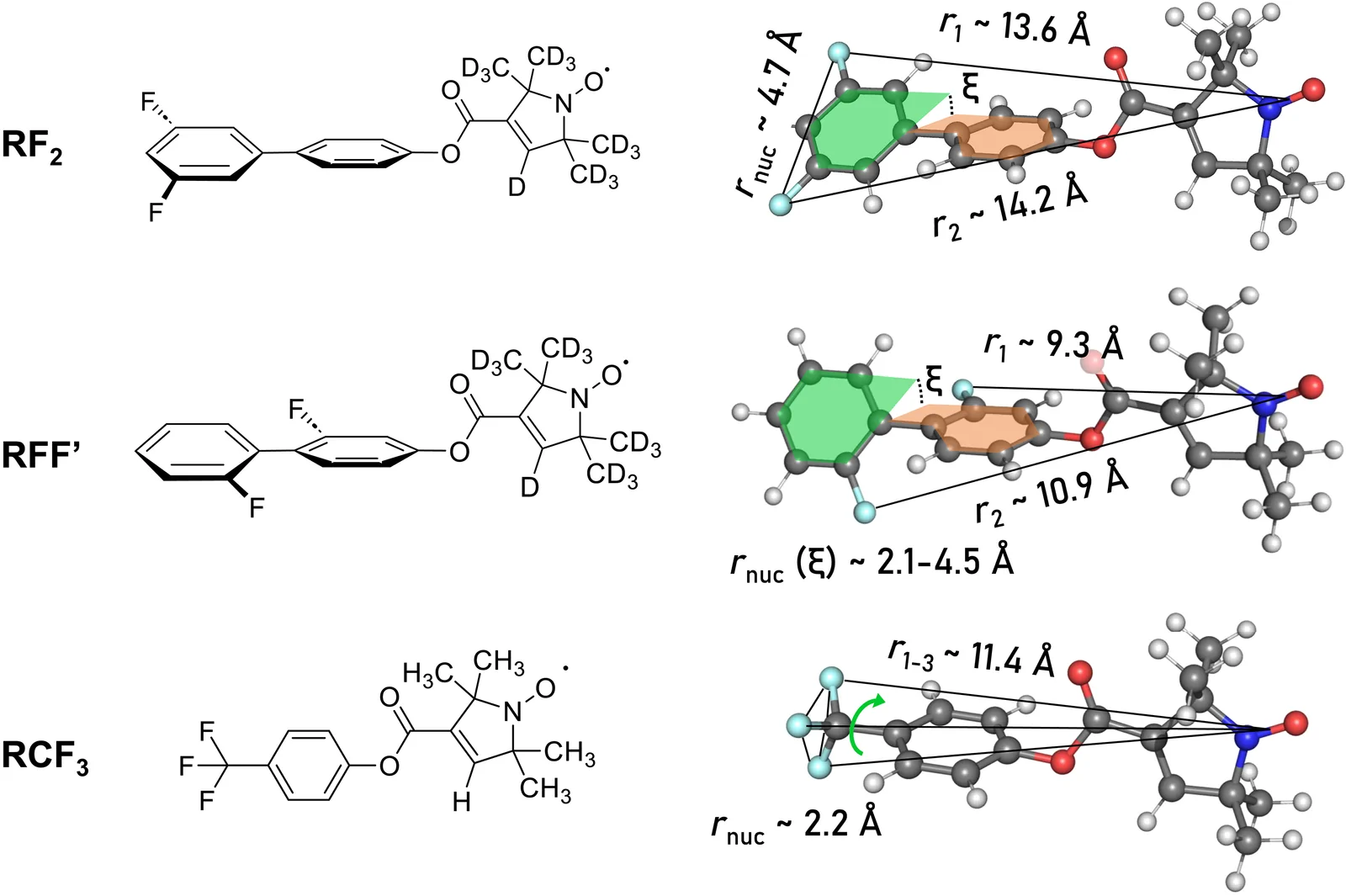
Model compounds. Left: Chemical structures of compounds **RF**_**2**_, **RFF′**, and **RCF**_**3**_. Right: Density functional theory (DFT)–optimized geometries of the model compounds. Relevant inter spin distances are indicated as solid black lines. Labels *r*_1–3_ mark nitroxide-^19^F distances and *r*_nuc_ mark ^19^F-^19^F distances. The angle ξ is the dihedral between the two aromatic rings in model compounds **RF**_**2**_ and **RFF′**. A green arrow indicates the rotation of the CF_3_ group in model compound **RCF**_**3**_.

To study multispin effects in TD ENDOR, we synthesized three nitroxide model compounds containing either two (compounds **RF**_**2**_, **RFF′**) or three fluorine atoms (compound **RCF**_**3**_). The structures of the compounds are shown in Fig. 3. Nitroxide-^19^F hyperfine dipolar couplings and corresponding point-dipole distances were estimated from the density functional theory (DFT)–optimized geometries. For the nitroxide radical, we considered the spin center as localized in the mid of the N─O bond (*14*). Details on the synthesis and DFT calculations are provided in sections S2 and S3. The model compounds **RF**_**2**_ and **RCF**_**3**_ were designed to exhibit minimal flexibility along the nitroxide-^19^F distance vector (*14*), leading to a narrow distance distribution and thereby sharp ENDOR spectra. This approach enables us to obtain data illustrating the effects of multispin interactions, which we also validate through numerical simulations. To examine the case of a more complex internuclear distance distribution, we synthesized the model compound **RFF′**. We highlight in Fig. 3 the degrees of freedom that influence the interspin distances and relative orientation of the hyperfine and nuclear-nuclear coupling tensors. In the model compounds **RF**_**2**_ and **RFF′**, this is the rotation around the single C─C bond of the biphenyl residue, characterized by the dihedral angle ξ. In model compound **RCF**_**3**_, this is the uniaxial rotation of the CF_3_ group. We note that rotational flexibility along the C─O bond in the ester group is expected to be negligible thanks to its partial double bond character in the conjugated O─C═O system. Thus, as in previous studies, we assume a rigid ester group (*14*).

### TD ENDOR with RF_2_ (*N* = 2): Observation of NEEM

**Fig. 4.**
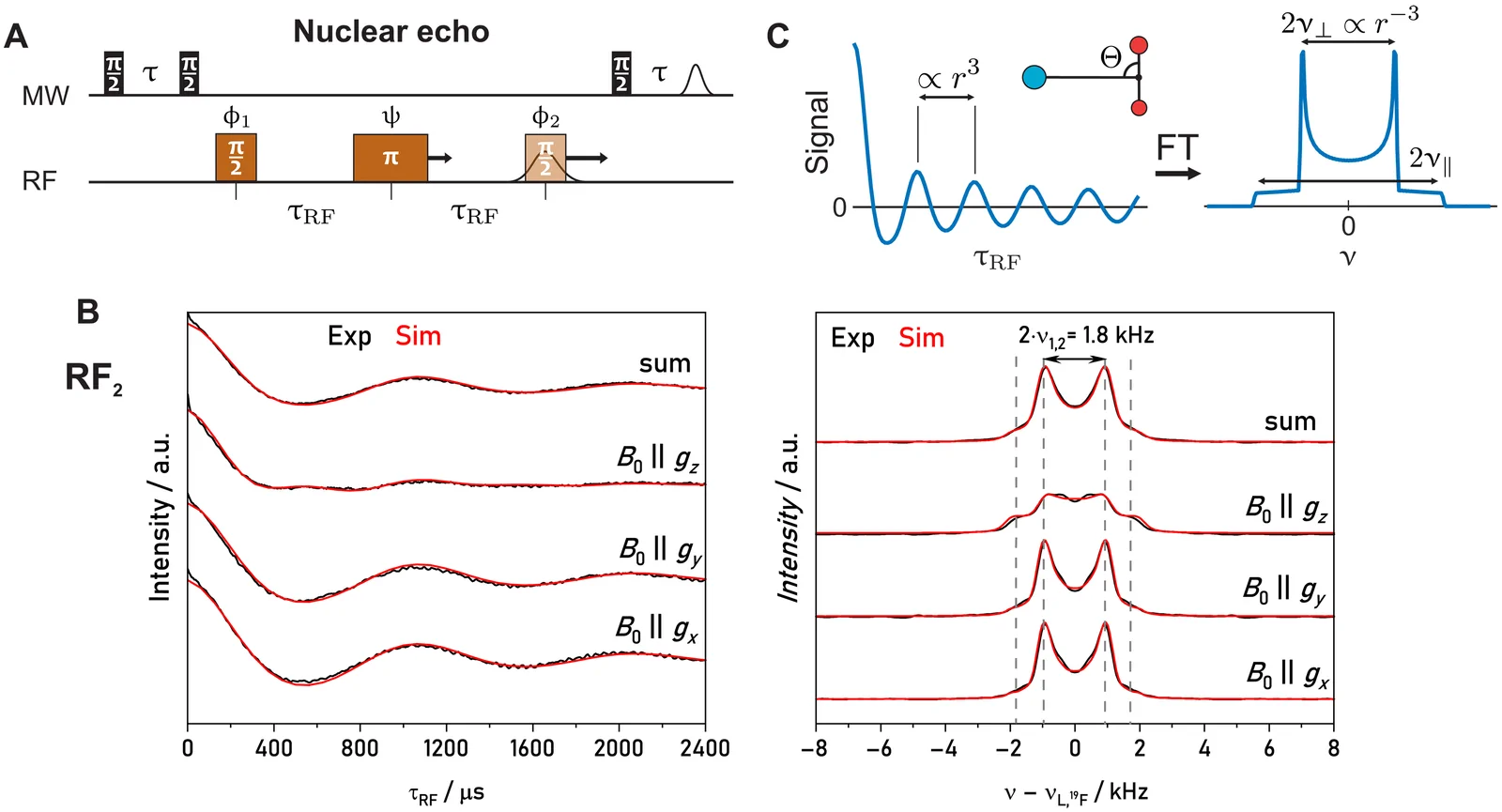
NEEM experiments of RF_2_. (**A**) NEEM pulse sequence. The nuclear echo intensity is recorded as a function of the inter pulse delay τ_RF_, which is incremented. The central delay on the MW channel is kept constant to exclude signal decay due to the electron relaxation and spectral diffusion (*57*). The phases of the RF pulses (orange boxes) are generally denoted as $\phi_{1}$, ψ, and $\phi_{2}$. (**B**) Left: NEEM traces of compound **RF**_**2**_ recorded at 20 K at Q band with 12 ns MW π/2 pulses, 4 μs RF π/2 pulses, τ = 3 μs, 70 ms shot-repetition time (srt), and 2 shots per point (spp) at three different positions of the EPR spectrum corresponding to the principal orientations of the *g* tensor. The second RF pulse was stepped in 6 μs steps. RF phase cycling for this experiment is shown in “Materials and Methods.” Simulated NEEM traces using analytical expressions derived in this work (see Eq. 18, more details in “Data simulation” and section S5) are shown as solid red lines. Right: Fourier transform of the experimental (black) and simulated (red) NEEM time traces. (**C**) Schematic NEEM data for a two-nuclei system. Powder-averaged time trace (left) and its real Fourier transform (right). The spectrum is fundamentally close to the Pake pattern. Its deviation from the Pake pattern depends on τ and on the relative orientation of hyperfine and nuclear dipolar tensors (Θ). The shown example corresponds to Θ ≈ 90° (see section S5.2). Characteristic features are marked in the time and FDs. *r* is the internuclear distance, $\nu_{⊥}=\frac{1}{2}\nu_{∥}=\mu_{0}/\left(4\pi h\right)\cdot g_{n}^{2}\mu_{N}^{2}/r^{3}$ is a principal value of the nuclear dipolar coupling tensor.

TD ENDOR techniques presented in this work, performed at 34 GHz/1.2 Tesla, rely on the detection of nuclear spin echoes (Figs. 1C and 4A). In the course of initial experiments with **RF**_**2**_, we unexpectedly found a vanishingly small ^19^F-echo intensity at some specific values of τ_RF_ despite the intense EPR signal. To explore this, we recorded the nuclear echo intensity as a function of delay τ_RF_ and observed strong signal modulation as displayed in Fig. 4B. The corresponding Fourier transform spectrum resembled a Pake pattern with a splitting of ∼2 kHz. We realized that this is consistent with the dipole-dipole interaction expected between the two ^19^F nuclei $\nu_{⊥}=\frac{\mu_{0}}{4\pi h}\cdot \frac{g_{n}^{2}\mu_{N}^{2}}{r^{3}}=106\text{kHz}\cdot \frac{{Å}^{3}}{r^{3}}$, where r=4.7Å is from the DFT-optimized geometry (Fig. 3). This modulation effect and the corresponding experiment will be referred here to as NEEM, akin to its well-known electron spin analogue, ESEEM (*49*–*51*).

Our observation can be rationalized by considering that the ^19^F π pulse refocuses all interactions in the spin Hamiltonian that are linear with respect to fluorine, such as chemical shift, hyperfine coupling, and coupling with protons (*26*). Hence, for singly fluorinated compounds, only relaxation is expected to affect the nuclear echo (*29*, *43*). In **RF**_**2**_ and other multi-fluorinated compounds, the evolution under homonuclear interaction is not refocused by the nonselective RF π pulse (*52*). As a result, the nuclear echo is modulated by

$$V\left(\tau_{\text{RF}}\right)\propto \text{cos}\left(\omega_{1,2}\tau_{\text{RF}}\right)$$ (16)

where $\omega_{1,2}$ is the ^19^F-^19^F coupling constant. Since dipolar interaction is anisotropic, $\omega_{1,2}$ is distributed, and the net echo reduction is due to destructive interference. The nuclear dipolar spectra measured at different positions within the EPR spectrum exhibit different shapes, indicating a correlation between the fluorine-fluorine vector and the nitroxide’s *g* tensor. This correlation is attributed to the rigidity of the benzene unit that bears fluorine atoms. However, the correlation is partially reduced by the rotational flexibility of this benzene ring, which is characterized by multiple possible dihedral angles ξ (see Fig. 3). Our DFT calculations reveal two energy minima at ξ = 34° and 146° with torsional barriers of ca. 1 kcal/mol for ξ = 0° and 2 kcal/mol for ξ = 90° in a CPCM solvation model, consistent with other examples in literature (*53*, *54*). The minima of ξ represent a compromise between minimizing the steric hindrance of the ortho-substituents in a fully coplanar arrangement of the two benzene rings and avoiding the loss of conjugation in a perpendicular biphenyl orientation. The barriers are sufficiently high to justify modeling the system as a mixture of the two rotamers with equal weights (see details in fig. S8).

To simulate the traces, we applied our theoretical model to the NEEM pulse sequence, which yielded the following spin order mixing amplitude for a system with *N* nuclei

$$m\left(X\to X;\tau_{\text{RF}}\right)=2^{-|X|}\sum_{s\left(X\right)}\text{cos}p\Phi \prod_{k\notin X}\text{cos}\left(\omega_{k}\cdot s\tau_{\text{RF}}\right)$$ (17)

where $\Phi =\phi_{1}+\phi_{2}-2\psi$ (Fig. 4A). By plugging this result in Eq. 15, we find for a system with two nuclear spins

$$\begin{array}{cc} V\left(\tau_{\text{RF}}\right)= & +{\text{cos}}^{2}\frac{A_{1}\tau }{2}{\text{cos}}^{2}\frac{A_{2}\tau }{2}\\ & +{\text{sin}}^{2}\frac{A_{1}\tau }{2}{\text{cos}}^{2}\frac{A_{2}\tau }{2}\text{cos}\text{Φ cos}\left(\omega_{1,2}\tau_{\text{RF}}\right)\\ & +{\text{cos}}^{2}\frac{A_{1}\tau }{2}{\text{sin}}^{2}\frac{A_{2}\tau }{2}\text{cos}\text{Φ cos}\left(\omega_{1,2}\tau_{\text{RF}}\right)\\ & +{\text{sin}}^{2}\frac{A_{1}\tau }{2}{\text{sin}}^{2}\frac{A_{2}\tau }{2}\cdot \frac{1}{2}\left(1+\text{cos}2\Phi \right) \end{array}$$ (18)

This result predicts that one-spin terms (the second and the third lines) are modulated with the nuclear coupling frequency, while the two-spin term (the fourth line) is not. We simulated the NEEM traces based on Eq. 18, and the result is shown in Fig. 4C in red lines. We were able to reproduce the shape of traces in the TD and all distinct features in the FD spectra. Equation 18 predicts τ-dependent blind spot factors that lead to a nonuniform scaling of the observed nuclear spectrum. Therefore, in a general case, NEEM spectra deviate from the classical Pake pattern (*55*), depending on the relative orientation of hyperfine and nuclear coupling tensors defined by the angle Θ (Fig. 4C and fig. S16). In all conformers of **RF**_**2**_, Θ is close to right angle, and we accordingly find that the orientation-averaged NEEM spectrum is close to the Pake pattern.

We noted above that NEEM is an experiment with a variable total nuclear evolution time. Consequently, the traces are affected by relaxation. In (*29*), it was reported for a nitroxide and in (*43*) for Gd(III), that the decay of the nuclear coherences ${\hat{S}}_{z}{\hat{I}}_{x}$ in TD ENDOR experiments is dominated by the electron *T*_1_ relaxation, which is analogous to scalar relaxation of the second kind in NMR. Since fast electron relaxation leads to broadening of nuclear spectra, traces were recorded at 20 K to minimize this effect. Nevertheless, in our simulation, we had to apply an exponential decay $\text{exp}\left(-2a\tau_{\text{RF}}\right)$ to the traces to match the experiment. The best agreement was achieved for *a* = 0.37 ms^−1^, which strongly exceeds the value expected from the electron *T*_1_ (*a* ≈ 0.02 ms^−1^, see section S4.2). To get more insight into origin of this decay, we performed a reference experiment with a singly fluorinated compound with the similar nitroxide-^19^F distance [model compound **1** in (*31*), see section S7]. The data showed that the nuclear echo decay at 20 K is similar to the decay function applied here for **RF**_**2**_. Therefore, there appears to be a relaxation contribution in the nuclear echo decay beyond the simple model *T*_2,*n*_ ≈ 2*T*_1,e_ (*29*, *43*, *56*) that still needs to be established and contributes to the NEEM linewidth. Such relaxation reduces the upper limit of detectable distances in NEEM. However, the available length of the mixing block must also be considered, which is limited by the electron longitudinal relaxation and hyperfine spectral diffusion (*57*). The NEEM traces exhibit high modulation depths (80 to 90%), potentially enabling experiments at concentrations as low as 10 μM (section S8 and fig. S19). In this context, we note that heteronuclear distance measurements are enabled by TD triple-resonance experiments, e.g., SEDOR-ENDOR (*35*). More advanced multiple-resonance techniques exploiting similar principles as NEEM were used to measure the nuclear-nuclear interactions of hyperfine-shifted ^13^C in spin-qubit networks (*58*, *59*).

Last, we note in Eq. 18 that the detection of the nuclear echo in the in-phase channel only is expected to be robust against small nuclear resonance offsets, caused either by large chemical shift or RF frequency offsets, as long as the RF pulses cover the entire ENDOR spectrum. We explicitly tested this for NEEM and PDHS with **RF**_**2**_ by implementing the quadrature detection of the nuclear echo. The quadrature component was acquired by shifting the phase of the read-out pulse by 90°. The results are presented in section S9 and show no significant changes in the spectra.

### Detection of double quantum coherences in PDHS with RF_2_ (*N* = 2)

**Fig. 5.**
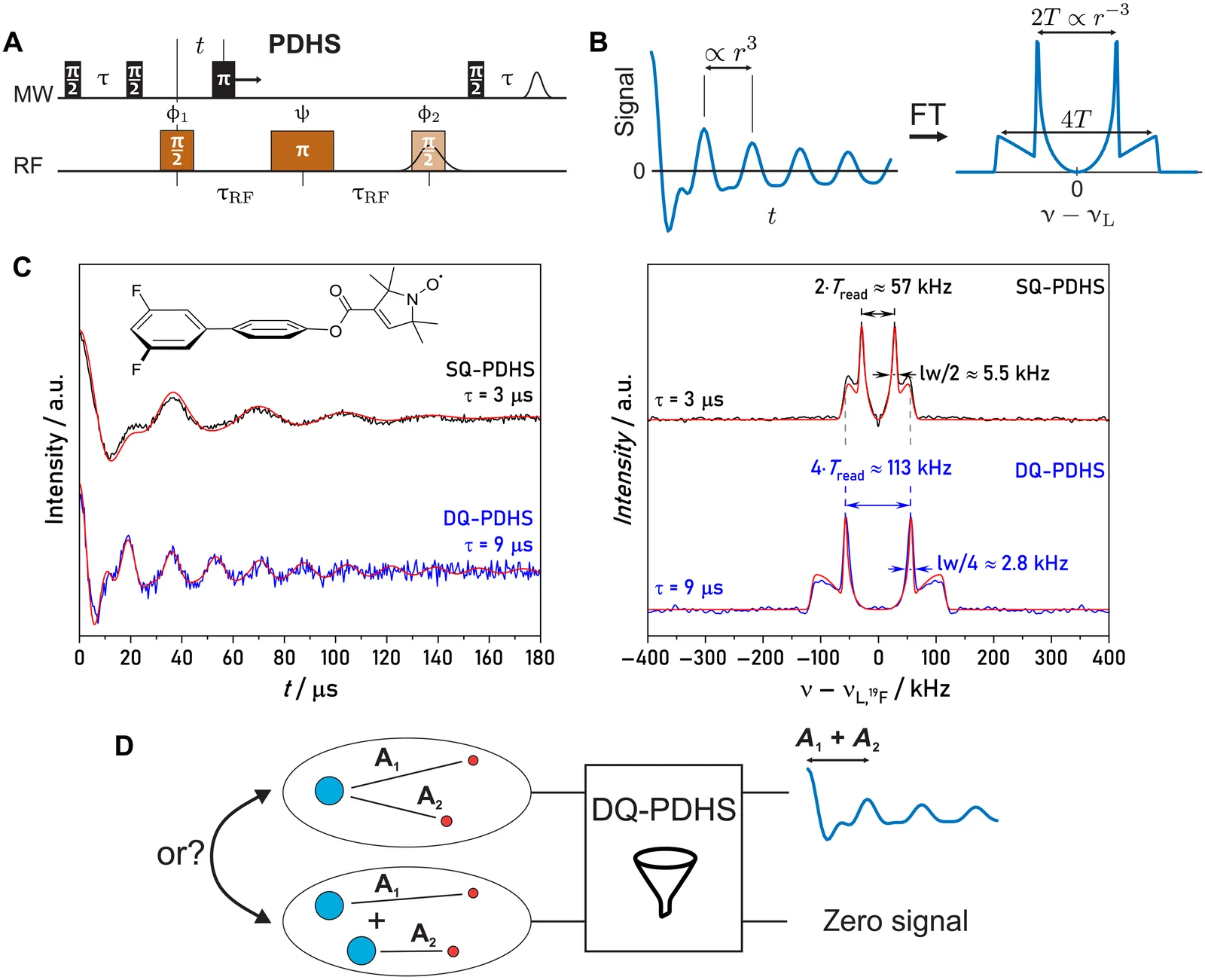
PDHS experiments of RF_2_. (**A**) PDHS pulse sequence. The delay *t* is incremented. Delay τ_RF_ is constant and determines the maximal modulation interval. (**B**) Synthetic PDHS data in a single-nucleus case. Powder-averaged time trace (left) and its real Fourier transform (right). The spectrum is a product of the Pake pattern centered at the nuclear Larmor frequency and the blind spot function (Mims Pake pattern). The latter causes decreased intensity in the middle and elevated shoulders. Characteristic features are marked in the time and FDs. *r* is the electron-nuclear distance and parameter $T=\mu_{0}/\left(4\pi h\right)\cdot g_{e}g_{n}\mu_{B}\mu_{N}/r^{3}$ is the perpendicular principal value of the hyperfine dipolar tensor. (**C**) Left: PDHS sum time traces of compound **RF**_**2**_ recorded at 50 K with a single- and double-quantum filter phase cycle with 12 ns MW π/2 pulses, 4 μs RF π/2 pulses, τ = 3 μs (SQ), τ = 9 μs (DQ), τ_RF_ = 220 μs, 6 ms srt, 10 spp, and the MW π pulse was stepped in 480 ns steps. Simulations using a mono-Gaussian distance distribution are shown as solid red lines (see also table S5). Right: PDHS spectra obtained via Fourier transform of the corresponding time traces. (**D**) Schematic illustrating the ability of DQ-PDHS to distinguish between two nuclei coupled to the same electron (top) and two electron-nucleus pairs (bottom) by correlating hyperfine couplings (*A*_1_ and *A*_2_).

Information on nitroxide-^19^F distances and their distributions can be obtained using the recently introduced PDHS experiment (*31*), which enables the determination of hyperfine couplings with kHz resolution. By refocusing line-broadening interactions, such as heteronuclear dipolar coupling and chemical shift, PDHS substantially enhances the resolution of hyperfine couplings and their distribution compared to the standard FD ENDOR technique (Fig. 5B). The PDHS pulse sequence is shown in Fig. 5A.

In PDHS, a nuclear spin echo is detected that is modulated by an additional MW π pulse, stepped in time, while the time delay τ_RF_ is fixed. Using our theoretical framework, we derived the following expression for the mixing factor in a PDHS experiment $\left(\Phi =\phi_{1}+\phi_{2}-2\psi \right)$

$$\begin{array}{c} m\left(X\to X;t,\tau_{\text{RF}}\right)=\\ -2^{-|X|}\sum_{s\left(X\right)}\text{cos}p\text{Φ cos}\left(A\cdot st\right)\prod_{k\notin X}\text{cos}\left(\omega_{k}\cdot s\tau_{\text{RF}}\right) \end{array}$$ (19)

In a system with a single nucleus (*N* = 1), the delay time τ_RF_ only determines the maximum evolution time, since no dependence on τ_RF_ apart from relaxation is expected

$$V\left(t;\tau_{\text{RF}}\right)={\text{cos}}^{2}\frac{A_{1}\tau }{2}+{\text{sin}}^{2}\frac{A_{1}\tau }{2}\text{cos}\text{Φcos}A_{1}t$$ (20)

Note that the modulation frequency in PDHS is twice as large as in FID-TD ENDOR or FD ENDOR (*3*). Consequently, in powder spectra, the read-out splitting in the PDHS spectrum corresponds to 2*T*, where *T* is the perpendicular component of the hyperfine dipolar tensor (Fig. 5B). Overall, the detected PDHS spectrum is a product of the Pake pattern and the Mims blind spot function, which we refer to as Mims Pake pattern.

In multinuclear systems, also homonuclear dipolar couplings modulate the nuclear echo; therefore, we expect a more complex signal composition. For two nuclei, the signal is found as

$$\begin{array}{cc} V\left(t;\tau_{\text{RF}}\right)= & +{\text{cos}}^{2}\frac{A_{1}\tau }{2}{\text{cos}}^{2}\frac{A_{2}\tau }{2}\\ & +{\text{sin}}^{2}\frac{A_{1}\tau }{2}{\text{cos}}^{2}\frac{A_{2}\tau }{2}\text{cos}\text{Φ cos}A_{1}t\;\text{cos}\left(\omega_{1,2}\tau_{\text{RF}}\right)\\ & +{\text{cos}}^{2}\frac{A_{1}\tau }{2}{\text{sin}}^{2}\frac{A_{2}\tau }{2}\text{cos}\text{Φ cos}A_{2}t\;\text{cos}\left(\omega_{1,2}\tau_{\text{RF}}\right)\\ & +{\text{sin}}^{2}\frac{A_{1}\tau }{2}{\text{sin}}^{2}\frac{A_{2}\tau }{2}\cdot \frac{1}{2}\left(\text{cos}\left(A_{1}-A_{2}\right)t+\text{cos}2\text{Φ cos}\left(A_{1}+A_{2}\right)t\right) \end{array}$$ (21)

In addition to the modulation with the two hyperfine coupling frequencies, we now find a modulation with combination frequencies. This part of the signal is generated from a nuclear two-spin coherence term $4{\hat{S}}_{z}{\hat{I}}_{1,y}{\hat{I}}_{2,y}$ and can therefore be called double-/zero-quantum (DQ/ZQ) contribution (*33*).

We performed PDHS experiments on the model compound **RF**_**2**_ with the SQ- and DQ-filter phase cycling protocol [see (*48*) and the “Experimental details” section]. Note that the initially proposed phase cycling protocol (*31*) for removing background signal in PDHS is equivalent to an SQ filter, thus it cancels ZQ and DQ contributions. Further experimental details are provided in the experimental section. We took the weighted sum of the three traces recorded at three different observer positions corresponding to the principal orientation of the *g* tensor (see fig. S11) to minimize distortions from orientation selection. The resulting sum time traces and their Fourier transform are shown in Fig. 5C. For the PDHS spectrum acquired with an SQ-filter, we observe a single splitting of ≈ 57 kHz. This splitting corresponds to a nitroxide-^19^F distance of 13.8 Å, which is in agreement with the DFT-predicted values of 13.6 and 14.2 Å. The two close distances are not resolved, likely due to molecular flexibility. However, the peaks are almost twice as broad compared to a model compound containing a single fluorine nucleus at a similar distance [lw/2 = 5.5 kHz in **RF**_**2**_ and lw/2 = 2.8 kHz for compound **1** in (*31*)]. NEEM data on model compound **RF**_**2**_ demonstrated that the nuclear spin echo can be strongly depleted at specific values of τ_RF_. We measured SQ-filtered PDHS with different τ_RF_ values (fig. S23) and observed no PDHS signal at τ_RF_ = 420 μs as expected from the measurements shown in Fig. 4B (see also section S11). While the choice of τ_RF_ in singly fluorinated systems only balances between frequency resolution and relaxation losses, it should be more carefully considered in multispin systems.

The PDHS time trace acquired with a DQ filter shows modulation with sum frequencies corresponding to a splitting of ≈113 kHz. As confirmed by SQ-PDHS, the two fluorines show almost equal hyperfine couplings, leading to the sum frequency being approximately the double frequency. Detection of double modulation frequencies could aid in determining long electron-nuclear distances, as the resolution limit is increased. Another specificity predicted for DQ-PDHS is the change in the τ-scaling factor, i.e., the blindspot function, clearly evident in the spectrum acquired with a DQ filter. The central hole in the PDHS spectrum exhibits an almost square shape due to the multiplication of the Pake pattern by a factor of $\approx {\text{sin}}^{4}\frac{A\tau }{2}$ (see Eq. 21). The scaling factor of the DQ contribution can also be noted in the τ dependence of the signal. We exemplify such scaling experimentally in section S10. The presented PDHS time trace with an SQ-filter was acquired with τ = 3 μs. At the same time, to achieve a sufficient signal-to-noise ratio for DQ-PDHS, a longer τ > 6 μs was necessary, as explained in the “Generation block” section (Fig. 2B). While SQ-PDHS contains the distance information of both fluorines, it cannot distinguish whether the signal arises from the same nucleus in different conformations or from two separate nuclei. DQ-PDHS allows to distinguish these two cases by directly correlating hyperfine couplings (Fig. 5D).

In the next step, we examined whether distance distributions can be extracted from the obtained PDHS traces. To account for multispin effects, we modified the dipolar kernel functions using our analytical solution (Eq. 21) for PDHS involving two fluorine nuclei. Using a Gaussian model for the distance distribution $P\left(r\right)=\frac{1}{\sqrt{2\pi }\sigma }\text{exp}\left(-\frac{{\left(r-\left\langle r\right\rangle \right)}^{2}}{2\sigma^{2}}\right)$, we fitted the SQ- (⟨r⟩ = 13.54 Å, σ = 0.34 Å) and DQ-filtered (⟨r⟩ = 13.72 Å, σ = 0.17 Å) PDHS traces. The fit results are shown in Fig. 5C. More details are provided in table S5.

We note the quality of trace fitting achieved with a simple model for P(r). The main difference between the two experiments (SQ- versus DQ-PDHS) is the width of the distance distributions. In SQ-PDHS, we observe a broader distance distribution as contributions from two fluorines with close mean distances are overlaid in the additive approximation (see the “Additive approximation at short τ” section). In DQ-filtered PDHS, we detect the sum frequency $\left(A_{1}+A_{2}\right)$ whose fluctuation is smaller than the sum of the individual terms’ variation. This is due to the partial correlation between $A_{1}$ and $A_{2}$ in a rigid structure like **RF**_**2**_. The distance distribution in DQ-PDHS, therefore, should be interpreted and analyzed with caution.

### Correlation of the hyperfine and homonuclear dipolar coupling tensors

**Fig. 6.**
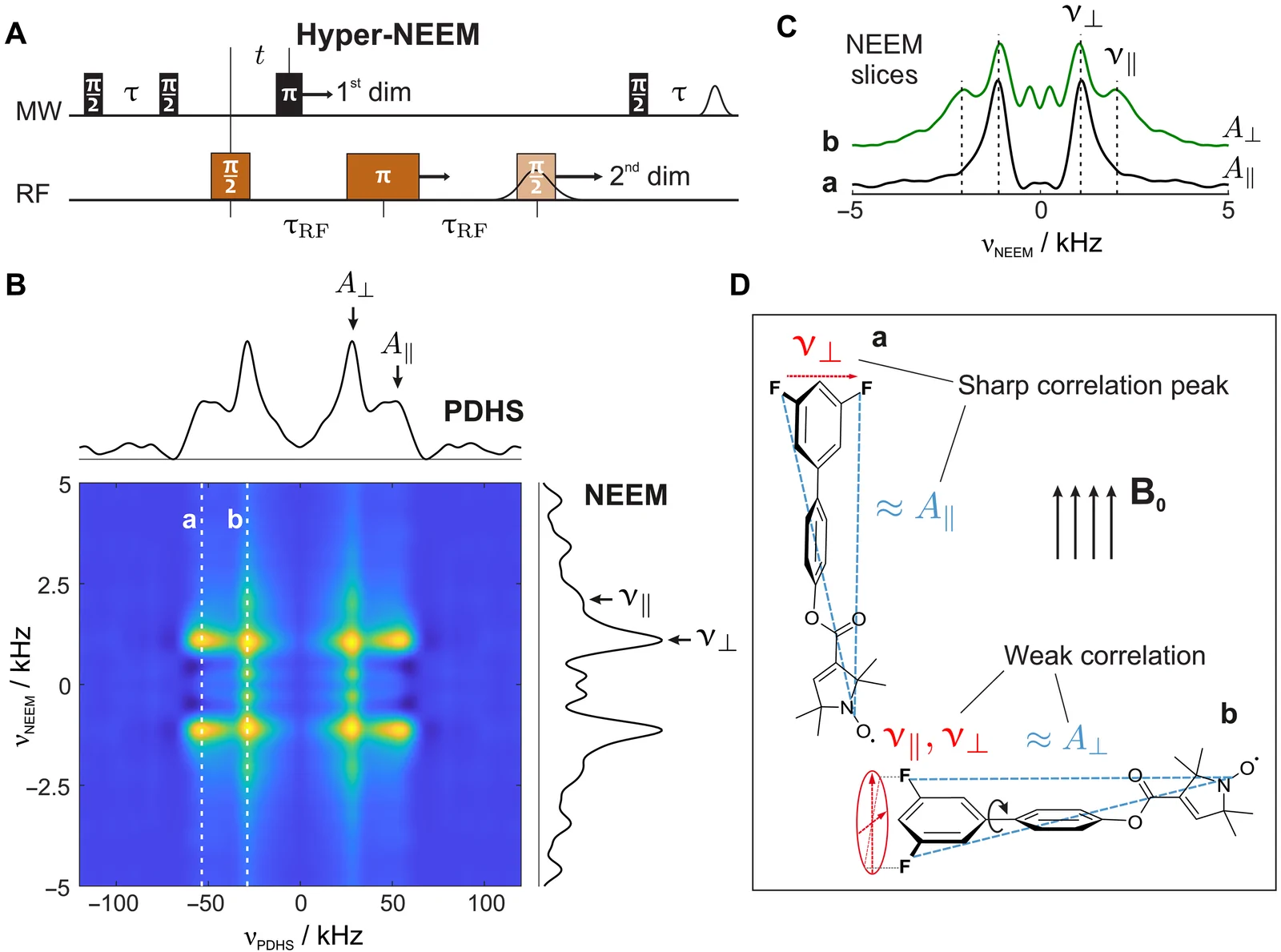
2D PDHS-NEEM correlation spectroscopy. (**A**) Hyper(fine)-NEEM pulse sequence scheme. The delay *t* is incremented in the first dimension and delay τ_RF_ is incremented in the second dimension. (**B**) NEEM-PDHS correlation spectrum of **RF**_**2**_, measured at 20 K with 12 ns MW π/2 pulses, 4 μs RF π/2 pulses, τ = 3 μs, 60 ms srt, 6 spp at the *g_y_* observer positions. *t* was incremented in 700 ns steps. τ_RF_ was incremented in 12 μs steps. Characteristic frequencies are marked on the projections. A minimal τ_RF_ of 150 μs was chosen for the experiments. (**C**) NEEM slices with ν_PDHS_ = *A*_∥_ ≈ 57 kHz (a) and ν_PDHS_ = *A*_⊥_ ≈ 28 kHz (b). Characteristic frequencies are marked. (**D**) Chemical structure of **RF**_**2**_ in two characteristic orientations. Blue lines indicate the orientation of the hyperfine coupling tensor. Red lines indicate the orientation of the internuclear coupling tensor. In case (**a**, *A*_∥_), the ring mobility of the biphenyl residue does not change the nuclear coupling, while in (**b**, *A*_⊥_), rotation leads to variation of the nuclear dipolar coupling.

At each fixed delay τ, the PDHS signal in multispin systems explicitly depends on two time delays: *t* and τ_RF_. This allows us to correlate hyperfine coupling and nuclear dipolar coupling by recording two-dimensional (2D) data, as shown in Fig. 6A for compound **RF**_**2**_.

The projections of the 2D-Fourier transform spectrum onto the coordinate axes are the PDHS and NEEM spectra (Fig. 6B). We note that the NEEM spectrum is slightly distorted due to lack of the first time points in the NEEM dimension, as a certain starting value of τ_RF_ is required to record the PDHS time traces. The 2D-spectrum is mirror-symmetric; therefore, we focus on the first quadrant. We find a correlation peak (*A*_∥_, ν_⊥_) that corresponds to molecules oriented along the magnetic field (see slice **a** in Fig. 6C and structure **a** in Fig. 6D). In compound **RF**_**2**_, the nuclear dipolar tensor is oriented nearly perpendicular to the hyperfine tensors, hence the correlation of a parallel HFC with a perpendicular NDC tensor component. The slice of ν_PDHS_ = *A*_⊥_ (slice **b** in Fig. 6C) contains both ν_⊥_ and ν_∥_ because multiple molecular orientations contribute to this coupling and because of the difluoro-phenyl ring flipping. Overall, such correlation data can be valuable for studying systems with partial flexibility.

### Nitroxide-^19^F and ^19^F-^19^F distance distributions in RFF′

**Fig. 7.**
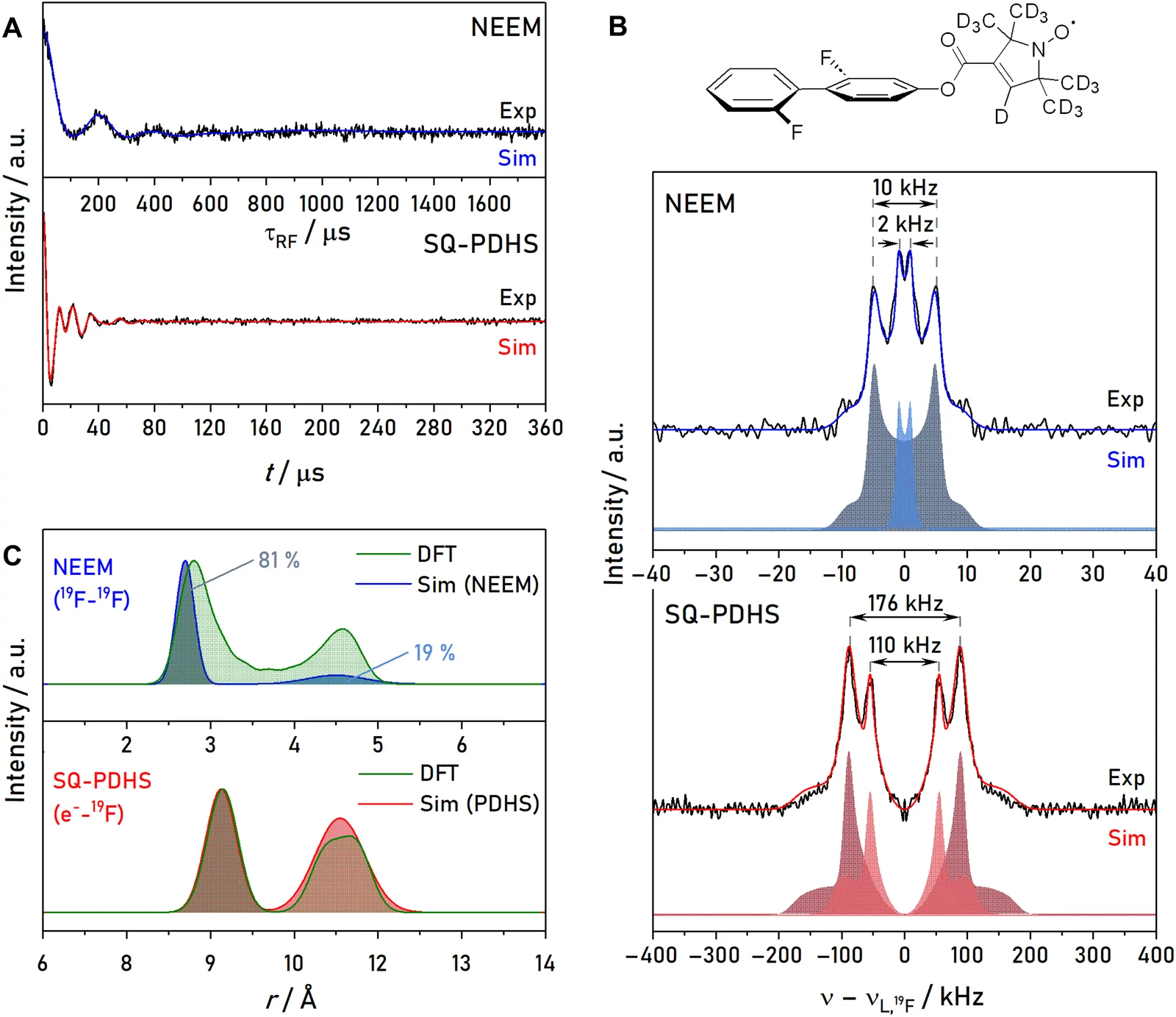
PDHS and NEEM experiments of model compound RFF′. (**A**) NEEM and SQ-filtered PDHS time trace of **RFF′** recorded at 50 K with 12 ns MW π/2 pulses, 4 μs RF π/2 pulses, τ = 2 μs (PDHS), τ = 3 μs (NEEM), 6 ms srt, 10 spp at the *g_y_* observer position. *t* was incremented in 300 ns steps for PDHS. τ_RF_ was incremented in 3 μs steps for NEEM. Simulations of the NEEM and PDHS traces with bi-Gaussian distance distributions are shown as blue and red solid lines, respectively. The model parameters are given in Table 1. (**B**) Fourier transform of the NEEM and PDHS time traces and their simulations. Shaded areas indicate separate contributions of the simulation marked in the distance distributions. (**C**) ^19^F-^19^F distance distribution from simulation of NEEM and DFT-predicted distribution (upper panel). Nitroxide-fluorine distance distribution from simulation of PDHS and DFT-predicted distribution (lower panel).

To examine a case of a more complex nuclear distance distribution, we conducted experiments with the compound **RFF′** (Figs. 3 and 7). Rotation around the single C−C bond of the biphenyl fragment, described by the dihedral angle ξ, strongly affects the ^19^F-^19^F distance (Fig. 3 and fig. S10). A combined approach using PDHS and NEEM enables a characterization of both the nitroxide-^19^F and ^19^F-^19^F distance distributions.

SQ-filtered PDHS (Fig. 7, A and B) reveals two main peaks, assigned to the proximal and distal fluorine atoms within the biphenyl unit. The DQ-PDHS confirms that these two fluorines indeed belong to the same radical, as displayed in fig. S25. Simulations employing a bimodal Gaussian model yielded two mean distances of 9.3 and 10.9 Å (Fig. 7C and Table 1). The line width reduction in PDHS enables the clear separation of these two close distances. For comparison, FD ENDOR measurement on **RFF′** reveals only a single splitting in the ENDOR spectrum (fig. S24). However, the smallest resolvable difference in distances in PDHS depends on the flexibility of the specific molecule, i.e., its distance distribution and the mean distance between the two. It is unclear a priori, whether flipping of the distal benzene is expected in this molecule. Intramolecular fluorine-fluorine and fluorine-proton steric and electrostatic interactions, as well as interaction with solvent, may shift the relative energies of the conformers beyond the thermal energy, thereby strongly depopulating some of those. We addressed this question experimentally by probing the distribution of fluorine-to-fluorine distances via NEEM. The NEEM spectrum shown in Fig. 7B displays two splittings corresponding to distances of 2.4 and 5.2 Å, evidencing the internal rotation of the biphenyl fragment. Simulations of the NEEM time traces using a bimodal Gaussian distance model suggest a population ratio of approximately 80:20. To validate the results from both PDHS and NEEM, relaxed surface scans with respect to the dihedral angle ξ were carried out to evaluate the nuclear-nuclear distance distribution. DFT calculations reveal two energy minima at ξ ≈ 60° and ξ ≈ 130°, with an energy difference of 1.8 kJ/mol. The resulting energy profile was used to predict the distance distributions presented in Fig. 7C. Further details on the DFT analysis are provided in section S3. For both experiments, we observe a good qualitative agreement between the experimentally derived and DFT-predicted distance distributions. Notably, the DFT calculations predict a somewhat higher weight for the long internuclear distance. The distributions in Fig. 7C are based on Boltzmann factors calculated for room temperature. However, if the equilibrium between rotamers adjusts to the noninstantaneously temperature fall upon freeze-quenching of the EPR sample, the experimental distance distribution would correspond to a lower temperature. Given the low barrier (≈1 kcal/mol, see section S3.2), it is likely that the actual temperature represented in EPR data is close to the freezing point of our solvent mixture, below which the solvent viscosity grows fast. By taking *T* = 200 K, where our solvent mixture still remains liquid (*60*), we can improve the agreement. Further estimations will be sensible only if the energy difference is calculated more precisely. Overall, the PDHS and NEEM experiments provide complementary, structural information that allows the determination of the dihedral angle ξ and its distribution, information that is inaccessible by standard FD ENDOR methods.

**Table 1.** PDHS and NEEM distance distributions of RFF′. Fitting parameters of SQ-PDHS and SQ-NEEM traces acquired for model compound **RFF′** (Fig. 7). $\langle r_{i}\rangle$ (*i* = 1, 2) are the mean distances, $\sigma_{i}$ (*i* = 1, 2) the SDs, and $w_{i}$ (*i* = 1, 2) are the relative weights in the bi-Gaussian model used in the simulations: $P\left(r\right)=\frac{w_{1}}{\sqrt{2\pi }\sigma_{1}}\cdot \text{exp}\left(-\frac{{\left(r-\langle r_{1}\rangle \right)}^{2}}{2\sigma_{1}^{2}}\right)+\frac{1-w_{1}}{\sqrt{2\pi }\sigma_{2}}\cdot \text{exp}\left(-\frac{{\left(r-\langle r_{2}\rangle \right)}^{2}}{2\sigma_{2}^{2}}\right)$.

Experiment	$w_{1}$	$\langle r_{1}\rangle$ /Å	$\sigma_{1}$/Å	$\langle r_{2}\rangle$/Å	$\sigma_{2}$/Å
PDHS	0.45 ± 0.05	9.30 ± 0.02	0.21 ± 0.02	10.90 ± 0.02	0.34 ± 0.04
NEEM	0.81 ± 0.02	2.69 ± 0.01	0.11 ± 0.01	4.31 ± 0.01	0.35 ± 0.03

### Distance measurements with trifluoromethyl groups CF_3_ (*N* = 3)

Having a quantitative understanding of the ENDOR spectroscopy for systems with two fluorine nuclei, we can proceed to the next advanced case. Here, we examine what kind of information can be obtained from MQ TD ENDOR spectroscopy with a CF_3_ group, which was introduced and is relevant for distance measurements (*22*, *23*, *25*, *61*). We first focus on the NEEM effect, which is a prerequisite to optimize a PDHS experiment. For three spins (*N* = 3), the analytical expression for NEEM can also be obtained using our theoretical model (eq. S58 in section S1.10.1). In contrast to the *N* = 2 case, the signal in *N* = 3 is modulated by combination frequencies of the three nuclear dipolar couplings $\omega_{1,2}$, $\omega_{1,3}$, and $\omega_{2,3}$ with different amplitudes determined by preparation time τ. The single-quantum contribution contains both sum and difference of nuclear frequencies because of the product of two cosines in eq. S58, and the double-quantum part has only sum frequencies. The zero-quantum contribution, which appears with the same amplitude prefactor, is eliminated by SQ- and DQ-filter phase cyclings.

**Fig. 8.**
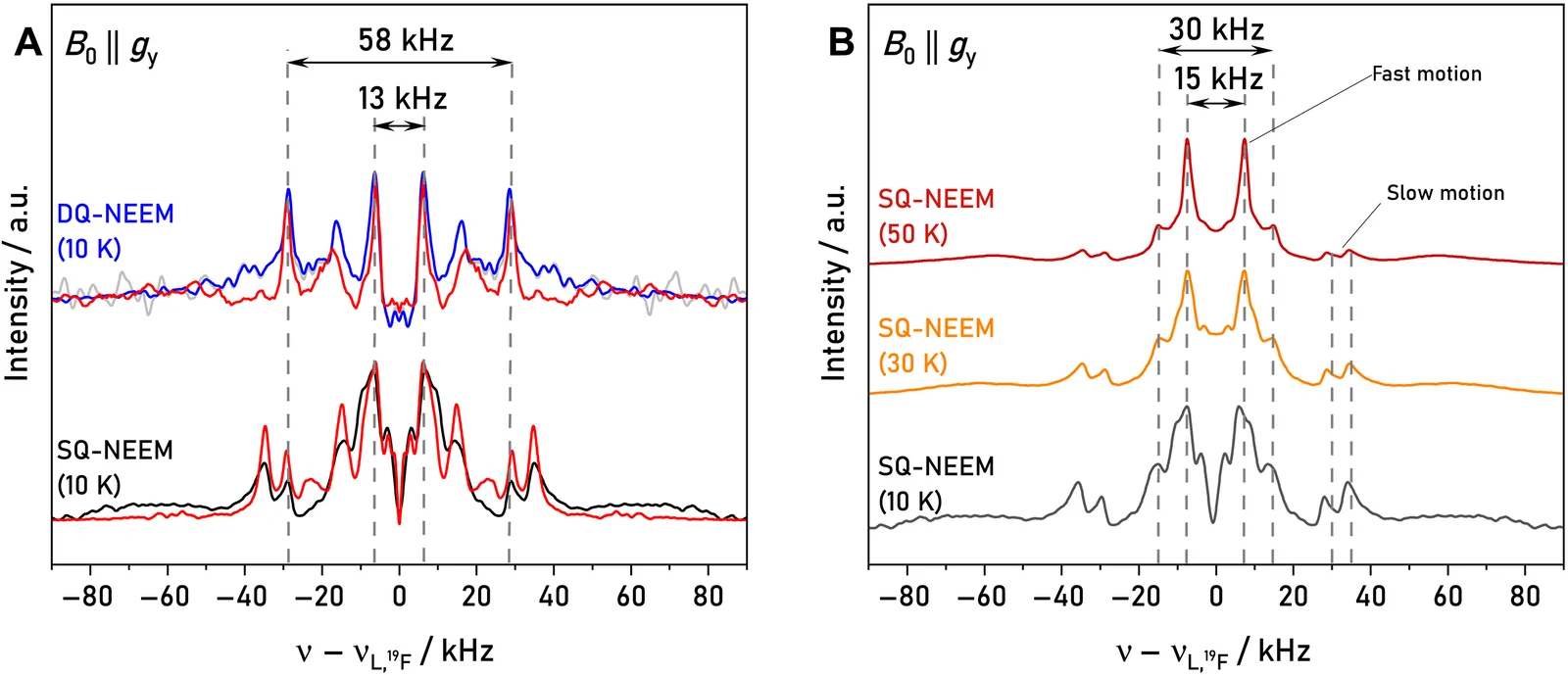
NEEM experiments of model compound RCF_3_. (**A**) Experimental SQ- and DQ-NEEM spectra of compound **RCF**_**3**_ recorded at 10 K with 12 ns MW π/2 pulses, 4 μs RF π/2 pulses, τ = 3 μs (SQ), τ = 5 μs (DQ), 500 ms srt, 2 spp at the *g_y_* observer positions. τ_RF_ was incremented in 3 μs steps. Simulations (red lines) were performed by propagating the density matrix, see text. (**B**) Temperature series of SQ-NEEM spectra (τ = 3 μs) acquired at 10, 30, and 50 K. Shot repetition times (srt) were 500, 25, and 10 ms, respectively. Other experimental parameters are same as in (A). The spectra at 30 and 50 K demonstrate signatures of CF_3_ groups undergoing fast uniaxial rotation, and the fraction of such mobile groups grows with temperature.

The SQ-NEEM and DQ-NEEM traces measured for compound **RCF**_**3**_ at 10 K are shown in figs. S17, S28, and S29. The corresponding spectra are shown in Fig. 8A. The spectra reveal multiple resolved features, whereby SQ-NEEM includes the DQ-NEEM spectrum and several additional features. Simulating NEEM spectra of a CF_3_ group is challenging because fluorines in a CF_3_ group are closer to each other than in **RF**_**2**_. Consequently, the homonuclear coupling (ν_⊥_ = 10.1 kHz from DFT) is larger than the difference in hyperfine couplings. The high-field approximation is not accurate in this case, and the pseudo-secular part of the nuclear dipolar interaction must be included

$${\hat{H}}_{\text{hom}.\text{ND}}=\sum_{j<k}\omega_{j,k}\left({\hat{I}}_{j,z}{\hat{I}}_{k,z}-\frac{{\hat{I}}_{j}^{+}{\hat{I}}_{k}^{-}+{\hat{I}}_{j}^{-}{\hat{I}}_{k}^{+}}{4}\right)$$ (22)

With this extension, no simple analytical solution exists for the case of three hyperfine-coupled nuclei. The use of the analytical solution with HFA (eq. S58) and a heuristic correction to the effective dipolar couplings (eq. S78), accounting for the level shift due to the flip-flop term, yields only poor agreement with experiment (see section S15.2). Simulations based on numeric propagation of the density matrix with the spin Hamiltonian $\hat{H}={\hat{H}}_{\text{HF}}+{\hat{H}}_{\text{hom}.\text{ND}}$ reproduce the experimental spectra (see Fig. 8A and section S15) and indicate that the ^19^F-^19^F coupling deviates from the DFT-predicted value by about 3 to 4%. Simulations also show that the narrow spectral features correspond to molecular orientations in which the magnetic field is perpendicular to the nitroxide-CF_3_ axis, making the three hyperfine couplings nearly identical and rendering the NEEM spectrum close to a pure dipolar NMR spectrum of CF_3_. (*62*) In **RF**_**2**_ and **RFF′** cases, the NEEM effect is driven by the allowed nuclear transitions and thus expected to be field-independent. For **RCF**_**3**_,nonnegligible pseudo-secular coupling together with chemical shift might cause field dependence of NEEM.

Until now, we have discussed the NEEM spectra of **RCF**_**3**_ assuming a fixed geometry. However, as previously mentioned, CF_3_ groups can undergo uniaxial rotation that is abundantly observed by ^19^F-NMR at room temperature (*63*). FD ENDOR experiments with **RCF**_**3**_ at high fields suggested that a temperature of 50 K is sufficient for activating the CF_3_ rotation (*44*). This is related to an exceptionally low torsional barrier (≈3 kJ mol^−1^) of the para-substituted CF_3_ group (*44*). The motion appears to occur via the mechanism of thermally activated sudden jumps, and it can be recognized as stochastic intramolecular reorientation rather than spinning. Here, we report the notable temperature dependence of NEEM spectra in the range of 10 to 50 K (Fig. 8B). NEEM data at 10 K are discussed above, and we refer to it as a frozen limit. At higher temperatures, the experimental spectra can be fitted as a mixture of the frozen 10 K pattern and a sharp Pake-like doublet with a splitting of ≈15 kHz (fig. S30), which we assign to mobile CF_3_ groups. Current simulations, based on the assumption of fast uniaxial motion, overestimate this splitting and, therefore, do not yet capture the full observed line shape. In fast motion regime, the rate constant of the CF_3_ reorientation, *k*, must exceed the difference of nuclear-nuclear couplings within CF_3_. This results in estimation *k* > 2π·10 kHz ≈ 63 ms^−1^. We were unable to reliably detect intermediate motion states. On the one hand, their spectra can be broad, similar to coalescence peaks in exchange spectroscopy, and thus not resolved. On the other hand, a sharp transition from slow to fast motion regimes can occur due to a low torsional barrier. These observations indicate that a more refined theoretical treatment of CF_3_ dynamics will be required to achieve full quantitative agreement, which goes beyond the scope of this work.

### Detection of single and MQ coherences in PDHS with CF_3_ (*N* = 3)

**Fig. 9.**
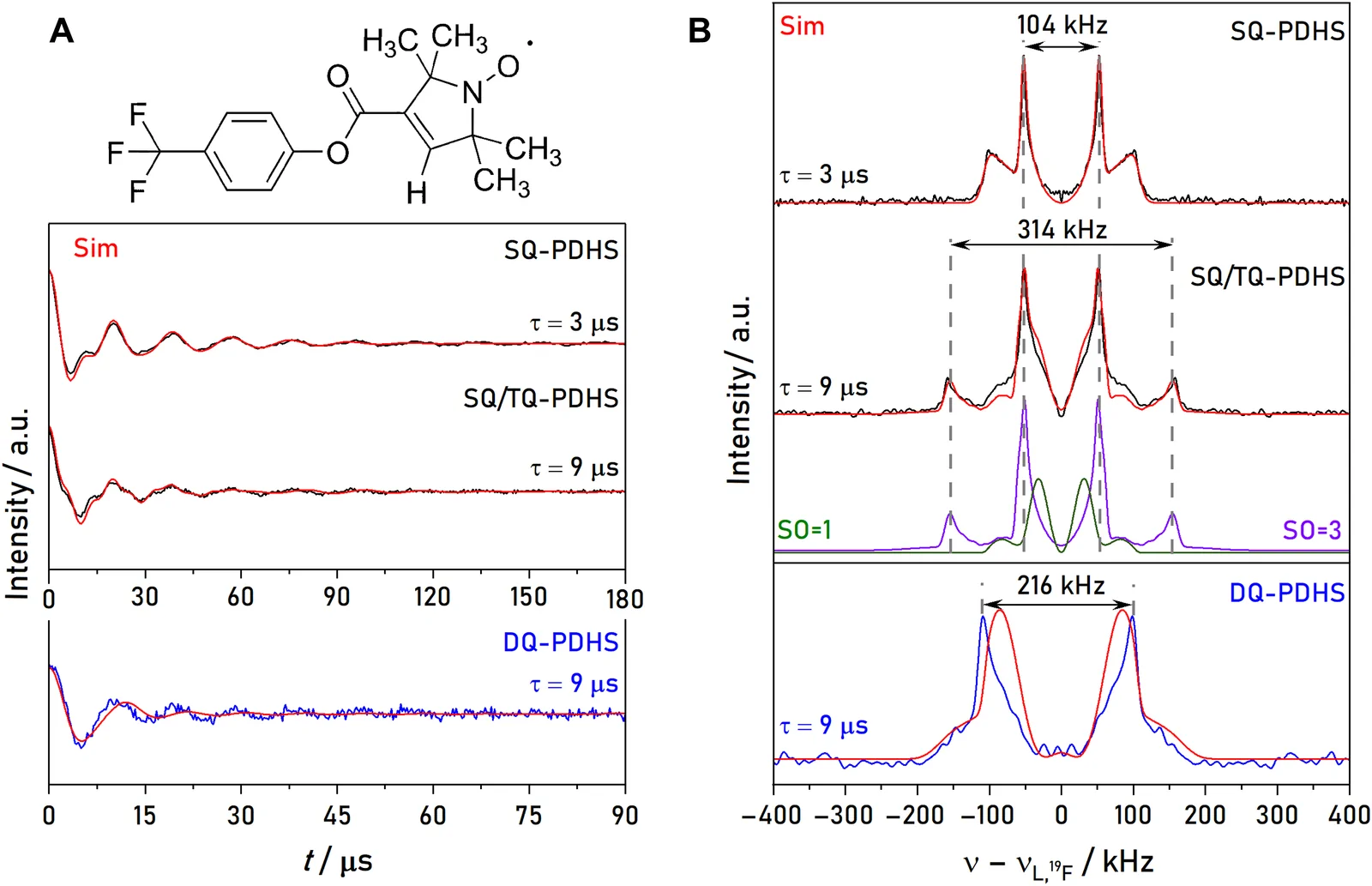
PDHS experiments of model compound RCF_3_. (**A**) SQ/TQ and DQ-filtered PDHS traces of model compound **RCF**_**3**_ measured at 10 K with 12 ns MW π/2 pulses, 4 μs RF π/2 pulses, τ = 9 μs, τ_RF_ = 220 μs, 500 ms srt, 2 spp, and the MW π pulse was stepped in 480 ns (SQ) or 240 ns (DQ) steps. Simulations are shown as solid red lines and the simulation parameters are summarized in table S6. (**B**) Fourier transform of the experimental and simulated time traces. Decomposition of the spectrum τ = 9 μs into pure 1-spin (SO = 1) and 3-spin (SO = 3) contributions is shown in green and purple, respectively.

In analogy to model compound **RF**_**2**_, we performed PDHS experiments with SQ- and DQ-filter phase cycle for model compound **RCF**_**3**_ at three observer positions in the EPR spectrum and summed them up (Fig. 9A). We first recorded PDHS traces using a short τ = 3 μs value, where the additive approximation was expected to be valid. We observe a typical Mims Pake pattern with a main splitting of 2*T* ≈ 104 kHz (see also Fig. 5B) that can be assigned to the nitroxide-^19^F distance of 11.3 Å, in good agreement with the DFT. As multispin effects are suppressed under these conditions, the PDHS trace can be simulated assuming the CF_3_ group as a single fluorine nucleus (Eq. 29). This is a very important result, which shows that the complicated situation of a multispin probe can be reduced to a simple case. Nevertheless, with increasing τ values, this approximation becomes progressively less valid. To demonstrate this, we recorded PDHS traces with a τ = 9 μs value (Fig. 9A). In SQ-PDHS, we now observe more frequencies, corresponding to additional contributions in FT spectrum. In addition to the case τ = 3 μs, a splitting of ≈ 314 kHz is present which would correspond to a distance of *r* = 7.8 Å, structurally not feasible in compound **RCF**_**3**_. It is the triple-quantum (TQ) contribution, which is not filtered out by the used phase cycle protocol. The value of this splitting is the sum of the hyperfine couplings of three fluorines, which are almost equal in the case of CF_3_. The presence of this signal is expected from the analytical expression for PDHS with three fluorines

$$\begin{array}{c} V\left(t;\tau_{\text{RF}}\right)=C_{1}^{2}C_{2}^{2}C_{3}^{2}\\ +S_{1}^{2}C_{2}^{2}C_{3}^{2}\text{cos}\text{Φcos}A_{1}t\text{cos}\left(\omega_{1,2}\tau_{\text{RF}}\right)\text{cos}\left(\omega_{1,3}\tau_{\text{RF}}\right)\\ +C_{1}^{2}S_{2}^{2}C_{3}^{2}\text{cos}\text{Φcos}A_{2}t\text{cos}\left(\omega_{1,2}\tau_{\text{RF}}\right)\text{cos}\left(\omega_{2,3}\tau_{\text{RF}}\right)\\ +C_{1}^{2}C_{2}^{2}S_{3}^{2}\text{cos}\text{Φcos}A_{3}t\text{cos}\left(\omega_{1,3}\tau_{\text{RF}}\right)\text{cos}\left(\omega_{2,3}\tau_{\text{RF}}\right)\\ +S_{1}^{2}S_{2}^{2}C_{3}^{2}\frac{1}{2}\text{cos}2\text{Φcos}\left(A_{1}+A_{2}\right)t\text{cos}\left(\left(\omega_{1,3}+\omega_{2,3}\right)\tau_{\text{RF}}\right)\\ +\text{cos}\left(A_{1}-A_{2}\right)t\text{cos}\left(\left(\omega_{1,3}-\omega_{2,3}\right)\tau_{\text{RF}}\right)\\ +C_{1}^{2}S_{2}^{2}S_{3}^{2}\frac{1}{2}\text{cos}2\text{Φcos}\left(A_{2}+A_{3}\right)t\text{cos}\left(\left(\omega_{1,2}+\omega_{1,3}\right)\tau_{\text{RF}}\right)\\ +\text{cos}\left(A_{2}-A_{3}\right)t\text{cos}\left(\left(\omega_{1,2}-\omega_{1,3}\right)\tau_{\text{RF}}\right)\\ +S_{1}^{2}C_{2}^{2}S_{3}^{2}\frac{1}{2}\text{cos}2\text{Φcos}\left(A_{1}+A_{3}\right)t\text{cos}\left(\left(\omega_{1,2}+\omega_{2,3}\right)\tau_{\text{RF}}\right)\\ +\text{cos}\left(A_{1}-A_{3}\right)t\text{cos}\left(\left(\omega_{1,2}-\omega_{2,3}\right)\tau_{\text{RF}}\right)\\ +S_{1}^{2}S_{2}^{2}S_{3}^{2}\frac{1}{4}\text{cos}3\text{Φcos}\left(A_{1}+A_{2}+A_{3}\right)t\\ +\text{cos}\Phi \left[\text{cos}\left(-A_{1}+A_{2}+A_{3}\right)t+\text{cos}\left(A_{1}-A_{2}+A_{3}\right)t+\text{cos}\left(A_{1}+A_{2}-A_{3}\right)t\right] \end{array}$$ (23)

Here, $C_{i}^{2}={\text{cos}}^{2}\frac{A_{i}\tau }{2}$ and $S_{i}^{2}={\text{sin}}^{2}\frac{A_{i}\tau }{2}$ are shorthand notations of the scaling factors. Notice that the SQ-contribution is additionally modulated by $A_{1}+A_{2}-A_{3}$, etc. We refer to these terms as SQ-contribution in the spin order 3 (SO = 3). Because of the similarity of hyperfine couplings in **RCF**_**3**_, such combinations are close to the fundamental frequencies $A_{1}$, $A_{2}$, and $A_{3}$. Equation 23 also shows that in contrast to the *N* = 2 case, the DQ-contribution in PDHS becomes τ_RF_-dependent and is scaled with $S_{i}^{2}S_{j}^{2}C_{k}^{2}$, leading to a different blind spot function. We note that to obtain high resolution in SQ- and DQ-PDHS with **RCF**_**3**_, relatively large τ_RF_ were used here. The experimental NEEM traces exhibit only shallow decay at these values (see section S6), which allows to choose the τ_RF_ more freely. As in the *N* = 2 case, we used the result in Eq. 23 to construct the dipolar kernels for fitting nitroxide-^19^F distance distributions in PDHS, see “Dipolar kernels for specific experiments” section. The final simulations show good agreement with the experimental data (Fig. 9). The SQ-PDHS spectrum of **RCF**_**3**_ is a sum of single- and three-spin contributions, with characteristic blind spot, i.e., scaling factors. Figure 9B shows the isolated signal contributions corresponding to pure single- and three-spin contributions.

We note that the SQ-PDHS spectrum for τ = 9 μs is dominated by the three-spin contribution, while the single-spin contributions create characteristic shoulders in the center of the spectrum. The weights of both contributions are heavily dependent on the chosen value of τ. The simulation parameters are summarized in table S6.

The DQ-filtered PDHS spectrum shows a single splitting of ≈216 kHz corresponding to almost fourfold value of *T*. We found that simulation of the DQ-PDHS data requires a more specific dipolar kernel which accounts for the trigonal geometry of the CF_3_ group. The explicit consideration of the molecular geometry is essential as the blind spot functions are dependent on the hyperfine dipolar angles of all three fluorine atoms. If the correlation of the nuclear positions is neglected, the simulation notably fails (fig. S32). Eventually, the DQ-PDHS data were simulated with a three-spin dipolar kernel $K_{\text{DQ}}^{\left(3\right)}$ (see Eq. 35). The fitting results using a unimodal Gaussian distribution are shown in red in Fig. 9 and the used simulation parameters are summarized in table S6. We believe that further improvement between the experiment and simulation can be achieved with a better understanding of the background contribution in DQ-NEEM, as the latter affects the τ_RF_-dependence of DQ-PDHS. More details regarding the simulation are given in the experimental section. In these examples, we used a relatively large τ = 9 μs to observe the multispin effects and multiple-quantum contributions. Our simulations show that under such conditions, the geometry of the CF_3_ group is reflected in the PDHS traces. However, in routine applications, this may complicate the interpretation of PDHS data in terms of distance distribution.

In this work, we have presented TD Mims ENDOR spectroscopy for systems involving multiple ^19^F nuclei and developed a general theoretical framework that provides analytical solutions for arbitrary TD ENDOR with *N* coupled like nuclei. This framework provides a unified description of spin dynamics under the high-field approximation and describes the controlled generation and detection of single- and multiple-quantum coherences, offering deeper insights into spin dynamics in multispin systems. We demonstrated two complementary experiments that extend the capabilities of ^19^F-ENDOR spectroscopy for distance measurements. The NEEM experiment directly measures homonuclear dipolar couplings, allowing for distances and distance distributions between like nuclei to be determined by EPR. Complementary to this, PDHS provides information by selectively probing hyperfine couplings through single and MQ pathways. We showed that a 2D experiment can characterize the correlation between the orientations of hyperfine and nuclear coupling tensors, a finding that is valuable for structural studies. Extracting not only average distances but distance distributions is a clear advantage of both NEEM and PDHS, as these distributions can serve as powerful experimental constraints for in silico ensemble modeling (*64*–*66*).

The presence of sum-frequency modulations in multiple-quantum contributions could be potentially beneficial for detecting large electron-nucleus distances beyond the resolution limit of PDHS in mono-fluorinated systems. Moreover, MQ PDHS allows correlations between different hyperfine couplings to be established, analogous to multi-quantum experiments in NMR (*37*–*39*). However, the buildup of double and triple quantum signals requires long Mims preparation periods and correspondingly long electron coherence times, which may limit experimental applicability. Application to a CF_3_-labeled model compound highlights both the power and complexity of this approach. For distance measurements, we have shown that the complexity with the CF_3_ probe can be reduced. Only SQ contributions are detected when properly selecting the evolution time τ during the preparation period. From the analytical expression, we obtain the condition on τ such that $\text{sin}\left(\frac{A\tau }{2}\right)<\text{cos}\left(\frac{A\tau }{2}\right)$, i.e., $\tau <\frac{r^{3}}{4C}\approx 3.36\mathrm{\mu s}\cdot {\left(\frac{r}{\text{nm}}\right)}^{3}$, which effectively suppresses multispin effects. Under this condition, the additive approximation is valid, and the CF_3_ data can be analyzed analogously to mono-fluorinated compounds (fig. S33). Otherwise, for the CF_3_ probe, quantitative data processing requires an explicit evaluation of the multispin/multiple-quantum effects. Moreover, accurate data simulations in rigid molecules demand extended dipolar kernel functions that explicitly account for the geometry of the CF_3_ group.

Overall, our results present TD ENDOR as a versatile platform for probing hyperfine and homonuclear dipolar couplings in multinuclear systems. The combination of NEEM and PDHS provides structural information that was previously inaccessible with conventional FD ENDOR spectroscopy. These types of experiments could also be advantageous in the field of quantum-information science and quantum sensing, where the spin systems often have spectroscopic and relaxation properties favorable for multiple-quantum ENDOR (*58*, *59*, *67*, *68*). At the same time, the experiments highlight challenges such as the influence of molecular flexibility, the need for careful selection of experimental conditions and pulse sequence parameters, as well as the importance of explicitly treating multispin effects in data analysis. This positions TD ENDOR as a powerful tool for elucidating the structure of spin-labeled molecules, which will benefit a wide range of (bio-)chemical structural investigations.

## MATERIALS AND METHODS

### Experimental details

EPR and ENDOR measurements were performed at 10 to 50 K using a Bruker E580 X/Q-band spectrometer equipped with a Bruker EN 5107D2 pulse EPR/ENDOR resonator in a cryogen-free temperature cryostat for EPR (Cryogenic Limited, UK). A 170-W TWT amplifier (Model 187Ka, Applied Systems Engineering, USA) and a 600-W RF amplifier (600A225A Amplifier Research) were used to amplify MW and RF pulses, respectively. MW pulse lengths of 12 ns (π/2) and RF pulse lengths of 8 μs (π) were used, and the latter was controlled with RF nutation experiments. The RF pulse phase can be programmed to 0°, 90°, 180°, or 270°, allowing for single- and double-quantum filtering phase cycling. Single-quantum RF phase cycling protocol for NEEM and PDHS is +[0, 0, 0] − [180, 0, 0] + [0, 180, 0] − [180, 180, 0]. The eight-step PDHS phase cycling from (*31*) is obtained by the additional cycling of ϕ*_2_*, but was not used here. The double-quantum RF phase cycling protocol for NEEM and PDHS is +[0, 0, 0] − [90, 0, 0] + [180, 0, 0] − [270, 0, 0] + [0, 180, 0] − [90, 180, 0] + [180, 180, 0] − [270, 180, 0]. Alternation of ψ removes nuclear FID from the RF π pulse. Both given protocols are equivalent to detection of the real part (in-phase) of the nuclear echo. The imaginary part (quadrature) was detected by setting ϕ*_2_* to 90°.

Time delays τ, τ_RF_, and *t* were optimized for the respective experiments and are given in the figure captions. Shot repetition times (srt) were adjusted based on *T*_1,e_. The echo integration was performed in a 24 to 48 ns window placed symmetrically around the echo maximum with 10 (50 K) or fewer (20 K) shots per point (spp). Before Fourier transform, all time traces were background corrected, multiplied by a Hamming window and zero-filled to 10 times the length of the trace. In PDHS, only a constant offset was considered for background correction. In NEEM, a monoexponential background was subtracted from the signal (section S6).

### Sample preparation

Solid compounds **RF**_**2**_, **RFF′**, and **RCF**_**3**_ were dissolved in d_6_–dimethyl sulfoxide (DMSO):d_8_-glycerol (2:3 v/v) with concentrations ranging from 10 to 400 μM and then transferred to EPR tubes (outer diameter, 1.6 mm) with following shock-freezing in liquid nitrogen. DMSO (Eurisotop) and glycerol (Sigma-Aldrich) both had a deuteration degree of 99.8% according to the manufacturers.

### Simulation of NEEM in Fig. 4B

Simulation of orientation-selective SQ-NEEM data (**RF**_**2**_, Fig. 4B) was performed as follows. The direction of the external magnetic field **n** was uniformly sampled over a unit sphere generated with the sphgrid function in *EasySpin* (*69*). In the high-field approximation, vector **n** is a spin quantization axis. The secular hyperfine coupling constant with nucleus *j* and the secular dipolar coupling constant between nuclei *j* and *k* are calculated as

$$\begin{array}{c} A_{j}=-\frac{\mu_{0}}{4\text{πℏ}}\frac{g_{e}\mu_{B}g_{n}\mu_{N}}{r_{j}^{3}}\left(1-3{\text{cos}}^{2}\theta_{j}\right)\\ =\frac{2\pi C}{r_{j}^{3}}\left(3{\text{cos}}^{2}\theta_{j}-1\right)=2\pi T\left(3{\text{cos}}^{2}\theta_{j}-1\right) \end{array}$$ (24)

$$\omega_{j,k}=\frac{\mu_{0}}{4\text{πℏ}}\frac{g_{n}^{2}\mu_{N}^{2}}{r_{j,k}^{3}}\left(1-3{\text{cos}}^{2}\theta_{j,k}\right)$$ (25)

where μ_0_ is vacuum permeability, μ_B_ and μ_N_ are Bohr and nuclear magnetons, *g_e_* and *g_n_* are electron and nuclear *g* factors, and *C* = 74.5 MHz·Å^3^ is the dipolar constant for ^19^F. Dipolar angles were calculated as $\theta_{j}=\text{arccos}\frac{r_{j}\cdot n}{∥r_{j}∥}$, $\theta_{j,k}=\text{arccos}\frac{\left(r_{j}-r_{k}\right)\cdot n}{∥r_{j}-r_{k}∥}$. The hyperfine couplings were used to compute the blind spot functions from Eq. 21 for the SQ term. Note that these functions differ from the traditional Mims blind spot function. The NEEM modulation frequencies ( $\omega_{1,2}$ ) were weighted with the blind spot values at τ = 3 μs, and a histogram in the FD was formed. The Fourier transform of this histogram yielded the corresponding time traces. The simulation was repeated for both rotamers of **RF**_**2**_ and summed with equal weights.

The excitation bandwidth of the used MW pulses is narrower than the EPR spectrum. The line shape of the nitroxide spectrum is dominated by the *g* anisotropy of the electron spin and the anisotropic hyperfine interaction with the ^14^N nucleus of the nitroxide fragment. By selectively exciting parts of the EPR spectrum, we select fewer orientations on the sphere. To account for the orientation selection, we calculated electron spin resonance fields *B*_res_ for each vector **n**, and additionally weighted the NEEM frequencies in Eq. 25 with weights

$$w=\text{exp}\left(-\frac{{\left(B_{\text{res}}-B_{0}\right)}^{2}}{2s^{2}}\right)$$ (26)

approximating the excitation profile of the MW observer sequence with three π/2 pulses. Here, *B*_0_ is the experimental magnetic field, and parameter *s* = 0.6 mT was determined in a separate simulation (see section S5.1).

### Simulation of NEEM in Fig. 8A

The orientation-selective NEEM spectra of **RCF**_**3**_ in Fig. 8A were calculated similarly to those in Fig. 4B. The difference was that numeric propagation of spin density matrix was used instead of analytical expressions. This is explained in section S15.

### Kernel approach to fit distance distributions

NEEM trace in Fig. 7A and PDHS traces in Figs. 5C, 7A, and 9A were analyzed to extract the distance distributions assuming no orientation selection. Powder-averaged dipolar signal obtained in NEEM and PDHS can be described similarly to other dipolar EPR techniques like DEER (*30*)

$$V\left(t,\tau \right)=\int_{0}^{\infty }K\left(r,t,\tau \right)P\left(r\right)dr$$ (27)

Here, P(r) is the distribution of interspin distances and K(r,t,τ) is the dipolar kernel that describes the connection between the TD signal and the distance distribution. Strictly speaking, the signal for two and more nuclei is represented via a multivariate distribution $P\left(r_{1},r_{2},\ldots \right)$ (*70*). This also requires the introduction of multivariate kernels $K\left(r_{1},r_{2},\ldots ,t,\tau \right)$, which are memory-demanding due to the high sampling of the distance axes. However, for our systems with similar nitroxide-fluorine distances, we can use the effective univariate kernel K(r,t,τ) and distribution P(r). The same can be done if the additive approximation is valid and, in this case, P(r) can be approximated as the sum of marginal (projected) distributions for $P\left(r_{1},r_{2},\ldots \right)$.

For every fitted dataset, a distance vector *r* and a time vector *t* were defined to generate the appropriate kernel K(r,t,τ) (see summary below). Afterward, Eq. 28 was solved using either a mono- (**RF**_**2**_, **RCF**_**3**_) or a bi-Gaussian (**RFF′**) model for P(r).

$$G=\text{arg}\underset{P\geq 0}{\text{min}}\left\{{\left\|\int K\left(r,t,\tau \right)P\left(r\right)dr-V_{\text{Exp}}\left(t\right)\right\|}^{2}\right\}$$ (28)

where G is an array containing optimal model parameters and $V_{\text{Exp}}$ is the experimental data. In all simulations, the experimental values of τ were used.

### Dipolar kernels for specific experiments

NEEM data of **RFF′** (Fig. 7C) was processed with a simplified DEER-like dipolar kernel

$$K_{\text{NEEM}}\left(r,\tau_{\text{RF}}\right)=\int_{0}^{\frac{\pi }{2}}\text{sin}\text{θ cos}\left(\omega_{1,2}\tau_{\text{RF}}\right)d\theta$$ (29)

We note that this kernel neglects the τ dependence of the NEEM signal and assumes a pure dipolar Pake pattern for each distance *r*, which is an acceptable approximation for the case of **RFF′** where Θ angles are close to the right angle (see also section S5.2).

For PDHS with one fluorine, the dipolar kernel function was given as in (*31*)

$$K_{\text{SQ}}^{\left(1\right)}\left(r,t,\tau \right)=\int_{0}^{\frac{\pi }{2}}{\text{sin}}^{2}\frac{A\tau }{2}\cdot \text{sin}\text{θcos}\mathrm{At}\mathrm{d\theta}$$ (30)

Here, τ is the separation between the two MW preparation pulses in the pulse sequence (see Fig. 1C), *A* is the hyperfine coupling that depends on both *r* and θ (Eq. 24), and the factor sinθ accounts for the statistical weight. Here and in the following, we label kernels with a superscript corresponding to the number of fluorines and with a subscript corresponding to the coherence order. On the basis of the analytical equations of PDHS for two (Eq. 21) and three (Eq. 23) fluorine atoms, we can formulate the following kernel functions for PDHS. In a simple model, a small cluster of fluorines can be approximated as a point object, i.e., *A*_1_ ≈ *A*_2_. Thus, for PDHS with two fluorine atoms acquired with a single-quantum filter, we obtain

$$K_{\text{SQ}}^{\left(2\right)}\left(r,t,\tau \right)=\int_{0}^{\frac{\pi }{2}}{\text{sin}}^{2}\frac{A\tau }{2}{\text{cos}}^{2}\frac{A\tau }{2}\cdot \text{sin}\text{θcos}\mathrm{At}\mathrm{d\theta}$$ (31)

Similarly, for the DQ-filtered PDHS with two fluorines, we used

$$K_{\text{DQ}}^{\left(2\right)}\left(r,t,\tau \right)=\int_{0}^{\frac{\pi }{2}}{\text{sin}}^{4}\frac{A\tau }{2}\cdot \text{sin}\text{θcos}2\mathrm{At}\mathrm{d\theta}$$ (32)

In **RCF**_**3**_, at low τ values, PDHS measurements can be processed in the additive approximation (fig. S31). Oppositely, at large τ values, the multispin effects cannot be ignored. Furthermore, we found that the point-object approximation *A_1_ ≈ A_2_ ≈ A_3_* is not optimal in this case (fig. S32) because the hyperfine dipolar angles of the three fluorines are slightly different, even if their distances to the nitroxide are the same. In other words, the PDHS spectra depend on both nitroxide-CF_3_ distance and orientation of the CF_3_ group. Overall, for τ = 9 μs, we switched to univariate models, where the kernels incorporate the geometry of the CF_3_ group

$$K_{\text{SQ},\text{SO}=1}^{\left(3\right)}\left(r,t,\tau \right)=\sum_{i=1}^{3}\int {\text{sin}}^{2}\frac{A_{i}\tau }{2}{\text{cos}}^{2}\frac{A_{j}\tau }{2}{\text{cos}}^{2}\frac{A_{k}\tau }{2}\cdot \text{cos}A_{i}t\mathrm{d\Omega}$$ (33)

$$\begin{array}{c} K_{\text{SQ},\text{SO}=3}^{\left(3\right)}\left(r,t,\tau \right)=\int \left(\prod_{i=1}^{3}{\text{sin}}^{2}\frac{A_{i}\tau }{2}\right)\\ \left[\text{cos}\left(-A_{1}+A_{2}+A_{3}\right)t+\text{cos}\left(A_{1}-A_{2}+A_{3}\right)t+\text{cos}\left(A_{1}+A_{2}-A_{3}\right)t\right]\mathrm{d\Omega} \end{array}$$ (34)

$$K_{\text{DQ}}^{\left(3\right)}\left(r,t,\tau \right)=\sum_{\begin{array}{c} i,j=1\\ i<j \end{array}}^{3}\int {\text{sin}}^{2}\frac{A_{i}\tau }{2}{\text{sin}}^{2}\frac{A_{j}\tau }{2}{\text{cos}}^{2}\frac{A_{k}\tau }{2}\cdot \text{cos}\left(A_{i}+A_{j}\right)t\mathrm{d\Omega}$$ (35)

$$K_{\text{TQ}}^{\left(3\right)}\left(r,t,\tau \right)=\int \left(\prod_{i=1}^{3}{\text{sin}}^{2}\frac{A_{i}\tau }{2}\right)\text{cos}\left(A_{1}+A_{2}+A_{3}\right)t\mathrm{d\Omega}$$ (36)

where i≠j≠k, dΩ=sinθdθdϕ with ϕ being the internal rotation angle of the CF_3_ group, and SO stands for spin order. Since our current experimental setup is limited in terms of the available RF phases, our single-quantum filter on compound **RCF**_**3**_ (see the “Experimental details” section) also acquires the TQ contribution. To simulate the data, we used the following combined kernel

$$K_{\text{SQ}}^{\left(3\right)}\left(r,t,\tau \right)=f\cdot K_{\text{SQ},\text{SO}=1}^{\left(3\right)}\left(r,t,\tau \right)+K_{\text{SQ},\text{SO}=3}^{\left(3\right)}\left(r,t,\tau \right)+K_{\text{TQ}}^{\left(3\right)}\left(r,t,\tau \right)$$ (37)

where *f* accounts for the τ_RF_-scaling due to factors $\text{cos}\left(\omega_{1,2}\tau_{\text{RF}}\right)$, $\text{cos}\left(\omega_{1,3}\tau_{\text{RF}}\right)$, etc. (see lines 2 to 4 in Eq. 23). We used it as an adjustable parameter.

## Supplementary Materials

### This PDF file includes:

Supplementary Text Figs. S1 to S33 Tables S1 to S6 References
