# Supplementary material for: Multiple quantum 19F electron-nuclear double resonance (ENDOR) for distance measurements with multifluorinated spin probes
Source: Sci Adv. 2026 Sep 16;12(38):eaeg7753. doi: 10.1126/sciadv.aeg7753 (PMC13580543; doi:10.1126/sciadv.aeg7753)
Supplement: Supplementary file 1 [file sciadv.aeg7753_sm.pdf]

Science Advances
AAAS

Supplementary Materials for

Multiple quantum ^{19}F electron-nuclear double resonance (ENDOR) for distance measurements with multifluorinated spin probes

Sergei Kuzin *et al.*

Corresponding author: Sergei Kuzin, sergei.kuzin@mpinat.mpg.de;
Marina Bennati, marina.bennati@mpinat.mpg.de

Sci. Adv. **12**, eaeg7753 (2026)
DOI: 10.1126/sciadv.aeg7753

This PDF file includes:

Supplementary Text
Figs. S1 to S33
Tables S1 to S6
References

S1 Details on formalism of multi-spin ENDOR experiments

S1.1 Spin Hamiltonian

We consider a spin Hamiltonian of one electron spin and N nuclei of the same kind (e.g., ^{19}F), i.e., a homonuclear case. To obtain analytical expressions for ENDOR signals, we assume a high-field approximation (HFA) for all spins and truncate the spin interactions accordingly:

$$\hat{H} = \hat{H}_{\mathrm{EZ}} + \hat{H}_{\mathrm{hf}} + \hat{H}_{\mathrm{CS}} + \hat{H}_{\mathrm{nd}} = \Omega \hat{S}_z + \sum_{j=1}^{N} A_j \hat{S}_z \hat{I}_{j,z} + \sum_{j=1}^{N} \Delta\omega_j \hat{I}_{j,z} + \sum_{j<k} \omega_{j,k} \hat{I}_{j,z} \hat{I}_{k,z} \,. \tag{S1}$$

Due to this truncation, this Hamiltonian is diagonal in the Zeeman product basis of $(N+1)$ spins. It is further convenient to arrange the coupling constants into vectors and matrices as follows:

$$\boldsymbol{\Delta\omega} = \begin{pmatrix} \Delta\omega_1 \\ \Delta\omega_2 \\ \vdots \\ \Delta\omega_N \end{pmatrix} \qquad \mathbf{A} = \begin{pmatrix} A_1 \\ A_2 \\ \vdots \\ A_N \end{pmatrix} \qquad \boldsymbol{\omega} = \begin{pmatrix} \boldsymbol{\omega}_1 & \boldsymbol{\omega}_2 & \cdots & \boldsymbol{\omega}_N \end{pmatrix} = \begin{pmatrix} 0 & \omega_{1,2} & \cdots & \omega_{1,N} \\ \omega_{2,1} & 0 & \cdots & \omega_{2,N} \\ \vdots & \vdots & \ddots & \vdots \\ \omega_{N,1} & \omega_{N,2} & \cdots & 0 \end{pmatrix} \tag{S2}$$

The matrix of nuclear-nuclear couplings $\boldsymbol{\omega}$ is symmetric, has zeroes on the main diagonal, and its columns will be referred to later as vectors $\boldsymbol{\omega}_i$.

We also consider ideal microwave (MW) and radio-frequency (RF) pulses with an arbitrary phase ϕ in the x-y plane ($\phi = 0$ stands for "$+x$" pulse, etc.), whose Hamiltonians in rotating frames are

$$\hat{H}_{\mathrm{MW}} = \beta \hat{S}_{\phi} \qquad\qquad \hat{H}_{\mathrm{RF}} = \beta \sum_{j=1}^{N} \hat{I}_{j,\phi} \tag{S3}$$

where β is the flip angle and $\hat{S}_{\phi} = \hat{S}_x \cos\phi + \hat{S}_y \sin\phi$ (and similarly for $\hat{I}_{j,\phi}$). Note that we use high-power broadband RF pulses, which are, hence, non-selective and act on all nuclei. The sum of operators on the right is often shortened to $\hat{F}_{\phi}$.

S1.1.1 Heteronuclear dipolar coupling

We can consider a more general spin Hamiltonian than in Eq. (S1), by adding K nuclei of spin 1/2 that are not manipulated by the RF pulses. For the ^{19}F ENDOR spectroscopy, this covers the presence of protons near fluorines. The augmented Hamiltonian is

$$\hat{H}_{\mathrm{gen}} = \hat{H} + \sum_{\lambda=1}^{K} \omega_n \hat{I}_{\lambda,z} + \sum_{\lambda=1}^{K} A_{\lambda} \hat{S}_z \hat{I}_{\lambda,z} + \sum_{j,\lambda=1}^{N,K} \omega_{j,\lambda} \hat{I}_{j,z} \hat{I}_{\lambda,z} \tag{S4}$$

where we use the Latin index for RF-manipulated nuclei and the Greek index for bystander nuclei. Suppose HFA applies to the added nuclei, i.e., no interaction may affect their nuclear state during the ENDOR experiment, e.g., pseudo-secular hyperfine coupling, homonuclear flip-flop terms, methyl rotor tunneling, etc. In that case, their nuclear states are constants of motion, and we may replace the operators $\hat{I}_{\lambda,z}$ by the corresponding magnetic quantum numbers $m_I(\lambda)$. After this, we deal with 2^K reduced spin Hamiltonians as in Eq. (S1), where the resonance offset of each nucleus j is added with the effective dipolar field: $\Delta\omega_j \to \Delta\omega_j + \sum_{\lambda=1}^{K} \omega_{j,\lambda} m_I(\lambda)$. In other words, the nuclear resonance offsets are distributed in this model. This idea can be extended to higher nuclear spins, e.g., solvent ^{2}H in this work, where the quadrupolar coupling can be neglected or treated in the HFA. This generalization impacts the time-domain (TD) ENDOR experiments with nuclear free induction decay (FID) detection, such as dead-time-free (DTF)- TD ENDOR (*29*). However, in this work, we discuss only experiments based on nuclear spin echoes, where the chemical shift and resonance offsets are refocused. Therefore, we do not address the role of other magnetic nuclei in ^{19}F ENDOR experiments.

S1.2 Spherical basis for density matrix

Evolution of a spin density matrix $\hat{\sigma}$ under a time-independent Hamiltonian $\hat{H}$ can be computed via the Baker-Campbell-Hausdorff formula

$$U(t)\,\hat{\sigma}\,U(t)^{\dagger} = \hat{\sigma} + (-it)[\hat{H},\hat{\sigma}] + \frac{(-it)^2}{2!}[\hat{H},[\hat{H},\hat{\sigma}]] + ... \tag{S5}$$

where $U(t) = \exp(-i\hat{H}t)$ is a propagator.

In the analysis of multiple-spin systems in the high-field approximation, writing the density matrix in a spherical basis (*45*) $\hat{S}_z$, $\hat{S}^+ = \hat{S}_x + i\hat{S}_y$ and $\hat{S}^- = \hat{S}_x - i\hat{S}_y$ is particularly advantageous because this basis consists of the eigenoperators for the evolution under $\hat{S}_z$:

$$[\hat{S}_z,\hat{S}^+] = \hat{S}^+ \qquad [\hat{S}_z,\hat{S}^-] = -\hat{S}^- \qquad [\hat{S}_z,\hat{S}_z] = 0\,. \tag{S6}$$

$\hat{S}^+$ and $\hat{S}^-$ represent coherences, and $\hat{S}_z$ corresponds to the polarization. The main property of the raising and lowering operators, following from Eq. (S6), is how they are rotated about the z axis:

$$\exp(-i\theta\hat{S}_z)\hat{S}^+\exp(i\theta\hat{S}_z) = e^{-i\theta}\hat{S}^+ \qquad \exp(-i\theta\hat{S}_z)\hat{S}^-\exp(i\theta\hat{S}_z) = e^{+i\theta}\hat{S}^-\,. \tag{S7}$$

Upon rotation, they simply obtain phase factors. As a result, the analytical form of the density matrix is substantially shorter when written in terms of raising and lowering operators. Next, since $\hat{S}^-$ is adjoint to $\hat{S}^-$, it is sufficient to consider the evolution of one of them. The price of such a simplification is that the solution is no longer a Hermitian matrix/operator; hence, it must be separated before detection. The Hermitian part of the solution corresponds to the evolution of $\hat{S}_x$ while the anti-Hermitian part is the evolution of $\hat{S}_y$ as demonstrated in Figure S1.

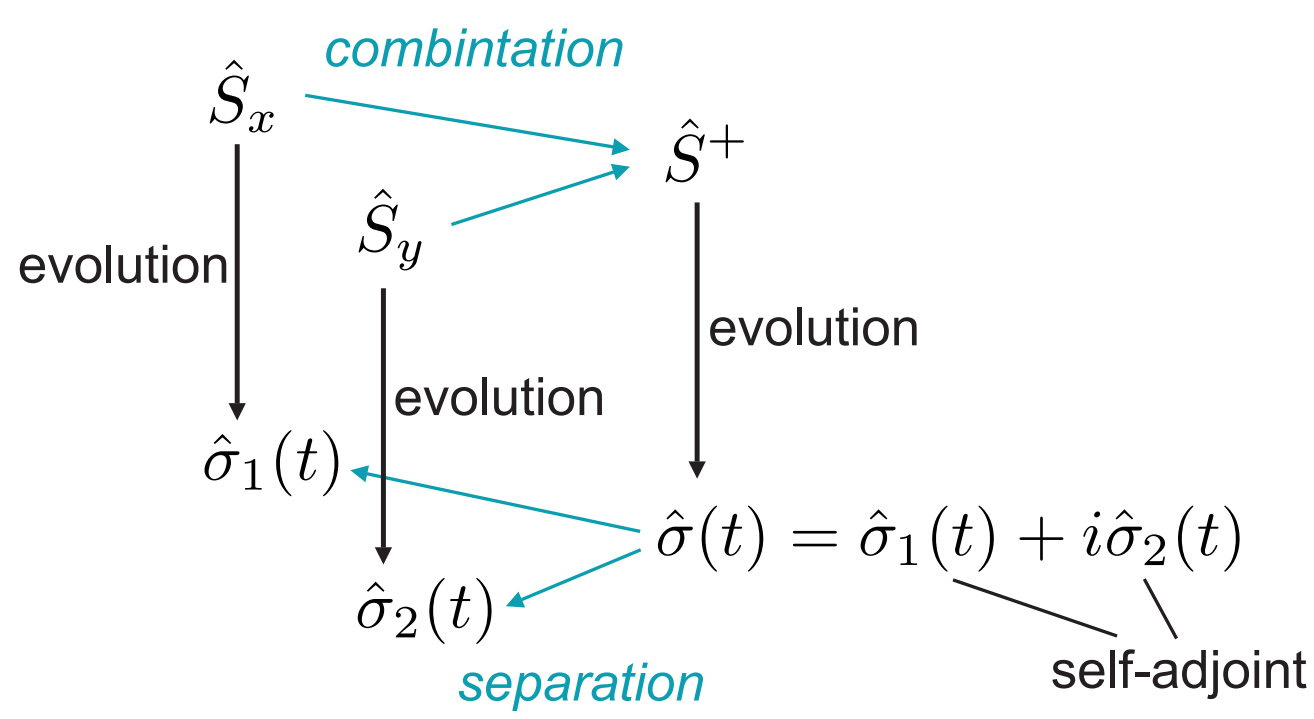

Figure S1: **Connection between evolution of operators $\hat{S}_x$, $\hat{S}_y$ and $\hat{S}^+$ in density matrix.** $\hat{S}_x$ and $\hat{S}_y$ can be combined in non-Hermitian $\hat{S}^+$, whose evolution result, $\hat{\sigma}(t)$, is also non-Hermitian and can be decomposed as $\hat{\sigma}_1(t) + i\hat{\sigma}_2(t)$. The two terms automatically correspond to the evolution of $\hat{S}_x$ and $\hat{S}_y$, respectively.

Spherical notation can be 'visualized' by noticing

$$\hat{S}_\phi = \hat{S}_x\cos\phi + \hat{S}_y\sin\phi = \frac{1}{2}\left(e^{-i\phi}\hat{S}^+ + e^{i\phi}\hat{S}^-\right)\,. \tag{S8}$$

The left-hand side (LHS) represents the magnetization vector in the x-y plane rotated by angle ϕ from the $+x$ direction. The two terms on the right-hand side are Hermitian conjugates of one another, and each of them can be associated with the LHS.

The application of a π pulse with a phase ϕ in the x-y plane is calculated as follows:

$$\hat{S}^+ \xrightarrow{\pi\hat{S}_\phi} e^{2i\phi}\hat{S}^- \text{ and } \hat{S}^- \xrightarrow{\pi\hat{S}_\phi} e^{-2i\phi}\hat{S}^+\,, \tag{S9}$$

where these expressions can be derived via an equivalent chain, (*71*) e.g.

$$\hat{S}^+ \xrightarrow{-\phi\hat{S}_z} e^{i\phi}\hat{S}^+ \xrightarrow{\pi\hat{S}_x} e^{i\phi}\hat{S}^- \xrightarrow{\phi\hat{S}_z} e^{2i\phi}\hat{S}^-\,. \tag{S10}$$

The first and the third arrows are treated as in Eq. (S7), while the middle arrow can be confirmed via the definition of $\hat{S}^+$ and $\hat{S}^-$. Finally, $\pi/2$ pulses act as follows:

$$\hat{S}^{\pm} \xrightarrow{\frac{\pi}{2}\hat{S}_\phi} e^{\pm i(\phi+\pi/2)}\hat{S}_z + \frac{1}{2}\left(\hat{S}^{\pm} + e^{\pm i2\phi}\hat{S}^{\mp}\right). \tag{S11}$$

S1.3 Evolution of $\hat{S}^+$ and $\hat{S}^-$

Now, we consider the time evolution of a density matrix written in the spherical basis. We can show via direct computation:

$$\begin{aligned}\hat{S}^+ \xrightarrow{\Omega t\hat{S}_z} &\cos\Omega t\hat{S}_x + \sin\Omega t\hat{S}_y + i(\cos\Omega t\hat{S}_y - \sin\Omega t\hat{S}_x) \\ &= \cos\Omega t(\hat{S}_x + i\hat{S}_y) - i\sin\Omega t(\hat{S}_x + i\hat{S}_y) \\ &= e^{-i\Omega t}\hat{S}^+\end{aligned} \tag{S12}$$

fully consistent with Eq. (S7). This result says that in the free evolution, $\hat{S}^+$ obtains a t-dependent phase $-\Omega t$. This can be understood as $\hat{S}^+$ corresponds to a coherence element of the density matrix. It can be written as $\hat{S}^+ = |\alpha\rangle\langle\beta|$, therefore

$$\hat{S}^+ \xrightarrow{\Omega t\hat{S}_z} U(t)|\alpha\rangle\langle\beta|U^\dagger(t) = e^{-i\Omega t/2}|\alpha\rangle\langle\beta|e^{-i\Omega t/2} = e^{-i\Omega t}|\alpha\rangle\langle\beta| = e^{-i\Omega t}\hat{S}^+ . \tag{S13}$$

Note again the negative sign of Ωt. The free evolution of $\hat{S}^-$ yields $e^{i\Omega t}\hat{S}^-$.

If the Hamiltonian includes nuclear operators, e.g. hyperfine interaction $\hat{H} = \hat{S}_z \sum_i A_i \hat{I}_{iz}$, we consider the commutator

$$[\hat{S}_z\hat{h}, \hat{S}^+] = \hat{S}^+\hat{h} \tag{S14}$$

where $\hat{h} = \sum_i A_i \hat{I}_{iz}$. This equation holds for any form of $\hat{h}$ as long as it commutes with $\hat{S}_z$ and $\hat{S}^+$.

A repeated commutator yields

$$[\hat{S}_z\hat{h}, [\hat{S}_z\hat{h}, \hat{S}^+]] = [\hat{S}_z\hat{h}, \hat{S}^+\hat{h}] = \hat{S}^+\hat{h}^2 . \tag{S15}$$

The generalisation for the higher numbers of nested commutators is evident at this point. After substitution into Eq. (S5), we obtain

$$\hat{S}^+\left(\hat{1} + (-it)\hat{h} + \frac{(-it)^2}{2!}\hat{h}^2 + ...\right) \tag{S16}$$

where we recognise the expression for the operator exponential, hence

$$\hat{S}^+ \xrightarrow{\hat{S}_z\hat{h}t} \hat{S}^+ e^{-it\hat{h}} . \tag{S17}$$

The result in Eq. (S12) is the special case for $\hat{h} = \Omega$. We would like to emphasize that the results obtained so far are based on Eq. (S5). Since the commutation relations between spin operators are the same for any spin, the results also apply to any spin.

S1.4 Example: Hahn echo

Here, we briefly show an advantage of the calculation with raising/lowering operators if the Hamiltonian is taken in the high-field approximation. For this, we compute the Hahn echo sequence $(\pi/2)_{-y} - t - \pi_{+x} - t$ for a two-spin system with $\hat{H} = \Omega\hat{S}_z + A\hat{S}_z\hat{I}_z$ (i.e. resonance offset and one coupled nucleus). The phase of the first pulse was chosen such that it converts the equilibrium density matrix $-\hat{S}_z$ into $+\hat{S}_x = (\hat{S}^+ + \hat{S}^-)/2$. It is enough to follow the evolution of $\hat{S}^+$ because of the symmetry between $\hat{S}^+$ and $\hat{S}^-$. We proceed as

$$\begin{aligned}\hat{S}^+ &\xrightarrow{\hat{H}t} e^{-iAt\hat{I}_z}e^{-i\Omega t}\hat{S}^+ \\ &\xrightarrow{\pi_{+x}} e^{-iAt\hat{I}_z}e^{-i\Omega t}\hat{S}^- \\ &\xrightarrow{\hat{H}t} e^{iAt\hat{I}_z}e^{i\Omega t}e^{-iAt\hat{I}_z}e^{-i\Omega t}\hat{S}^- = \hat{S}^- .\end{aligned} \tag{S18}$$

We emphasise that the simplification in the last line is legitimate since these exponentials commute. Hence, we obtained overall $\hat{S}^+ \to \hat{S}^-$. By taking the Hermitian part of the result (Figure S1): $\hat{S}_x \to \hat{S}_x$, also the anti-Hermitian part: $\hat{S}_y \to -\hat{S}_y$, which can be confirmed by standard product operator calculations. This illustrates a well-known result: the secular hyperfine coupling is refocused in a spin-echo experiment. Remarkably, in this exercise, the conclusion and the size of the calculation effort are independent of the number of coupled nuclei. The latter, however, grows prominently for the product operator computation in a Cartesian basis.

S1.5 Mims preparation block (three-spin case and general expression)

Similar to the previous Section, we start with $(\pi/2)_{-y}$ pulse that converts $-\hat{S}_z \to \hat{S}_x$. The propagation of $\hat{S}^+$ in the Mims preparation block $\pi/2 - \tau$ repeats the first line in Eq. (S18). We exemplify it for two coupled nuclei

$$\hat{S}^+ \xrightarrow{\hat{H}\tau} e^{-iA_2\tau\hat{I}_{2,z}} e^{-iA_1\tau\hat{I}_{1,z}} e^{-i\Omega t}\hat{S}^+ \,. \tag{S19}$$

Note that nuclear-nuclear coupling does not affect the evolution of the state here.

We group the nuclear terms in an auxiliary nuclear operator $\hat{M}^+ = e^{-iA_2\tau\hat{I}_{2,z}} e^{-iA_1\tau\hat{I}_{1,z}}$. It is a sum of the following four terms

$$\hat{M}^+ = \begin{cases} \cos\dfrac{A_1\tau}{2}\cos\dfrac{A_2\tau}{2}\hat{1} \\ \left(-2i\sin\dfrac{A_1\tau}{2}\right)\cos\dfrac{A_2\tau}{2}\hat{I}_{1,z} \\ \cos\dfrac{A_1\tau}{2}\left(-2i\sin\dfrac{A_2\tau}{2}\right)\hat{I}_{2,z} \\ \left(-2i\sin\dfrac{A_1\tau}{2}\right)\left(-2i\sin\dfrac{A_2\tau}{2}\right)\hat{I}_{1,z}\hat{I}_{2,z} \end{cases} \tag{S20}$$

To arrive at this linear form, we used the following identity for spin 1/2

$$e^{-ia\hat{I}_z} = \cos\frac{a}{2}\hat{1} - 2i\sin\frac{a}{2}\hat{I}_z \,. \tag{S21}$$

Next, we group the powers of 2 with the spin operators to normalise them. Operator $\hat{M}^+$ is non-Hermitian, which is natural since $\hat{S}^+$ is non-Hermitian itself. It can be represented as $\hat{M}^+ = \hat{M}_{\mathrm{Re}} + i\hat{M}_{\mathrm{Im}}$ where the Hermitian part is

$$\hat{M}_{\mathrm{Re}} = \begin{cases} \cos\dfrac{A_1\tau}{2}\cos\dfrac{A_2\tau}{2}\hat{1} \\ -\sin\dfrac{A_1\tau}{2}\sin\dfrac{A_2\tau}{2}4\hat{I}_{1,z}\hat{I}_{2,z} \end{cases} \tag{S22}$$

and the anti-Hermitian part (without i) is

$$\hat{M}_{\mathrm{Im}} = \begin{cases} -\sin\dfrac{A_1\tau}{2}\cos\dfrac{A_2\tau}{2}2\hat{I}_{1,z} \\ -\cos\dfrac{A_1\tau}{2}\sin\dfrac{A_2\tau}{2}2\hat{I}_{2,z} \end{cases} \,. \tag{S23}$$

The numeric coefficients are called *generation amplitudes/coefficients*, c_g, in this work. They depend on τ and are different for different nuclear operators. Very conveniently, they can be calculated at once for any number of nuclei. Let $\overline{1,N}$ be a set of integers from 1 to N (N is the number of coupled nuclei) and $X \subseteq \overline{1,N}$ its subset. Then let's define

$$\hat{I}_{X,z} = \prod_{j\in X}\hat{I}_{j,z} \,, \tag{S24}$$

i.e. a product of several z-operators indexed by X. As can be seen in Eqs. (S22) and (S23), such products appear in the density matrix for every subset of nuclear indices X (4 possibilities $X = \{\}, \{1\}, \{2\}, \{1,2\}$ for two nuclei,

8 for three, etc.). The generation coefficient for any product $\hat{I}_{X,z}$ is given by

$$c_g(X,\tau) = (-1)^{\lceil \frac{|X|}{2} \rceil} \left(\prod_{j\in X} \sin\frac{A_j\tau}{2} \right) \left(\prod_{k\notin X} \cos\frac{A_k\tau}{2} \right) . \tag{S25}$$

This expression is always composed of N factors, whereby every z-operator present in $\hat{I}_{X,z}$ brings a sin-factor, and every other index enters with a cos-factor.

After application of the second MW $(\pi/2)_{-y}$ pulse, which converts $\hat{S}^+ \to \hat{S}_z$ (remaining coherences are neglected), we take the Hermitian part of the total density matrix according to the rules in Section S1.2. The result is

$$\hat{\sigma}(\tau) = \cos\Omega\tau\hat{S}_z\hat{M}_{\mathrm{Re}} + \sin\Omega\tau\hat{S}_z\hat{M}_{\mathrm{Im}} \,, \tag{S26}$$

where in the general case

$$\hat{M}_{\mathrm{Re}}(\tau) = \sum_{\substack{X\subseteq\overline{1,N}\\ |X| \text{ is even}}} c_g(X,\tau)2^{|X|}\hat{I}_{X,z} \qquad\qquad \hat{M}_{\mathrm{Im}}(\tau) = \sum_{\substack{X\subseteq\overline{1,N}\\ |X| \text{ is odd}}} c_g(X,\tau)2^{|X|}\hat{I}_{X,z} \,. \tag{S27}$$

The coefficients $c_g(X,\tau)$ are equal to the weights with which the given normalized spin order $2^{|X|}\hat{S}_z\hat{I}_{X,z}$ is generated. Consequently, they generalise the idea of ENDOR blindspots to the multiple-nuclear case.

Due to the distribution of the electron spin resonance offset Ω, both terms contribute to the target spin echo independently of the phase of the second MW $\pi/2$ pulse (see Section S1.6). Therefore, one can treat the Mims preparation block as a converter of $\hat{S}_z$ into products $\hat{S}_z\hat{I}_{X,z}$ with all possible nuclear subsets $X \subseteq \{1,...,N\}$.

S1.6 Distribution of resonance offsets

Before further developing the formalism for ENDOR experiments, we consider the solution's dependence on the resonance offset (Ω). We can show that it can be integrated over Ω implicitly, at the cost of correcting the detection operator.

After spin manipulation during the mixing block, before the Mims block reconversion, the density matrix can be written as

$$\hat{\sigma}' = \cos\Omega\tau\hat{S}_z\hat{M}'_{\mathrm{Re}} + \sin\Omega\tau\hat{S}_z\hat{M}'_{\mathrm{Im}} \tag{S28}$$

where $\hat{M}'_{\mathrm{Re}}$ and $\hat{M}'_{\mathrm{Im}}$ are new nuclear operators, and where we assumed that the mixing does not contain periods of electron spin transverse evolution. Note that at this stage, we have neglected terms that are not proportional to $\hat{S}_z$ as they are not detectable in our experiments.

Instead of propagating $\hat{\sigma}'$ forward to the echo detection event, we propagate the detection operator back in time to the point of $\hat{\sigma}'$. Choosing the detection operator $\hat{\sigma}_{\mathrm{det}} = \hat{S}_x$, its back-propagated form is simply $\hat{\sigma}'_{\mathrm{det}} = \hat{\sigma}(-\tau)$ (see Eq. (S26)). Hence, we write the electron spin echo intensity

$$\begin{aligned} V_\Omega = \mathrm{tr}(\hat{\sigma}'\hat{\sigma}'_{\mathrm{det}}) = \mathrm{tr}(\hat{\sigma}'\hat{\sigma}(-\tau)) &= \cos^2\Omega\tau\cdot\mathrm{tr}(\hat{M}'_{\mathrm{Re}}\hat{M}_{\mathrm{Re}}(-\tau)) \\ &- \sin^2\Omega\tau\cdot\mathrm{tr}(\hat{M}'_{\mathrm{Im}}\hat{M}_{\mathrm{Im}}(-\tau)) \\ &+ \sin\Omega\tau\cos\Omega\tau\cdot\mathrm{tr}(\hat{M}'_{\mathrm{Im}}\hat{M}_{\mathrm{Re}}(-\tau)) \\ &- \sin\Omega\tau\cos\Omega\tau\cdot\mathrm{tr}(\hat{M}'_{\mathrm{Re}}\hat{M}_{\mathrm{Im}}(-\tau)) \,. \end{aligned} \tag{S29}$$

We note that this expression, for a single nucleus, is equivalent to the well-known expression for frequency-domain ENDOR with ideal selective RF pulses (see Eq. (39) in Ref. (*33*)). This can be confirmed after substitution: $\hat{M}_{\mathrm{Re}}(\tau) = \hat{M}'_{\mathrm{Re}}(\tau) = \cos(A\tau/2)\hat{1}$, $\hat{M}_{\mathrm{Im}}(\tau) = -\sin(A\tau/2)2\hat{I}_z$, and, for off-resonant RF, $\hat{M}'_{\mathrm{Im}}(\tau) = \hat{M}_{\mathrm{Im}}(\tau)$ and, for on-resonant RF, $\hat{M}'_{\mathrm{Im}}(\tau) = 0$. The latter is because the selective RF pulse performs $2\hat{S}_z\hat{I}_z \to \pm\hat{I}_z$, where the sign depends on the electron spin manifold.

The total signal is the weighted average

$$V = \int f(\Omega)f_p(\Omega)V_\Omega \;\mathrm{d}\Omega \tag{S30}$$

where $f(\Omega)$ is the EPR spectral shape and $f_p(\Omega)$ is the excitation profile of the microwave pulse subsequence. If we approximate both functions as symmetric, the last two terms in Eq. (S29) average to zero. Furthermore, if we recall $\cos^2(x) = (1+\cos 2x)/2$ and $\sin^2(x) = (1-\cos 2x)/2$, Eq. (S30) transforms to

$$V = \frac{1}{2}\int f(\Omega) f_p(\Omega) \left[\mathrm{tr}(\hat{M}'_{\mathrm{Re}}\hat{M}_{\mathrm{Re}}(-\tau)) - \mathrm{tr}(\hat{M}'_{\mathrm{Im}}\hat{M}_{\mathrm{Im}}(-\tau))\right] \mathrm{d}\Omega$$
$$+ \frac{1}{2}\int f(\Omega) f_p(\Omega) \cos 2\Omega\tau \left[\mathrm{tr}(\hat{M}'_{\mathrm{Re}}\hat{M}_{\mathrm{Re}}(-\tau)) + \mathrm{tr}(\hat{M}'_{\mathrm{Im}}\hat{M}_{\mathrm{Im}}(-\tau))\right] \mathrm{d}\Omega\,. \quad \text{(S31)}$$

The first integral repeats Eq. (S30) with a simplified expression for V_Ω. The second integral is typically neglected, but this is not obvious; we should discuss it. The expression in brackets may depend on Ω since the coupling constants A_j and $\omega_{j,k}$ may depend on the resonant frequency of different spin packets – a phenomenon leading to an orientation selection in EPR. This can be expected for rigid nitroxide radicals where the eigenframes of the coupling tensors are correlated with the eigenframes of the g- and $A(^{14}\mathrm{N})$-tensors. To estimate the second integral, let's split it into smaller domains of size $2\pi\Delta$ in which the coupling parameters are constant. How large should Δ be for the second integral to vanish? The smaller integral is simply a Fourier integral of boxcar shape (i.e., sinc function) evaluated at the point 2τ. This gives the value not larger than $(4\pi\tau\Delta)^{-1}$. Allowing that value of 5% is negligible, the Δ should be at least $20/(4\pi\tau)$. Already at $\tau = 1$ µs, $\Delta \approx 1.5$ MHz or 0.5 G. For the conditions of this work, i.e. nitroxide radicals at Q band (spectral width ≈ 100 G, see e.g. Figure S11), this is an acceptable approximation, and the second integral indeed vanishes. This result is consistent with ENDOR literature, where terms $\cos^2\Omega\tau$ and $\sin^2\Omega\tau$ are directly averaged to 1/2. Actually, Δ is currently overestimated because the signals from different sub-domains destructively interfere.

After this consideration, the target signal from a fixed spin packet (see the first integral) is expressed as

$$V_\Omega \propto \mathrm{tr}(\hat{M}_{\mathrm{Re}}\hat{M}'_{\mathrm{Re}}) + \mathrm{tr}(\hat{M}_{\mathrm{Im}}\hat{M}'_{\mathrm{Im}}) \quad \text{(S32)}$$

where we simplified $\hat{M}_{\mathrm{Re}}(-\tau) = \hat{M}_{\mathrm{Re}}(\tau)$ and $\hat{M}_{\mathrm{Im}}(-\tau) = -\hat{M}_{\mathrm{Im}}(\tau)$. Without consideration of the resonance offset, which is equivalent to letting $\Omega = 0$ in Eq. (S26), only the first term in this sum would be obtained. In further simulations, we compute signals V_Ω for each spin packet and sum them with weights as in Eq. (S30).

S1.7 Mixing in time-domain ENDOR

For the propagation during the mixing block, we represent the coherences generated by the RF $\pi/2$ pulse via raising and lowering operators. To this end, we note

$$2\hat{S}_z\hat{I}_{1,z} \xrightarrow{\frac{\pi}{2}\hat{I}_{1,\phi}} 2\hat{S}_z\left(\hat{I}_x\cos(\phi-\pi/2) + \hat{I}_y\sin(\phi-\pi/2)\right)$$
$$= \hat{S}_z\left(\hat{I}_1^+ e^{-i(\phi-\pi/2)} + \hat{I}_1^- e^{i(\phi-\pi/2)}\right)\,. \quad \text{(S33)}$$

The transformation in the second line was introduced in Eq. (S8). Spin nutation in the multinuclear product operator happens simultaneously for all factors; e.g.

$$4\hat{S}_z\hat{I}_{1,z}\hat{I}_{2,z} \xrightarrow{\frac{\pi}{2}(\hat{I}_{1,\phi}+\hat{I}_{2,\phi})} \hat{S}_z\left(\hat{I}_1^+ e^{-i(\phi-\pi/2)} + \hat{I}_1^- e^{i(\phi-\pi/2)}\right)\left(\hat{I}_2^+ e^{-i(\phi-\pi/2)} + \hat{I}_2^- e^{i(\phi-\pi/2)}\right)\,. \quad \text{(S34)}$$

If $\phi = 0$ or π then the right-hand side is equal to $4\hat{S}_z\hat{I}_{1,y}\hat{I}_{2,y}$, if $\phi = \pi/2$ or $3\pi/2$, then to $4\hat{S}_z\hat{I}_{1,x}\hat{I}_{2,x}$. Brackets multiplication gives

$$\begin{aligned} 4\hat{S}_z\hat{I}_{1,z}\hat{I}_{2,z} \xrightarrow{\frac{\pi}{2}(\hat{I}_{1,\phi}+\hat{I}_{2,\phi})} \; & \hat{S}_z\hat{I}_1^+\hat{I}_2^+ e^{-i\mathbf{2}(\phi-\pi/2)} && \backslash\backslash\ \text{DQ} \\ & \hat{S}_z\hat{I}_1^+\hat{I}_2^- e^{-i\mathbf{0}(\phi-\pi/2)} && \backslash\backslash\ \text{ZQ} \\ & \hat{S}_z\hat{I}_1^-\hat{I}_2^+ e^{-i\mathbf{0}(\phi-\pi/2)} && \backslash\backslash\ \text{ZQ} \\ & \hat{S}_z\hat{I}_1^-\hat{I}_2^- e^{-i(\mathbf{-2})(\phi-\pi/2)} && \backslash\backslash\ \text{DQ} \end{aligned} \quad \text{(S35)}$$

Eq. (S35) features excitation of *zero-* (ZQ) and *double-quantum coherences* (DQ) from a spin order term with two nuclei. The obtained coherence operators can be equivalently denoted by the symbol $\hat{S}_z\hat{I}_X^{\mathbf{s}}$, where $\mathbf{s}$ is an

N-element array with $+1$, -1 and 0. Zero at position i means that spin i is not involved in the coherence, otherwise $+1$ is for $\hat{I}_i^+$ and -1 is for $\hat{I}_i^-$. In principle, $\mathbf{s}$ already contains information about X, because only indices from X can be non-zero in $\mathbf{s}$. Nevertheless, keeping X explicitly simplifies the following analysis. The number $p(\mathbf{s}) = s_+ - s_-$ (bold in the equation above), algebraic sum of numbers in $\mathbf{s}$, alternatively, the difference between the amount of raising and lowering operators, is called the coherence order (*71,72*). This is an integer between $-|X| \leqslant p(\mathbf{s}) \leqslant |X|$. Later on, we will omit the symbol $\mathbf{s}$ for brevity. Coherence order determines the transformation of a coherence element upon z-rotation

$$\exp(-i\theta\hat{F}_z)\hat{I}_X^{\mathbf{s}}\exp(i\theta\hat{F}_z) = e^{-ip(\mathbf{s})\theta}\hat{I}_X^{\mathbf{s}} \tag{S36}$$

which generalizes Eq. (S7).

The excitation of a general nuclear spin order $\hat{S}_z\hat{I}_{X,z}$ can be described by the formula (S37) (also, Eq. (9) in the main text):

$$2^{|X|}\hat{S}_z\hat{I}_{X,z} \xrightarrow{\frac{\pi}{2}\hat{F}_\phi} \sum_{\mathbf{s}(X)} e^{-ip(\phi-\pi/2)}\hat{S}_z\hat{I}_X^{\mathbf{s}} \tag{S37}$$

where $\hat{F}_\phi = \hat{I}_{1,\phi} + ... + \hat{I}_{N,\phi}$ and $\mathbf{s}(X)$ is a coherence signature corresponding to subset $X \subseteq \overline{1,N}$. To better understand the detection part of our ENDOR experiments, it is useful to consider the generalization of Eq. (S11). It can be shown

$$\hat{S}_z\hat{I}_X^{\mathbf{s}} \xrightarrow{\frac{\pi}{2}\hat{F}_\phi} e^{ip(\phi+\pi/2)}\hat{S}_z\hat{I}_{X,z} + \hat{\sigma}_{\text{rest}} \tag{S38}$$

where p is again short for $p(\mathbf{s})$ and $\hat{\sigma}_{\text{rest}}$ contains terms non-contributing to the electron spin echo.

Now, we return to Eq. (S37) and specify it for the $+y$ pulse ($\phi = \pi/2$):

$$2^{|X|}\hat{S}_z\hat{I}_{X,z} \xrightarrow{\frac{\pi}{2}\hat{F}_y} 2^{|X|}\hat{S}_z\hat{I}_{X,x} = \sum_{\mathbf{s}(X)} \hat{S}_z\hat{I}_X^{\mathbf{s}}\,. \tag{S39}$$

For two spins, Eq. (S39) reads

$$\begin{aligned}
&X = \{\} && 2^0\hat{S}_z \to \hat{S}_z \\
&X = \{1\} && 2^1\hat{S}_z\hat{I}_{1,z} \to \hat{S}_z\hat{I}_1^+ + \hat{S}_z\hat{I}_1^- \\
&X = \{2\} && 2^1\hat{S}_z\hat{I}_{2,z} \to \hat{S}_z\hat{I}_2^+ + \hat{S}_z\hat{I}_2^- \\
&X = \{1,2\} && 2^2\hat{S}_z\hat{I}_{1,z}\hat{I}_{2,z} \to \hat{S}_z\hat{I}_1^+\hat{I}_2^+ + \hat{S}_z\hat{I}_1^+\hat{I}_2^- + \hat{S}_z\hat{I}_1^-\hat{I}_2^+ + \hat{S}_z\hat{I}_1^-\hat{I}_2^-
\end{aligned} \tag{S40}$$

where the vectors $\mathbf{s}$ are (from top to bottom) $(0,0)$; $(+1,0)$ and $(-1,0)$; $(0,+1)$ and $(0,-1)$; $(+1,+1)$ and $(+1,-1)$ and $(-1,+1)$ and $(-1,-1)$.

The evolution of these coherence elements can be computed using the rules from Section S1.3. Table S1 contains specific results for $\hat{\sigma} = \hat{S}_z\hat{I}_1^+\hat{I}_2^-$.

Table S1: **Example of coherence evolution.** Evolution of the coherence element $\hat{S}_z\hat{I}_1^+\hat{I}_2^-$ under relevant interactions: chemical shift, hyperfine coupling and nuclear-nuclear coupling.

Term in $\hat{H}$	Evolution of $\hat{S}_z\hat{I}_1^+\hat{I}_2^-$
$\Delta\omega_1\hat{I}_{1,z} + \Delta\omega_2\hat{I}_{2,z}$	$e^{-i(\Delta\omega_1-\Delta\omega_2)t}\cdot\hat{S}_z\hat{I}_1^+\hat{I}_2^-$
$A_1\hat{S}_z\hat{I}_{1,z} + A_2\hat{S}_z\hat{I}_{2,z}$	$e^{-i(A_1-A_2)t\hat{S}_z}\cdot\hat{S}_z\hat{I}_1^+\hat{I}_2^-$
$\omega_{1,2}\hat{I}_{1,z}\hat{I}_{2,z}$	$\hat{S}_z\hat{I}_1^+\hat{I}_2^-$
$\omega_{3,4}\hat{I}_{3,z}\hat{I}_{4,z}$	$\hat{S}_z\hat{I}_1^+\hat{I}_2^-$
$\omega_{1,4}\hat{I}_{1,z}\hat{I}_{4,z}$	$e^{-i\omega_{1,4}t\hat{I}_{4,z}}\hat{S}_z\hat{I}_1^+\hat{I}_2^-$
$\omega_{2,4}\hat{I}_{2,z}\hat{I}_{4,z}$	$e^{+i\omega_{2,4}t\hat{I}_{4,z}}\hat{S}_z\hat{I}_1^+\hat{I}_2^-$

Chemical shift (line 1) induces the phase evolution. Hyperfine coupling (line 2) makes the given coherence oscillate between $\hat{S}_z\hat{I}_1^+\hat{I}_2^-$ and $\hat{I}_1^+\hat{I}_2^-$ (the latter being unobservable in ENDOR). Evolution under nuclear coupling

(lines 3-5) depends on the coherence composition. If both coupled nuclei are simultaneously excited or *not* excited in a given coherence, then their coupling does not affect its evolution. This is a general statement that the evolution of multiple-quantum coherence is not affected by the coupling between the nuclei involved. Coupling with non-involved nuclei leads to the evolution, as exemplified in line 5.

These examples can be generalised to describe the evolution of an arbitrary coherence element as follows (Eq. (10) in the main text):

$$\hat{S}_z\hat{I}_X^{\mathbf{s}} \xrightarrow{\hat{H}_{\mathrm{CS}}t} e^{-i\boldsymbol{\Delta\omega}\cdot\mathbf{s}t}\cdot\hat{S}_z\hat{I}_X^{\mathbf{s}} \qquad \text{(chemical shift)} \tag{S41}$$

$$\hat{S}_z\hat{I}_X^{\mathbf{s}} \xrightarrow{\hat{H}_{\mathrm{hf}}t} e^{-i\mathbf{A}\cdot\mathbf{s}t\hat{S}_z}\cdot\hat{S}_z\hat{I}_X^{\mathbf{s}} \qquad \text{(hyperfine coupling)} \tag{S42}$$

$$\hat{S}_z\hat{I}_X^{\mathbf{s}} \xrightarrow{\hat{H}_{\mathrm{nd}}t} \prod_{k\notin X} e^{-i\boldsymbol{\omega}_k\cdot\mathbf{s}t\hat{I}_{k,z}}\cdot\hat{S}_z\hat{I}_X^{\mathbf{s}} \qquad \text{(nuclear coupling)} \tag{S43}$$

where $\boldsymbol{\omega}_k$ are the columns of the nuclear-nuclear coupling matrix (see Section S1.1), and $\mathbf{A}\cdot\mathbf{s}$, $\boldsymbol{\Delta\omega}\cdot\mathbf{s}$ and $\boldsymbol{\omega}_k\cdot\mathbf{s}$ are each the scalar product of two N-dimensional arrays.

Here, we behold the clear advantage of working in the spherical basis. All three equations have a similar meaning: the evolution of a coherence element is described by a multiplication by an operator U (not to be confused with a real propagator) that has a universal structure *for all* X and $\mathbf{s}$:

$$\hat{S}_z\hat{I}_X^{\mathbf{s}} \xrightarrow{\hat{H}_i t} U_i(X,\mathbf{s},t)\hat{S}_z\hat{I}_X^{\mathbf{s}}\,. \tag{S44}$$

We emphasize that the evolution in Eqs. (S41)–(S43) preserves the coherence structure. This means that the raising and lowering operators are persistent in the result. In the linear form, only multiplication by $\hat{I}_z$ operators from non-involved nuclei and multiplication by $\hat{S}_z$ can be found.

Note that since the Hamiltonians commute, rules (S41)–(S43) can be applied in any order. Operators U also commute, they are multiplicative in the time argument $U(X,\mathbf{s},t_1)U(X,\mathbf{s},t_2) = U(X,\mathbf{s},t_1+t_2)$ and have a sign inversion symmetry $U(X,\mathbf{s},-t) = U(X,-\mathbf{s},t)$, which will be used in the application to NEEM and PDHS in Section S1.9.

S1.8 Detection

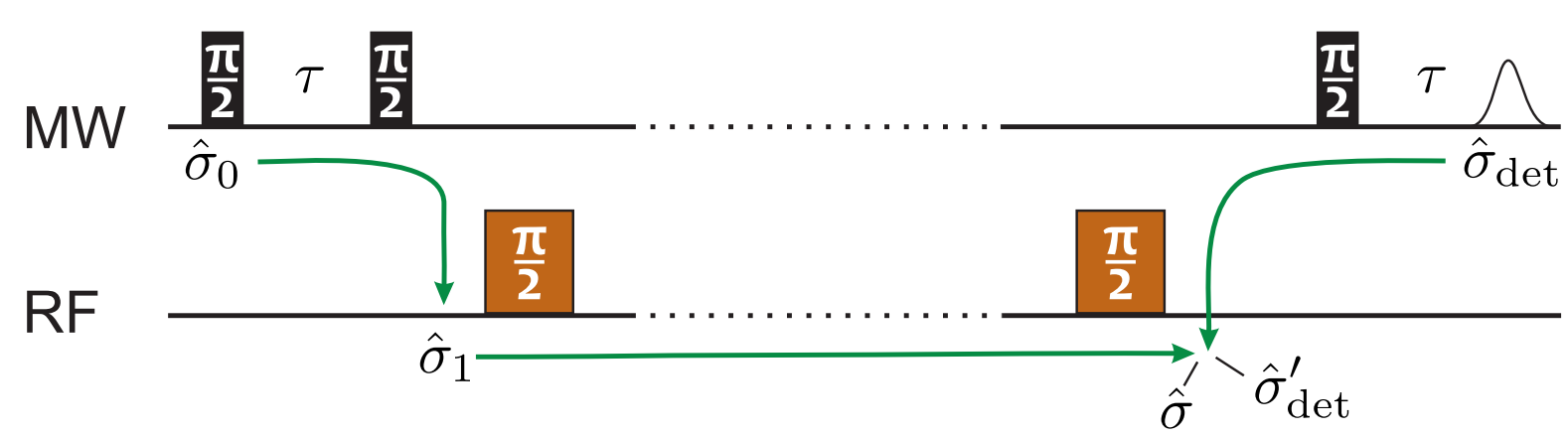

Figure S2: **Backpropagation of the detection operator.** This step simplifies the signal computation. σ_{det} can be $\hat{S}_x$ and $\hat{S}_y$.

Here, we assume that the evolution of the spin system is computed until the last MW $\pi/2$ pulse ($\hat{\sigma}$ in Fig. S2). Instead of further propagating the density matrix to the detection point, we backpropagate the EPR detection operator ($\hat{\sigma}_{\mathrm{det}} = \hat{S}_x$) to the current point of the pulse sequence ($\hat{\sigma}'_{\mathrm{det}}$ in Fig. S2). Backpropagation can be computed quickly because the detection is symmetric with respect to the generation part. At the same time, as we focus on the formula for the final signal, this step allows us to discard non-detectable components of the density matrix $\hat{\sigma}$ and prevent their meaningless forward propagation to the point of the electron echo.

As argued above, $\hat{\sigma}'_{\mathrm{det}} = \hat{\sigma}_1(-\tau)$, where $\hat{\sigma}_1(\tau)$ is the density matrix after the preparation block of length τ (marked in Figure S2), so it is written as

$$\hat{\sigma}'_{\mathrm{det}} = \sum_{X\subseteq\overline{1,N}} c_g(X,-\tau)2^{|X|}\hat{S}_z\hat{I}_{X,z} \tag{S45}$$

This means that, after computing the evolution of the density matrix during the mixing block, we are only interested in the elements generated after the Mims preparation block. Interestingly, we can already predict, at this step, which terms in $\hat{\sigma}$ before the last RF $\pi/2$ pulse become detectable after the pulse using the result in Eq. (S38). Indeed, if we do one more backpropagation step of $\hat{\sigma}_{\mathrm{det}}$, i.e."pull" it through the RF $\pi/2$ pulse, it would become the combination of coherences as in Eq. (S37). Conversely, only terms like $\hat{S}_z\hat{I}_X^{\mathbf{s}}$ can become $\hat{S}_z\hat{I}_{X,z}$ upon the RF $\pi/2$ pulse; therefore, we will focus on them in the next Section. Understanding of this will simplify the analysis of the specific pulse sequences.

Different terms in $\hat{\sigma}_{\mathrm{det}}$ written in the spherical basis are pairwise orthogonal. Thus, based on everything we learned so far, the formation of signal TD ENDOR can be split into three functional blocks:

- **Generation**. The initial density matrix $\hat{S}_x$ is converted into various nuclear spin order operators $2^{|X|}\hat{S}_z\hat{I}_{X,z}$ with weights $c_g(X,\tau)$.
- **Mixing**. Upon nuclear excitation and manipulation, the operators $2^{|X|}\hat{S}_z\hat{I}_{X,z}$ are transformed into different forms, of which only the detectable ones are $2^{|Y|}\hat{S}_z\hat{I}_{Y,z}$ for a possibly different set Y. The conversion coefficient is denoted as $m(X \to Y)$.
- **Detection**. Operators $2^{|Y|}\hat{S}_z\hat{I}_{Y,z}$ are converted to $\hat{S}_x$ (electron spin magnetization) with the coefficient $c_g(Y,\tau)$ and physically measured with the EPR spectrometer. The negative sign of τ disappears because after detectable elements have been identified in Eq. (S45), their evolution to the $\hat{S}_x$ is a forward time propagation.

These transformations in the density matrix are called *pathways* here. The EPR signal is the algebraic sum of individual pathways' contributions, i.e.

$$V = \sum_{X,Y} V_{X,Y} = \sum_{X,Y} c_g(X,\tau)m(X \to Y)c_g(Y,\tau)\,. \tag{S46}$$

Prospectively, we can reduce this formula for NEEM and PDHS to

$$V = \sum_{X} V_{X,X} = \sum_{X} c_g(X,\tau)^2 m(X \to X) \tag{S47}$$

since no shape transformation of spin orders occurs in these experiments, i.e. $m(X \to Y) = 0$ for $X \neq Y$. The computation of the remaining mixing coefficients $m(X \to X)$ is based on Eqs. (S41)–(S43) as demonstrated in the next Section.

S1.9 Example of the signal derivation in TD ENDOR with multiple nuclei

In this section, we provide a step-by-step derivation of the signals in NEEM and PDHS for any N, followed by a specification for the case $N = 2$. We start from the excitation by the first RF $\pi/2$ pulse. Let's consider the evolution of an arbitrary coherence element described by the nuclear subset $X \subseteq \overline{1,N}$ and signature $\mathbf{s}$ according to rules (S41)–(S43)

$$\hat{S}_z\hat{I}_X^{\mathbf{s}} \xrightarrow{\hat{H}t} \underbrace{e^{-i\boldsymbol{\Delta\omega}\cdot\mathbf{s}t}}_{\hat{H}_{\mathrm{CS}}}\underbrace{e^{-i\mathbf{A}\cdot\mathbf{s}t\hat{S}_z}}_{\hat{H}_{\mathrm{hf}}}\underbrace{\prod_{k\notin X} e^{-i\boldsymbol{\omega}_k\cdot\mathbf{s}t\hat{I}_{k,z}}}_{\hat{H}_{\mathrm{nd}}}\hat{S}_z\hat{I}_X^{\mathbf{s}} = U_{\mathrm{CS}}(X,\mathbf{s},t)U_{\mathrm{hf}}(X,\mathbf{s},t)U_{\mathrm{nd}}(X,\mathbf{s},t)\hat{S}_z\hat{I}_X^{\mathbf{s}}\,. \tag{S48}$$

This is a shared beginning of NEEM and PDHS. In the following subsections, we consider these two experiments separately.

S1.9.1 Nuclear echo envelope modulation (NEEM)

The first period of free evolution is $t = \tau_{\mathrm{RF}}$. Next, the RF π pulse with phase ψ does not change U_{CS} or U_{hf} but causes the inversion $\hat{I}_{k,z} \to -\hat{I}_{k,z}$ in U_{nd}. Luckily for the following steps, such an inversion is formally equivalent to a sign inversion of vector $\mathbf{s}$. Consequently, we can write

$$\xrightarrow{\pi\hat{F}_\psi} e^{i2p\psi}U_{\mathrm{CS}}(X,\mathbf{s},t)U_{\mathrm{hf}}(X,\mathbf{s},t)U_{\mathrm{nd}}(X,-\mathbf{s},t)\hat{S}_z\hat{I}_X^{-\mathbf{s}}\,. \tag{S49}$$

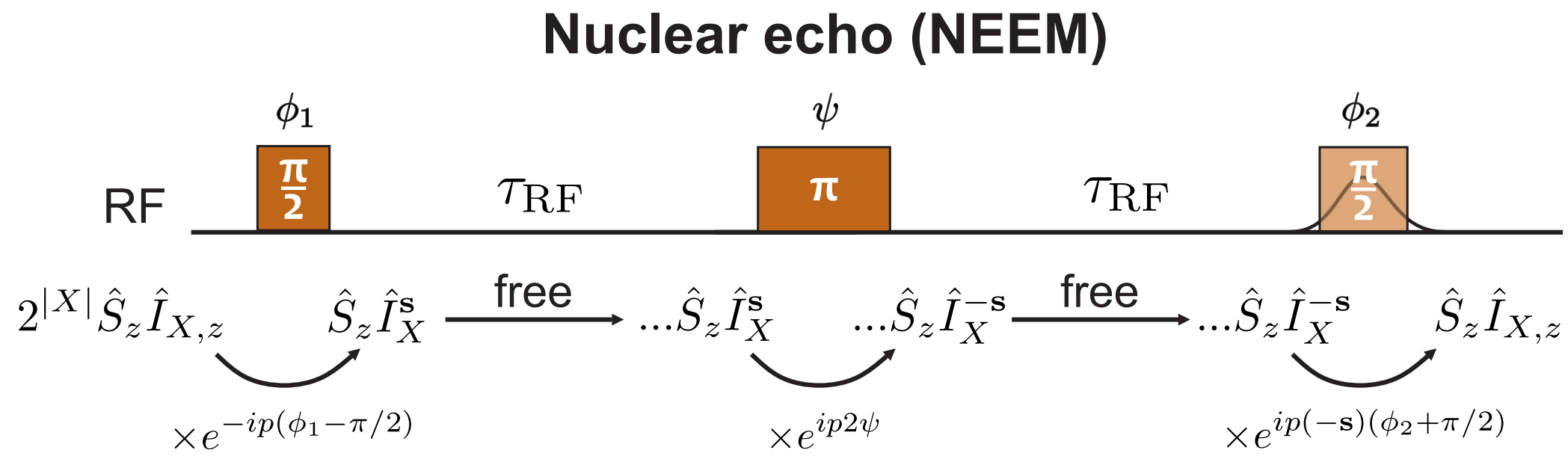

Figure S3: **Density matrix evolution in the nuclear echo experiment.** 'free' denotes free evolution. Only the path leading to a detectable term is outlined. Dots substitute a numeric coefficient. Phases of RF pulses are assumed arbitrary: ϕ_1, ψ and ϕ_2. These phases affect the state under consideration through phase factors (indicated by curved arrows). p is used for $p(\mathbf{s})$ but note that $p(-\mathbf{s}) = -p(\mathbf{s}) = -p$.

The following free evolution of this state, using the rules in Eqs. (S41)–(S43), leads to

$$U_{\rm nd}(X,-\mathbf{s},\tau_{\rm RF})\,\overbrace{U_{\rm hf}(X,-\mathbf{s},\tau_{\rm RF})\,\underbrace{U_{\rm CS}(X,-\mathbf{s},\tau_{\rm RF})U_{\rm CS}(X,\mathbf{s},\tau_{\rm RF})}_{=1}\,U_{\rm hf}(X,\mathbf{s},\tau_{\rm RF})}^{=1}\,U_{\rm nd}(X,-\mathbf{s},\tau_{\rm RF})\hat{S}_z\hat{I}_X^{-\mathbf{s}}\,. \tag{S50}$$

Thanks to the relations $U_{\rm CS}(X,-\mathbf{s},\tau_{\rm RF})U_{\rm CS}(X,\mathbf{s},\tau_{\rm RF}) = 1$ and $U_{\rm hf}(X,-\mathbf{s},\tau_{\rm RF})U_{\rm hf}(X,\mathbf{s},\tau_{\rm RF}) = 1$, this expression simplifies to

$$\hat{\sigma}' = U_{\rm nd}(X,-\mathbf{s},2\tau_{\rm RF})\hat{S}_z\hat{I}_X^{-\mathbf{s}} = \left[\prod_{k\notin X}\exp\left(-i\boldsymbol{\omega}_k\cdot\mathbf{s}2\tau_{\rm RF}\hat{I}_{k,z}\right)\right]\hat{S}_z\hat{I}_X^{-\mathbf{s}}\,. \tag{S51}$$

As expected, chemical shift and hyperfine coupling are refocused at the nuclear echo.

Now, we substitute Eq. (S21) and recognise that all terms with $\hat{I}_z$ are not detectable because the last RF $\pi/2$ pulse converts them into coherences. After completing the multiplication, we find only one detectable term, i.e.

$$\hat{\sigma}'_{\rm NEEM} = \left[\prod_{k\notin X}\cos\left(\boldsymbol{\omega}_k\cdot\mathbf{s}\tau_{\rm RF}\right)\right]\hat{S}_z\hat{I}_X^{-\mathbf{s}} + \text{non-detectable terms}\,. \tag{S52}$$

The first term in this equation is converted to the detectable spin order with the phase factor $\exp(ip(-\mathbf{s})(\phi_2 + \pi/2)) = \exp(-ip(\mathbf{s})(\phi_2 + \pi/2))$ (see Eq. (S11). We also recall that coherence was at first generated with the factor $\exp(-ip(\mathbf{s})(\phi_1 + \pi/2))$ and multiplied upon the RF π pulse with $\exp(ip(\mathbf{s})2\psi)$. Overall, the phase factor $\exp(-ip\Phi)$ is gained, where $\Phi = \phi_1 + \phi_2 - 2\psi$. The total mixing coefficient from $2^{|X|}\hat{S}_z\hat{I}_{X,z}$ to $2^{|X|}\hat{S}_z\hat{I}_{X,z}$ (Figure S3) is the sum over all coherence signatures $\mathbf{s}$

$$m(X\to X;\tau_{\rm RF}) = 2^{-|X|}\sum_{\mathbf{s}(X)}\cos p\Phi\prod_{k\notin X}\cos(\boldsymbol{\omega}_k\cdot\mathbf{s}\tau_{\rm RF})\,. \tag{S53}$$

Note that p depends on the array $\mathbf{s}$. Terms proportional to $\cos p\Phi$ are called *p-quantum contributions* and can be experimentally extracted by a p-quantum phase cycling filter. To obtain the total ENDOR signal, the mixing coefficients are multiplied by their corresponding blind spot functions, and the results are summed (see Eq. (S47)).

Note that in NEEM and PDHS, no change of nuclear spin orders occurs, i.e. evolution of $\hat{S}_z\hat{I}_{X,z}$ in these experiments leads to $\hat{S}_z\hat{I}_{X,z}$ only, among detectable terms.

S1.9.2 Specification for $N = 2$

In the case of two spins, the nuclear coupling arrays are as follows

$$\boldsymbol{\omega}_1 = \begin{pmatrix}0\\ \omega_{1,2}\end{pmatrix} \qquad\qquad \boldsymbol{\omega}_2 = \begin{pmatrix}\omega_{1,2}\\ 0\end{pmatrix} \tag{S54}$$

Next, we compute four mixing coefficients according to the formula (S53).

- Case $X = \{\}$, $|X| = 0$. The only possible signature $\mathbf{s}$ is $(0,0)$ with $p = 0$; therefore the sum has only one term $\cos 0\Phi \cos((\boldsymbol{\omega_1}\cdot(0,0))\tau_{\mathrm{RF}}) \cos((\boldsymbol{\omega_2}\cdot(0,0))\tau_{\mathrm{RF}})$, which is equal to 1 as all scalar products are zero;
- Case $X = \{1\}$, $|X| = 1$. $\mathbf{s}$ can be $(+1,0)$ with $p = +1$ and $(-1,0)$ with $p = -1$. The mixing coefficient is equal to $2^{-1}(\cos\Phi\cos(+\omega_{1,2}\tau_{\mathrm{RF}}) + \cos(-\Phi)\cos(-\omega_{1,2}\tau_{\mathrm{RF}})) = \cos\Phi\cos(\omega_{1,2}\tau_{\mathrm{RF}})$;
- Case $X = \{2\}$, $|X| = 1$. Similarly to the previous case, we obtain $\cos\Phi\cos(\omega_{1,2}\tau_{\mathrm{RF}})$;
- Case $X = \{1,2\}$, $|X| = 2$. The sum has four terms, but there are no indices $k \notin X$; therefore, the product is always "empty", i.e. equal to 1. Finally, we obtain $2^{-2}(\cos 2\Phi + \cos 0\Phi + \cos 0\Phi + \cos(-2\Phi)) = \frac{1}{2}(1 + \cos 2\Phi)$.

These results are summarised in Table S2.

Table S2: **Building the analytical expression for NEEM ($N = 2$).** Mixing coefficients for different nuclear spin orders $2^{|X|}\hat{S}_z\hat{I}_{X,z}$ in nuclear echo experiment ($N = 2$). These expressions show that chemical shift and hyperfine interactions are refocused, while nuclear-nuclear coupling modulates single-quantum terms.

Subset X	Mixing coefficient $m(X \to X; \tau_{\mathrm{RF}})$	Blind spot function $c_g(X,\tau)^2$
$\{\}$	1	$\cos^2\frac{A_1\tau}{2}\cos^2\frac{A_2\tau}{2}$
$\{1\}$	$\cos\Phi\cos\omega_{1,2}\tau_{\mathrm{RF}}$	$\sin^2\frac{A_1\tau}{2}\cos^2\frac{A_2\tau}{2}$
$\{2\}$	$\cos\Phi\cos\omega_{1,2}\tau_{\mathrm{RF}}$	$\cos^2\frac{A_1\tau}{2}\sin^2\frac{A_2\tau}{2}$
$\{1,2\}$	$\frac{1}{2}(1+\cos 2\Phi)$	$\sin^2\frac{A_1\tau}{2}\sin^2\frac{A_2\tau}{2}$

S1.9.3 Specification for $N = 3$

The double-quantum nuclear coherence in the $N = 2$ case does not evolve under nuclear coupling; however, it does evolve with $N = 3$ due to the coupling with the third nucleus. Formula (S53) automatically demonstrates this. Indeed, the nuclear coupling constants are

$$\boldsymbol{\omega}_1 = \begin{pmatrix} 0 \\ \omega_{1,2} \\ \omega_{1,3} \end{pmatrix} \qquad \boldsymbol{\omega}_2 = \begin{pmatrix} \omega_{1,2} \\ 0 \\ \omega_{2,3} \end{pmatrix} \qquad \boldsymbol{\omega}_3 = \begin{pmatrix} \omega_{1,3} \\ \omega_{2,3} \\ 0 \end{pmatrix} \tag{S55}$$

Now, if $X = \{1,2\}$ ($|X| = 2$), index k in Eq. (S53) attains $k = 3$. In all arrays $\mathbf{s}(X)$, $s_3 = 0$, therefore,

$$\boldsymbol{\omega}_3 \cdot \mathbf{s} = \omega_{1,3}s_1 + \omega_{2,3}s_2 \tag{S56}$$

and the mixing amplitude for $X = \{1,2\}$ is

$$m(X \to X, \tau_{\mathrm{RF}}) = \frac{1}{4}(2\cos(\omega_{1,3} - \omega_{2,3})\tau_{\mathrm{RF}} + 2\cos 2\Phi\cos(\omega_{1,3} + \omega_{2,3})\tau_{\mathrm{RF}}) \tag{S57}$$

where we observe the modulation of the double-quantum term. Other mixing terms can be obtained similarly.

As a result, the general formula for NEEM with $N=3$ is ($\Phi = \phi_1 + \phi_2 - 2\psi$)

$$\begin{aligned}
V(\tau_{\mathrm{RF}}) = &+ \cos^2\frac{A_1\tau}{2}\cos^2\frac{A_2\tau}{2}\cos^2\frac{A_3\tau}{2} \\
&+ \sin^2\frac{A_1\tau}{2}\cos^2\frac{A_2\tau}{2}\cos^2\frac{A_3\tau}{2}\cos\Phi\cos(\omega_{1,2}\tau_{\mathrm{RF}})\cos(\omega_{1,3}\tau_{\mathrm{RF}}) \\
&+ \cos^2\frac{A_1\tau}{2}\sin^2\frac{A_2\tau}{2}\cos^2\frac{A_3\tau}{2}\cos\Phi\cos(\omega_{1,2}\tau_{\mathrm{RF}})\cos(\omega_{2,3}\tau_{\mathrm{RF}}) \\
&+ \cos^2\frac{A_1\tau}{2}\cos^2\frac{A_2\tau}{2}\sin^2\frac{A_3\tau}{2}\cos\Phi\cos(\omega_{1,3}\tau_{\mathrm{RF}})\cos(\omega_{2,3}\tau_{\mathrm{RF}}) \\
&+ \sin^2\frac{A_1\tau}{2}\sin^2\frac{A_2\tau}{2}\cos^2\frac{A_3\tau}{2}\cdot\frac{1}{2}(\cos 2\Phi\cos(\omega_{1,3}+\omega_{2,3})\tau_{\mathrm{RF}} + \cos(\omega_{1,3}-\omega_{2,3})\tau_{\mathrm{RF}}) \\
&+ \cos^2\frac{A_1\tau}{2}\sin^2\frac{A_2\tau}{2}\sin^2\frac{A_3\tau}{2}\cdot\frac{1}{2}(\cos 2\Phi\cos(\omega_{1,2}+\omega_{1,3})\tau_{\mathrm{RF}} + \cos(\omega_{1,2}-\omega_{1,3})\tau_{\mathrm{RF}}) \\
&+ \sin^2\frac{A_1\tau}{2}\cos^2\frac{A_2\tau}{2}\sin^2\frac{A_3\tau}{2}\cdot\frac{1}{2}(\cos 2\Phi\cos(\omega_{1,2}+\omega_{2,3})\tau_{\mathrm{RF}} + \cos(\omega_{1,2}-\omega_{2,3})\tau_{\mathrm{RF}}) \\
&+ \sin^2\frac{A_1\tau}{2}\sin^2\frac{A_2\tau}{2}\sin^2\frac{A_3\tau}{2}\cdot\frac{1}{4}(\cos 3\Phi + 3\cos\Phi)\,.
\end{aligned} \tag{S58}$$

Note that the SQ-contribution is modulated with a product of cos factors. Due to the identity $\cos\alpha\cos\beta = 1/2(\cos(\alpha+\beta)+\cos(\alpha-\beta))$, this is equivalent to modulation with the sum and difference of nuclear couplings. The DQ-contribution is modulated only with the sum of coupling constants.

S1.9.4 Pulsed dipolar hyperfine spectroscopy (PDHS)

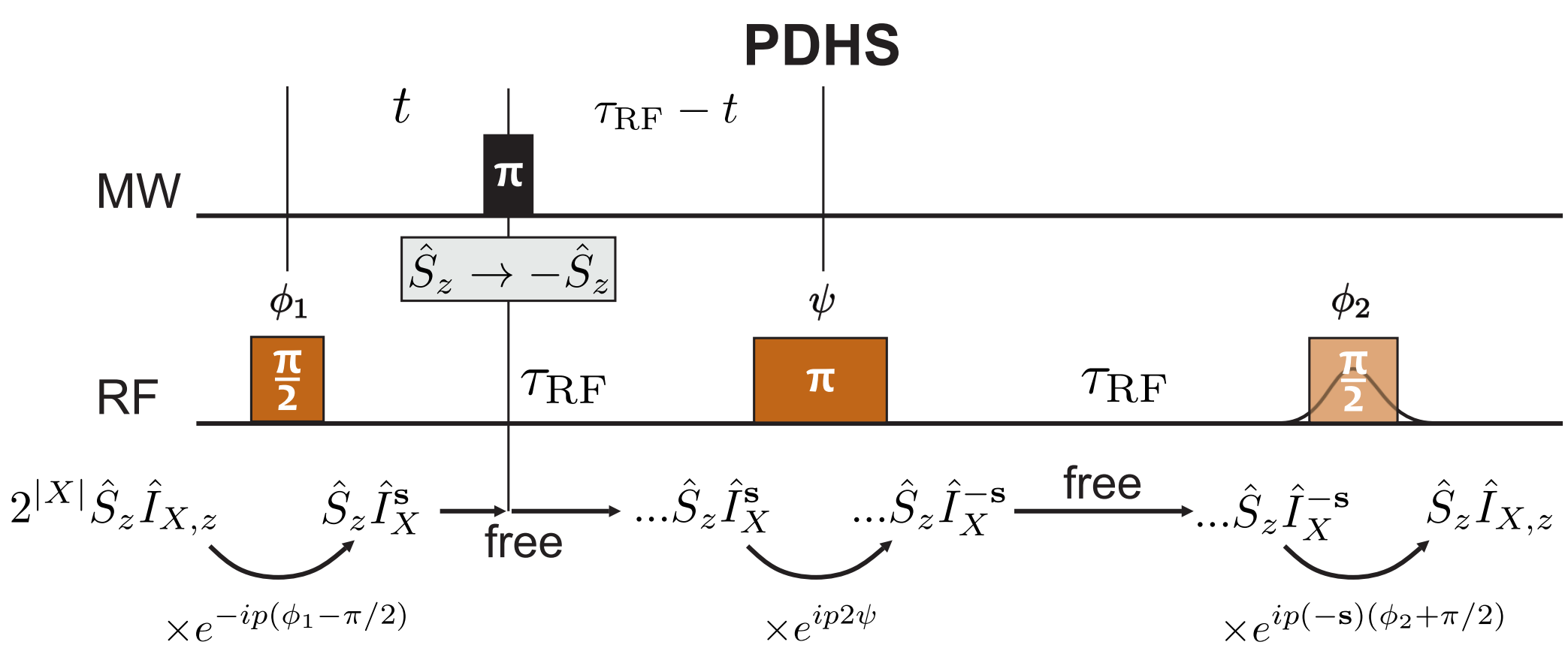

Figure S4: **Density matrix evolution in PDHS experiment.** 'free' denotes free evolution. Phases of RF pulses are assumed arbitrary: ϕ_1, ψ and ϕ_2. The MW π pulse acts here by the sign inversion $\hat{S}_z \to -\hat{S}_z$ which affects the evolution under the hyperfine coupling (U_{hf}).

After free evolution for time t (see Eq. (S48)), a MW π pulse is applied, which is equivalent to inversion $\hat{S}_z \to -\hat{S}_z$. As a result, this pulse inverts $\hat{S}_z\hat{I}_X^{\mathbf{s}} \to -\hat{S}_z\hat{I}_X^{\mathbf{s}}$. Also, it affects the factor $U_{\mathrm{hf}}(X,\mathbf{s},t)$ that becomes formally equal to $U_{\mathrm{hf}}(X,\mathbf{s},-t)$:

$$\xrightarrow{\pi\hat{S}_x} -U_{\mathrm{CS}}(X,\mathbf{s},t)U_{\mathrm{hf}}(X,\mathbf{s},-t)U_{\mathrm{nd}}(X,\mathbf{s},t)\hat{S}_z\hat{I}_X^{\mathbf{s}}\,. \tag{S59}$$

The next free evolution period ($\tau_{\mathrm{RF}} - t$) reads

$$-U_{\mathrm{hf}}(X,\mathbf{s},\tau_{\mathrm{RF}}-t)U_{\mathrm{nd}}(X,\mathbf{s},\tau_{\mathrm{RF}}-t)\underbrace{U_{\mathrm{CS}}(X,\mathbf{s},\tau_{\mathrm{RF}}-t)U_{\mathrm{CS}}(X,\mathbf{s},t)}_{=U_{\mathrm{CS}}(X,\mathbf{s},\tau_{\mathrm{RF}})}U_{\mathrm{nd}}(X,\mathbf{s},t)U_{\mathrm{hf}}(X,\mathbf{s},-t)\hat{S}_z\hat{I}_X^{\mathbf{s}}\,. \tag{S60}$$

If we use the linear property of operators U with respect to time, we obtain

$$-U_{\mathrm{hf}}(X,\mathbf{s},\tau_{\mathrm{RF}}-2t)U_{\mathrm{nd}}(X,\mathbf{s},\tau_{\mathrm{RF}})U_{\mathrm{CS}}(X,\mathbf{s},\tau_{\mathrm{RF}})\hat{S}_z\hat{I}_X^{\mathbf{s}}\,. \tag{S61}$$

The rest of the computation repeats the NEEM experiment:

- RF π pulse adds a factor $e^{ip2\psi}$ and inverts the coherence $\hat{S}_z\hat{I}_X^{\mathbf{s}} \to \hat{S}_z\hat{I}_X^{-\mathbf{s}}$ and affects $U_{\text{nd}}(X, \mathbf{s}, \tau_{\text{RF}}) \to U_{\text{nd}}(X, -\mathbf{s}, \tau_{\text{RF}})$;
- free evolution (τ_{RF}) cancels out $U_{\text{CS}}(X, \mathbf{s}, \tau_{\text{RF}})$ and adds with $U_{\text{nd}}(X, -\mathbf{s}, \tau_{\text{RF}})$.

Given that factor $U_{\text{hf}}(X, \mathbf{s}, \tau_{\text{RF}} - 2t)$ equals to $U_{\text{hf}}(X, -\mathbf{s}, 2t - \tau_{\text{RF}})$, we arrive at

$$-U_{\text{hf}}(X, -\mathbf{s}, 2t)U_{\text{nd}}(X, -\mathbf{s}, 2\tau_{\text{RF}})\hat{S}_z\hat{I}_X^{-\mathbf{s}}\,. \tag{S62}$$

By applying the property (S21) to U_{hf} and U_{nd} and noting that $\hat{S}_z \cdot \hat{S}_z\hat{I}_X^{-\mathbf{s}} = \frac{1}{4}\hat{I}_X^{-\mathbf{s}}$ is non-detectable, we can re-group

$$\hat{\sigma}'_{\text{PDHS}} = e^{-ip(\mathbf{s})(\phi_1 - 2\psi - \pi/2)}\cos(\mathbf{A}\cdot\mathbf{s}t)\left[\prod_{k\notin X}\cos\left(\boldsymbol{\omega}_k\cdot\mathbf{s}\tau_{\text{RF}}\right)\right]\hat{S}_z\hat{I}_X^{-\mathbf{s}} + \text{non-detectable terms}\,. \tag{S63}$$

Finally, after adding the factor $\exp(ip(-\mathbf{s})(\phi_2 + \pi/2))$ from the last RF $\pi/2$ pulse and summing over all $\mathbf{s}$:

$$m(X \to X; t, \tau_{\text{RF}}) = -2^{-|X|}\sum_{\mathbf{s}(X)}\cos p\Phi\cos(\mathbf{A}\cdot\mathbf{s}t)\prod_{k\notin X}\cos(\boldsymbol{\omega}_k\cdot\mathbf{s}\tau_{\text{RF}})\,. \tag{S64}$$

The common negative sign is experimentally meaningful (the electron echo is flipped compared to NEEM), but is further neglected upon signal normalisation. Similarly to NEEM, to obtain the total signal, the mixing coefficients are multiplied by their corresponding blind spot functions, and the results are summed (see Eq. (S47)).

S1.9.5 Specification for $N = 2$

In PDHS, τ_{RF}-dependence of the mixing coefficient repeats that of NEEM (Section S1.9.2). Therefore, we consider only the t-dependence here. By recalling $\mathbf{A} = (A_1, A_2)^T$, we compute the relevant scalar products

$$\begin{pmatrix}A_1\\A_2\end{pmatrix}\cdot\begin{pmatrix}\pm1\\0\end{pmatrix} = \pm A_1 \qquad \begin{pmatrix}A_1\\A_2\end{pmatrix}\cdot\begin{pmatrix}0\\\pm1\end{pmatrix} = \pm A_2 \qquad \begin{pmatrix}A_1\\A_2\end{pmatrix}\cdot\begin{pmatrix}1\\\pm1\end{pmatrix} = A_1 \pm A_2 \tag{S65}$$

and obtain expressions as in Table S3.

Table S3: **Building the analytical expression for PDHS ($N = 2$).** Mixing coefficients for different nuclear spin orders $2^{|X|}\hat{S}_z\hat{I}_{X,z}$ in PDHS experiment ($N = 2$). These expressions show that chemical shifts are refocused, while the hyperfine interaction modulates different terms.

Subset X	Mixing coefficient $m(X \to X; t, \tau_{\text{RF}})$	Blind spot function $c_g(X, \tau)^2$
$\{\}$	1	$\cos^2\frac{A_1\tau}{2}\cos^2\frac{A_2\tau}{2}$
$\{1\}$	$\cos\Phi\cos A_1 t\cos\omega_{1,2}\tau_{\text{RF}}$	$\sin^2\frac{A_1\tau}{2}\cos^2\frac{A_2\tau}{2}$
$\{2\}$	$\cos\Phi\cos A_2 t\cos\omega_{1,2}\tau_{\text{RF}}$	$\cos^2\frac{A_1\tau}{2}\sin^2\frac{A_2\tau}{2}$
$\{1,2\}$	$\frac{1}{2}(\cos(A_1 - A_2)t + \cos 2\Phi\cos(A_1 + A_2)t)$	$\sin^2\frac{A_1\tau}{2}\sin^2\frac{A_2\tau}{2}$

S1.10 Numeric validation for $N = 3$

The spin dynamics analysis in Section S1.9 is exact but relies on less conventional methods than the standard product operator formalism (POF) (*71*). The results for $N = 2$ can be, in principle, validated with POF; however, cases $N > 2$ appear to be more tedious. It is thus interesting to compare formulae (S53) and (S64) with numerically predicted TD ENDOR traces based on the density matrix propagation. We performed this for both PDHS and NEEM using 'random' coupling constants and RF phases to avoid potential error compensation. The specific results are shown in this Section below.

S1.10.1 NEEM

In Figure S5, we validate the analytical expression for NEEM with three nuclei by comparing it with a numerical simulation. The simulation parameters are given in the Figure caption. The peaks in the corresponding spectrum have been assigned to combinations of nuclear couplings ($a = \omega_{1,2}/2\pi$, $b = \omega_{2,3}/2\pi$, $c = \omega_{3,1}/2\pi$). The hyperfine couplings and delay τ impact the peak intensities. Chemical shifts are perfectly refocused in agreement with the formal analysis in Section S1.9.1.

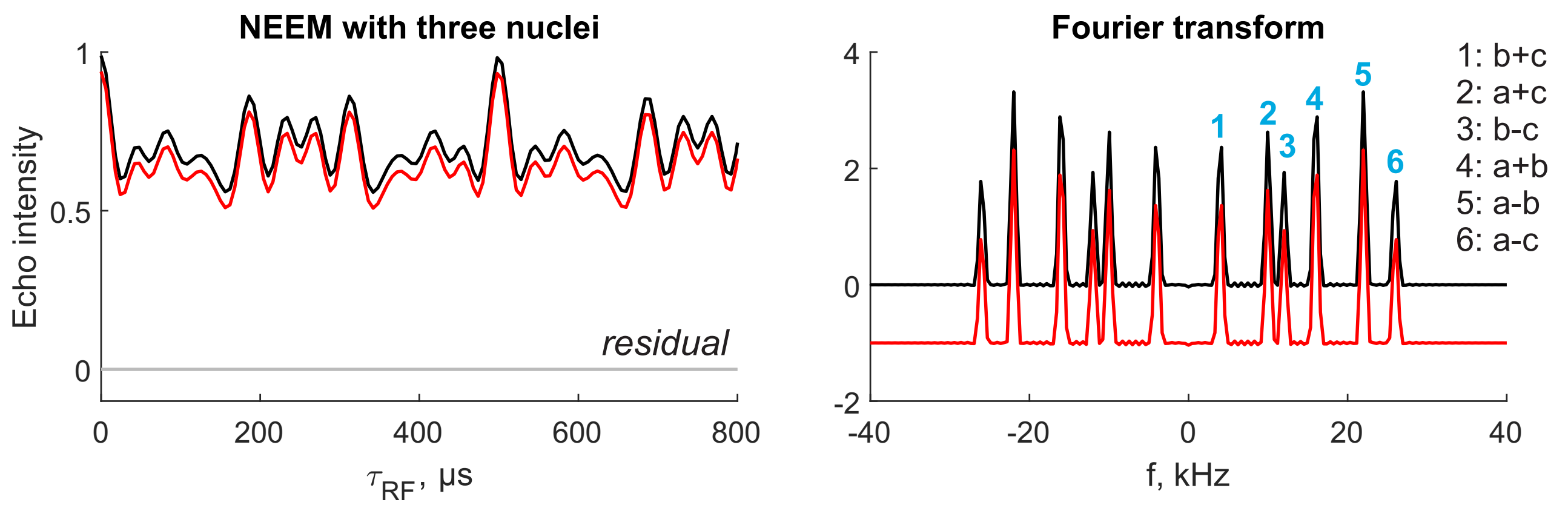

Figure S5: **Numeric validation of NEEM signal with three nuclei.** The black line is computed via the numerical propagation of the density matrix. The red line is the analytical solution. The left panel shows the time-domain signals, and the right panel shows their Fourier transforms. Both datasets exhibit perfect overlap (note the exact zero residuals). The peak assignment in the spectra is shown as numbers 1-6 ($a = \omega_{1,2}/2\pi$, $b = \omega_{2,3}/2\pi$, $c = \omega_{3,1}/2\pi$). Note that the red lines are shifted downwards for better visibility. Simulation parameters: $\tau = 5$ μs, $[A_1, A_2, A_3]/2\pi = [18, 25, 22]$ kHz, $[\omega_{1,2}, \omega_{2,3}, \omega_{3,1}]/2\pi = [-19, 3, -7]$ kHz, $[\Delta\omega_1, \Delta\omega_2, \Delta\omega_3]/2\pi = [1, -2, 3]$ kHz, $[\phi_1, \psi, \phi_2] = [0, \pi/4, \pi/6]$. Since no phase cycling was applied, the signal contains ZQ-, SQ- and DQ-components.

S1.10.2 PDHS

In Figure S6, we validate the analytical expression for PDHS with three nuclei by comparing it with a numerical simulation. The peaks in the corresponding spectrum have been assigned to combinations of hyperfine couplings. The nuclear couplings and delays τ and τ_{RF} affect the peak intensities. Chemical shifts are perfectly refocused.

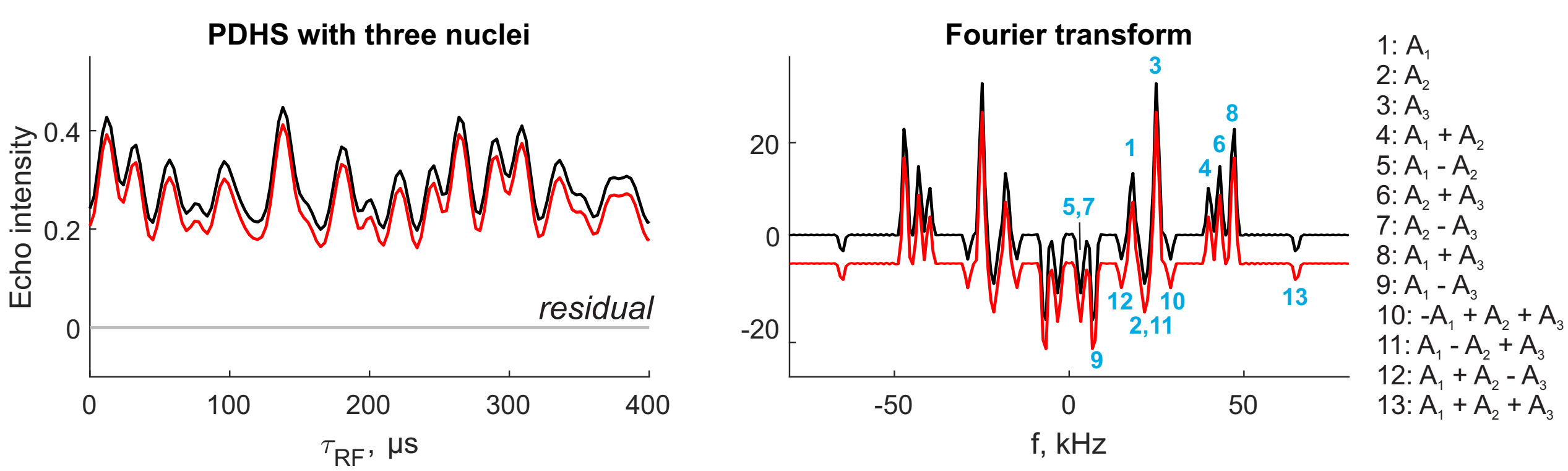

Figure S6: **Numeric validation of PDHS signal with three nuclei.** The black line is computed via the numerical propagation of the density matrix. The red line is the analytical solution. The left panel shows the time-domain signals, and the right panel shows their Fourier transforms. The peak assignment is shown as numbers 1-13. Note that the red lines are shifted downwards for better visibility. Simulation parameters: $\tau = 9$ μs, $[A_1, A_2, A_3]/2\pi = [18, 25, 22]$ kHz, $[\omega_{1,2}, \omega_{2,3}, \omega_{3,1}]/2\pi = [-19, 3, -7]$ kHz, $[\Delta\omega_1, \Delta\omega_2, \Delta\omega_3]/2\pi = [1, -2, 3]$ kHz, $[\phi_1, \psi, \phi_2] = [0, \pi/4, \pi/6]$, $\tau_{RF} = 600$ μs. Since no phase cycling was applied, the signal contains ZQ-, SQ-, DQ- and TQ-components.

S1.11 p-quantum contributions

In Figure S7, we illustrate the distribution of the excited nuclear coherence over different orders $p \geqslant 0$ in NEEM/PDHS. For this, we rewrite Eq. (S47) as

$$V(\tau, \tau_{\mathrm{RF}}) = \sum_{p=-N}^{N} \cos p\Phi \cdot V_p(\tau, \tau_{\mathrm{RF}}), \tag{S66}$$

where V_p is the p-quantum contribution, and formally substitute $\tau_{\mathrm{RF}} = 0$. This allows us to write the following formula based on combinatorial considerations

$$V_p(\tau) = \sum_{X \subseteq \overline{1,N}} c_g(X, \tau)^2 2^{-|X|} n(X, p) \tag{S67}$$

where $n(X, p)$ is the number of coherences of order p that can be excited from spin order $\hat{I}_{X,z}$ (see Eq. (S37)). For example, $n(\{1\}, +1) = n(\{1\}, -1) = 1$, $n(\{1, 2\}, +1) = n(\{1, 2\}, -1) = 0$, $n(\{1, 2\}, 0) = 2$, etc. For $p > N$, $V_p(\tau) = 0$.

We plot the τ-dependence of such p-quantum contributions for systems with $N = 1$ nucleus up to $N = 5$ nuclei, assuming equal hyperfine coupling constants. Since the p-quantum filters do not distinguish between orders p and $-p$, we plot $V_p + V_{-p}$. One can note that the intensity of the single-quantum contribution grows faster with increasing numbers of nuclei. Therefore, multinuclear spin probes can enable single-quantum Mims ENDOR spectroscopy with shorter τ, which is advantageous in terms of electron transverse relaxation and spectral diffusion mechanisms.

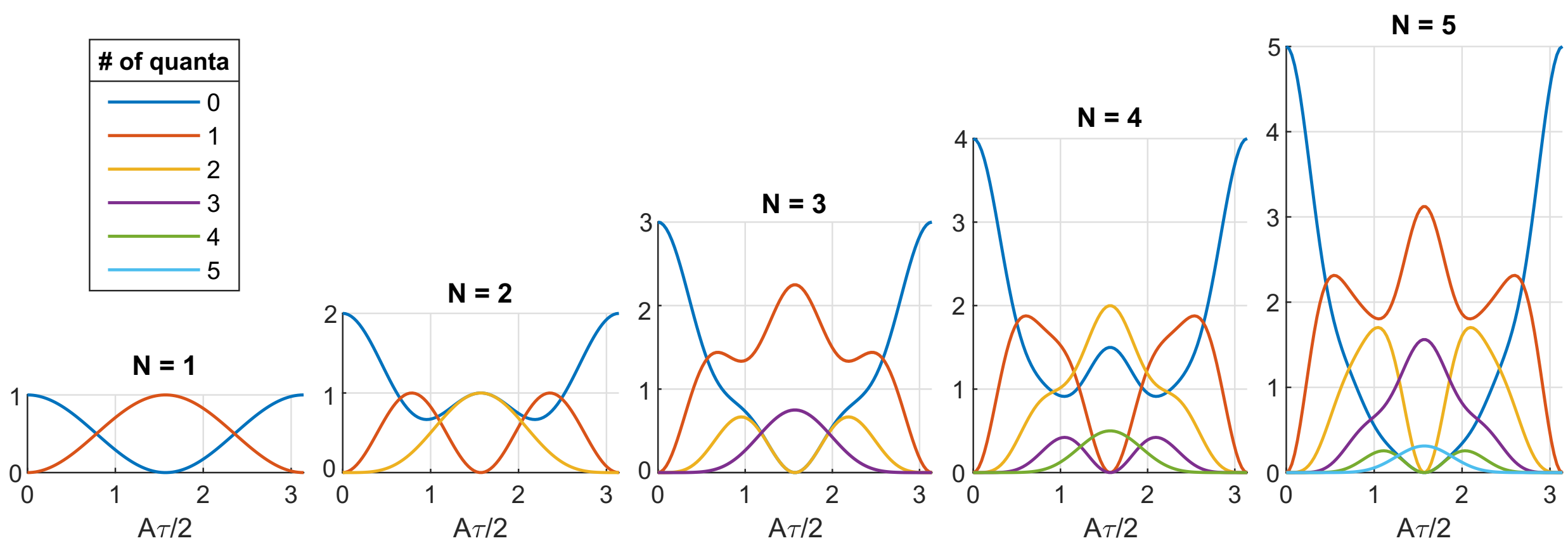

Figure S7: **Relative intensities of p-quantum contributions in time-domain ENDOR experiments as a function τ (Mims preparation block length).** N denotes the number of nuclei. Hyperfine couplings with all nuclei were set to the same value A.

S2 Synthesis

The synthesis of the nitroxides was performed following previously reported procedures (*14*, *29*, *31*, *74-76*). Dicyclohexylcarbodiimide (DCC) and 4-(Trifluoromethyl)phenol were purchased from Sigma-Aldrich. Dimethylaminopyridine (DMAP) was purchased from Acros Organics. 3',5'-Difluoro[1,1'-biphenyl]-4-ol and 2,2'-difluoro[1,1'-biphenyl]-4-ol were purchased from BLDpharm.

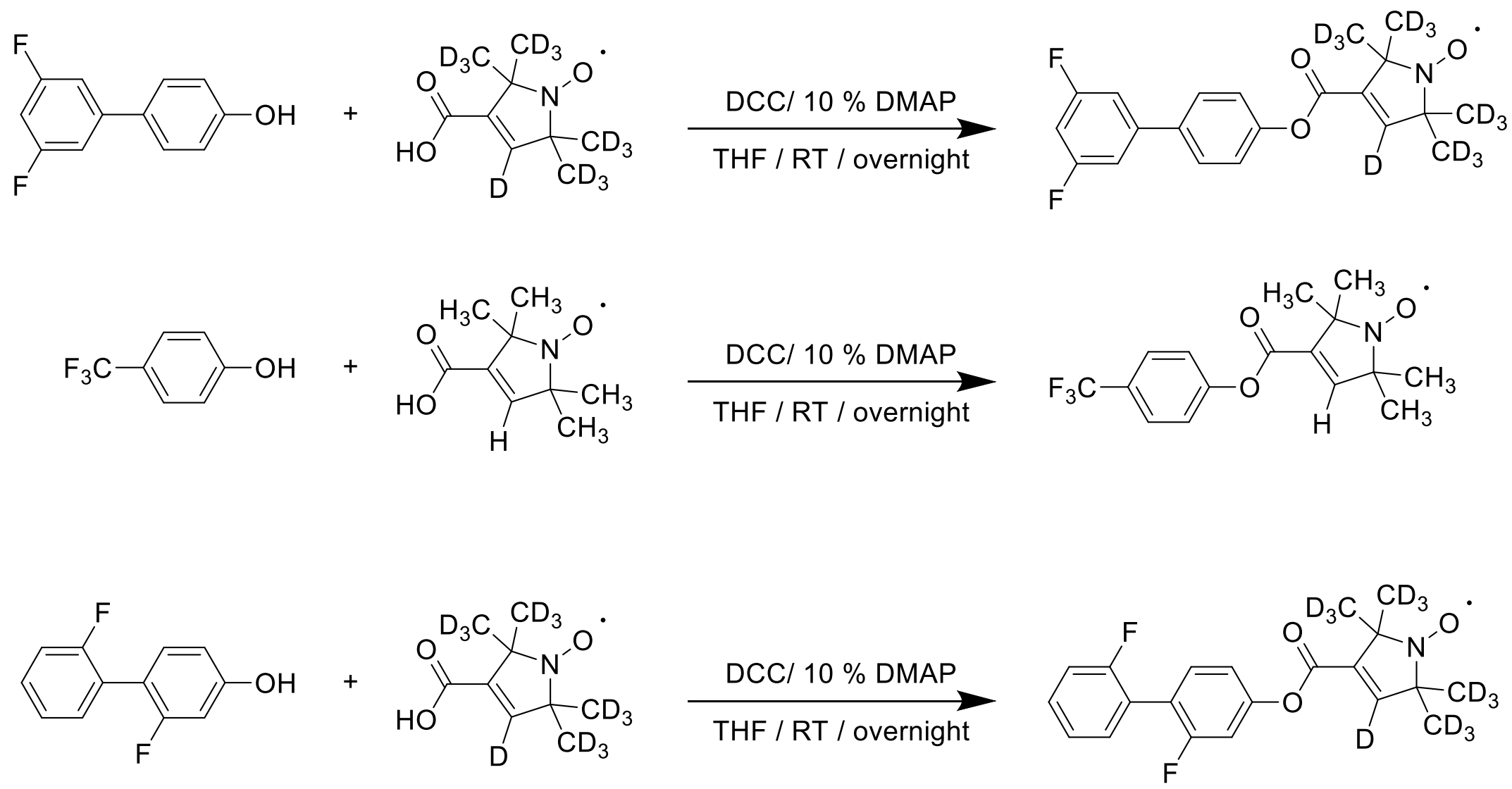

Solvents and all remaining chemicals were purchased from either Sigma-Aldrich, Acros Organics or TCI chemicals. All chemicals were used as received without further purification. The purity of the synthesised compounds was assessed via LC-MS.

S3 DFT optimization

S3.1 Atomic coordinates of RF_2, RFF' and RCF_3

Geometry optimization of compounds $\mathbf{RF_2}$, **RFF'** and $\mathbf{RCF_3}$ was done with Orca 6.0.1 (UKS BP86/def2-tzvp level of theory, CPCM solvent with ε 47.6 and n 1.4). Atomic coordinates (in Å) of optimised structures are listed below.

Compound $\mathbf{RF_2}$

Atom	x	y	z
C	-2.6961989868	0.5966006554	0.0684173374
C	-1.5269447683	1.1633079178	-0.4300739010
C	-2.5904116489	-0.7151589251	0.5213597917
C	-1.4074227731	-1.4422852485	0.4918004048
C	-0.3134144774	0.4900343791	-0.4861112402
C	-0.2432816860	-0.8364941415	-0.0189640059
C	1.0379071812	-1.5837226790	-0.0613834777
C	1.9615616026	-1.3795541232	-1.1048872869
C	1.3610953903	-2.5190848949	0.9394486180
C	2.5651249275	-3.2234526452	0.9066500575
C	3.1649749283	-2.0826112391	-1.1524917651
C	3.4568298639	-2.9974724813	-0.1404836637
F	-3.7115617732	-1.3159576625	1.0096158250
F	-1.5782399933	2.4499800626	-0.8752134233
H	0.5673910585	1.0066347827	-0.8658992136
H	-1.4054188255	-2.4742708341	0.8410510285
H	0.6746333255	-2.6855304808	1.7706861251
H	1.7274747959	-0.6810570513	-1.9095965936
H	3.8647109193	-1.9332196883	-1.9751736186
H	2.8168679810	-3.9410643082	1.6882726574
O	4.6181977910	-3.7834170934	-0.2022823396
C	5.8352163813	-3.1694150814	0.0038010075
O	5.9458485390	-1.9912943103	0.2981584584
C	6.9525238072	-4.1153189486	-0.1644884733
C	6.8347678400	-5.4104251613	-0.5131865327
C	8.1512297392	-6.1211787622	-0.6101028462
C	8.3997064432	-3.6986660949	0.0435607358
N	9.0775254359	-5.0055532738	-0.2474949423
C	8.7132149593	-3.2714522628	1.4878189598
C	8.8841301865	-2.6387211511	-0.9609449430
C	8.4468187102	-6.6215354714	-2.0373840544
C	8.2762304700	-7.2731921689	0.4053070549
O	10.3459100545	-5.1475311702	-0.2007690764
H	-3.6359804947	1.1456868293	0.1022601846
H	5.8954653775	-5.9242531720	-0.7130308877
H	8.0507896391	-6.9294966166	1.4236364717
H	9.2999563312	-7.6717216356	0.3804834674
H	7.5800890180	-8.0803846797	0.1399186037
H	8.3531845393	-5.8077466834	-2.7687601158
H	7.7433843505	-7.4221095193	-2.3035740862
H	9.4681511165	-7.0247028941	-2.0781623254
H	8.6458613963	-2.9342595761	-1.9917806267
H	9.9726568486	-2.5216342792	-0.8673141573
H	8.4068284216	-1.6743654552	-0.7474216773
H	8.3554338058	-4.0207641499	2.2070092173
H	8.2329725148	-2.3095345747	1.7066438984
H	9.7997497367	-3.1564540379	1.6045753689

Compound **RFF'**

Atom	x	y	z
C	-3.3018572423	1.5305127183	-0.0259485885
C	-2.4655045915	2.3763548461	0.7101671437
C	-2.7492817112	0.5065776908	-0.7996731529
C	-1.3671276049	0.3570839566	-0.8270357819
C	-1.0814323776	2.2043299226	0.6621087823
C	-0.4901183244	1.1882775183	-0.1144714338
C	0.9830993024	1.0260397916	-0.1475988306
C	1.7149139425	0.9115862862	-1.3388057476
C	1.7272298853	0.9868657319	1.0475214497
C	3.1130753742	0.8332417402	1.0531277902
C	3.0966048774	0.7574239360	-1.3764031969
C	3.7821437163	0.7158162854	-0.1646225582
O	5.1624779998	0.4703772528	-0.1888885227
C	6.0089134873	1.5490602789	-0.0277985474
O	5.6072372530	2.6930199625	0.0970717998
C	7.4218749066	1.1337878496	-0.0333896221
C	7.8653547376	-0.1291942343	-0.1787717161
C	8.5556645751	2.1329183888	0.1325939171
C	9.3596920061	-0.2416625841	-0.1391115568
N	9.7245160471	1.1951731974	0.0516978571
C	9.9465636492	-0.7746819463	-1.4604144266
C	9.8543720242	-1.0836395784	1.0530448557
C	8.5574795061	2.8357410874	1.5014333443
C	8.6468318436	3.1578551389	-1.0111303779
H	8.6190001149	2.6600327635	-1.9898920785
H	9.5903102533	3.7137889382	-0.9213091129
H	7.8129237283	3.8680512785	-0.9485321879
H	8.4897245069	2.1056224233	2.3192847175
H	7.7077227135	3.5264633947	1.5683151875
H	9.4876377230	3.4099411054	1.6132795380
H	9.5905900892	-0.1870147559	-2.3170000839
H	9.6515958727	-1.8233535088	-1.6013269027
H	11.0433193933	-0.7217340916	-1.4203492686
H	9.4271496618	-0.7208582183	1.9973281665
H	10.9502082668	-1.0261630623	1.1102580003
H	9.5657813063	-2.1341226116	0.9123483009
O	10.9338113571	1.5944081521	0.1479132477
H	-3.3728602540	-0.1818273500	-1.3709914067
F	-0.8563388244	-0.6833844831	-1.5465943098
H	-4.3844945154	1.6605099976	0.0023409828
H	-2.8902343113	3.1772968639	1.3164087677
H	-0.4293060838	2.8774641040	1.2213109413
F	1.0661569674	1.0040331548	-2.5310314848
H	3.6198164064	0.6818020137	-2.3292244335
H	1.1942225865	1.0596106582	1.9965067348
H	3.6682922765	0.7899947622	1.9899269864
H	7.2330974831	-1.0058867656	-0.3128131817

Compound $\mathbf{RCF_3}$

Atom	x	y	z
C	-3.6265896362	2.7751724777	-0.0709257445
C	-2.1330845579	2.5939237119	-0.0382357884
F	-4.1668237625	2.8968218616	1.1736889270
F	-4.2609332135	1.7107451640	-0.6610006036
F	-4.0030530978	3.8789295936	-0.7759106826
C	-1.3571723113	3.0200066177	-1.1245211867
C	-1.5310964751	1.9565570638	1.0536118137
C	-0.1504651221	1.7549844624	1.0667581852
C	0.6096627777	2.1900041817	-0.0181817665
C	0.0224054065	2.8180967255	-1.1173741957
O	1.9807764142	1.9052087491	-0.0212235656
C	2.8614944137	2.9529403990	0.1684699070
O	2.4945150169	4.0931060050	0.3926942998
C	4.2611431469	2.5060018452	0.0757520066
C	4.6650323775	1.2531978859	-0.2081102164
C	5.4263801268	3.4578503874	0.2937980803
C	6.1563174636	1.1034391973	-0.2394993885
H	4.0047489640	0.4091253597	-0.4029875193
N	6.5659739999	2.5017820070	0.0933788041
C	6.6781148496	0.7085002936	-1.6347672360
C	6.6723310591	0.1230852420	0.8313482451
C	5.4903329076	4.0356192115	1.7179109319
C	5.5113055516	4.5779164775	-0.7575983701
O	7.7880561464	2.8618366155	0.1812920747
H	5.4487775411	4.1692921130	-1.7752881214
H	6.4677472054	5.1068558291	-0.6443170682
H	4.6940268771	5.2949812135	-0.6104329019
H	5.4220251401	3.2385640936	2.4707063420
H	4.6670060374	4.7440999662	1.8726945363
H	6.4422832396	4.5684346538	1.8493486386
H	6.3036176282	1.3952956625	-2.4054089820
H	6.3524137902	-0.3118729267	-1.8783147224
H	7.7766000505	0.7349061349	-1.6347602335
H	6.2942204505	0.3903539936	1.8271083402
H	7.7707461786	0.1446620274	0.8473218753
H	6.3459722044	-0.8973643343	0.5888990163
H	-1.8256887077	3.5112622070	-1.9772755909
H	0.6378484467	3.1412439854	-1.9570092103
H	-2.1333353386	1.6211641696	1.8978208708
H	0.3363968107	1.2647096758	1.9099301997

S3.2 DFT analysis of the dihedral angle ξ in $\mathbf{RF_2}$ and RFF'

To study the flexibility of the model systems $\mathbf{RF_2}$ and **RFF'**, relaxed surface scans of dihedral angle ξ, and *g*- and $A(^{14}N)$-tensor frames calculation were performed at UKS, B3LYP/def2-tzvp level of theory. The resulting energies and Boltzmann weights are shown in Figures S8 and S9.

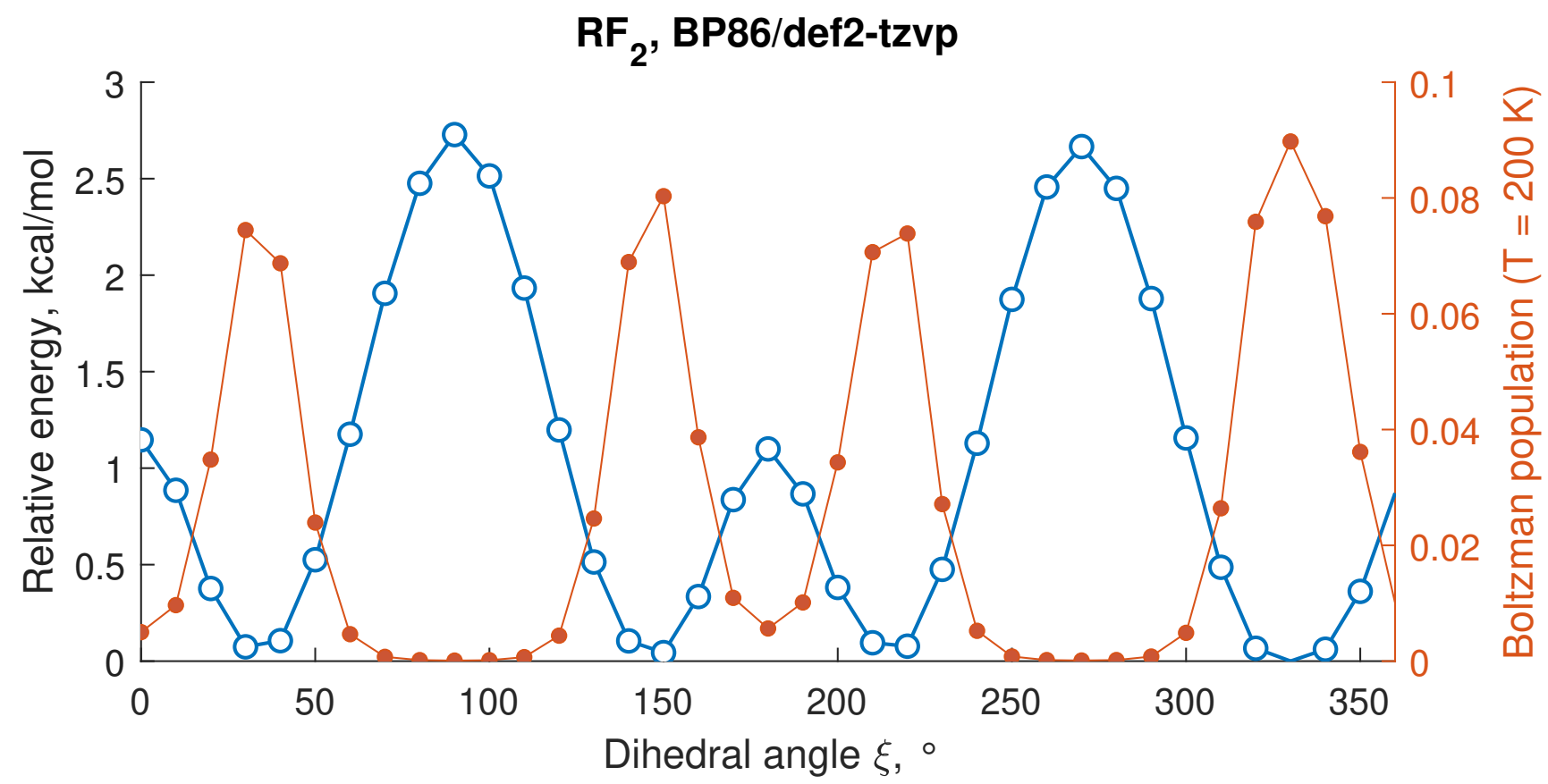

Figure S8: **Dihedral angle scan for $\mathbf{RF_2}$.** Rotational relaxed energy scan of compound $\mathbf{RF_2}$ (blue line) and corresponding Boltzmann weights ($T = 200$ K). ξ is the dihedral angle between two benzene rings.

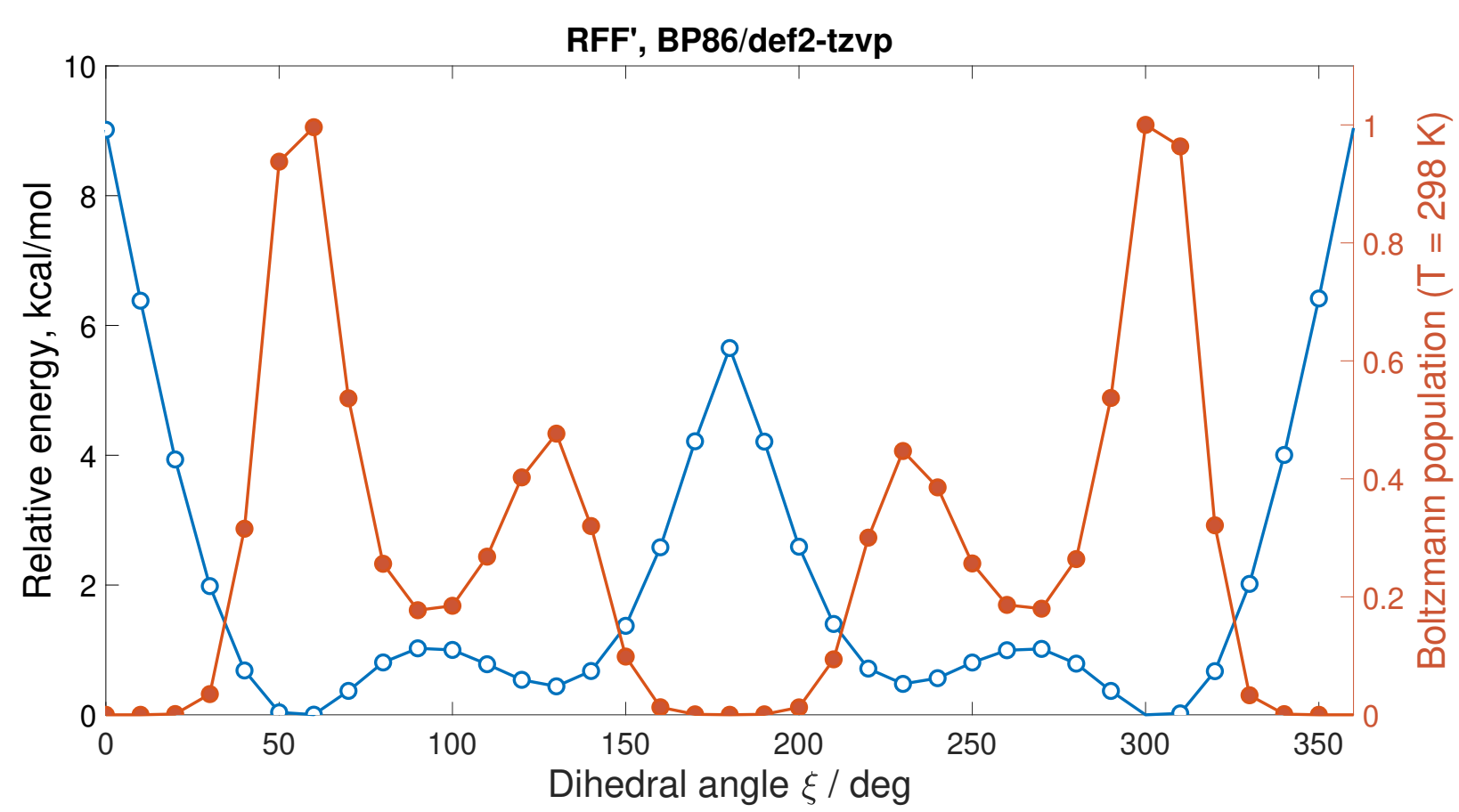

Figure S9: **Dihedral angle scan for RFF'.** Rotational relaxed energy scan of **RFF'** (blue line) and corresponding Boltzmann weights ($T = 298$ K). ξ is the dihedral angle between two benzene rings.

The distribution of ξ has a strong influence on the ^{19}F-^{19}F distance distribution in **RFF'**. DFT calculations reveal two energy minima at $\xi \approx 60°$ and $\xi \approx 130°$, with an energy difference of 1.8 kcal/mol. The minima represent a compromise between minimizing the steric hindrance of the ortho substituents in a fully coplanar arrangement of the two benzene rings and avoiding the complete loss of conjugation that occurs in a perpendicular biphenyl conformation. A dihedral angle of $\xi \approx 60°$ corresponds to a short ^{19}F-^{19}F distance, whereas an angle of $\xi \approx 130°$ corresponds to a longer one. Corresponding structures of **RFF'** are shown in Figure S10.

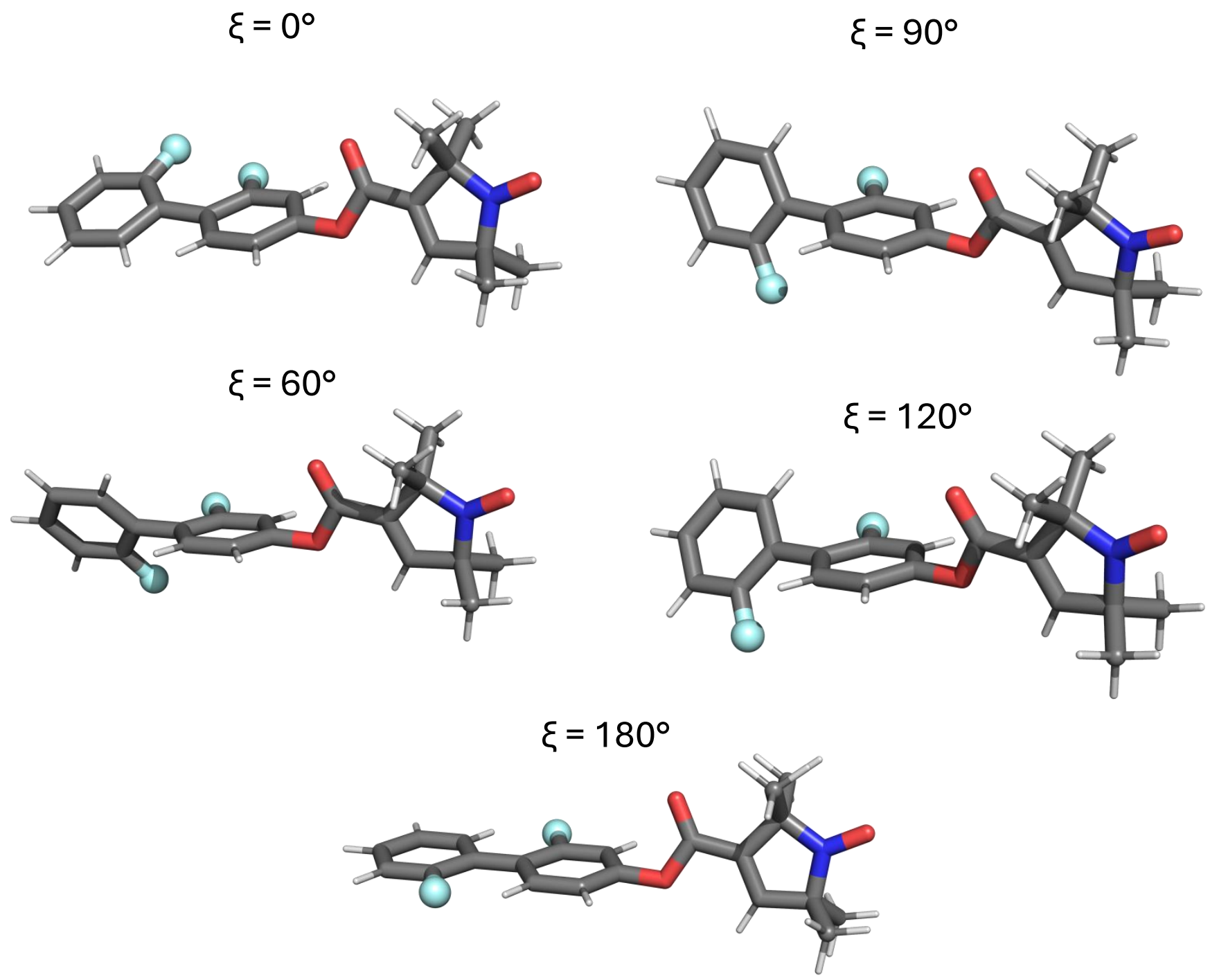

Figure S10: **DFT-optimized structures of RFF'.** Different dihedral angles ξ set as a geometric constraint in ORCA input are presented.

The Boltzmann distribution predicts a higher population of the short-distance rotamer, in agreement with the NEEM experiments presented in the main text (Nitroxide-^{19}F and ^{19}F-^{19}F distance distributions in **RFF'**). The optimized geometries obtained from the DFT calculations were used to generate distributions of both inter-nuclear and nitroxide–nuclear distances. To extract these distance distributions, 36 Gaussians, each centered on the individual inter-spin distances of the conformers, were summed and weighted by their Boltzmann populations, yielding the final distance distribution shown in Figure 7 (main text). Each Gaussian function had a variance of $\sigma = 0.12$ Å for inter-nuclear distances and $\sigma = 0.20$ Å for electron–nuclear distances

S4 EPR characterization

S4.1 EPR spectrum and observer positions

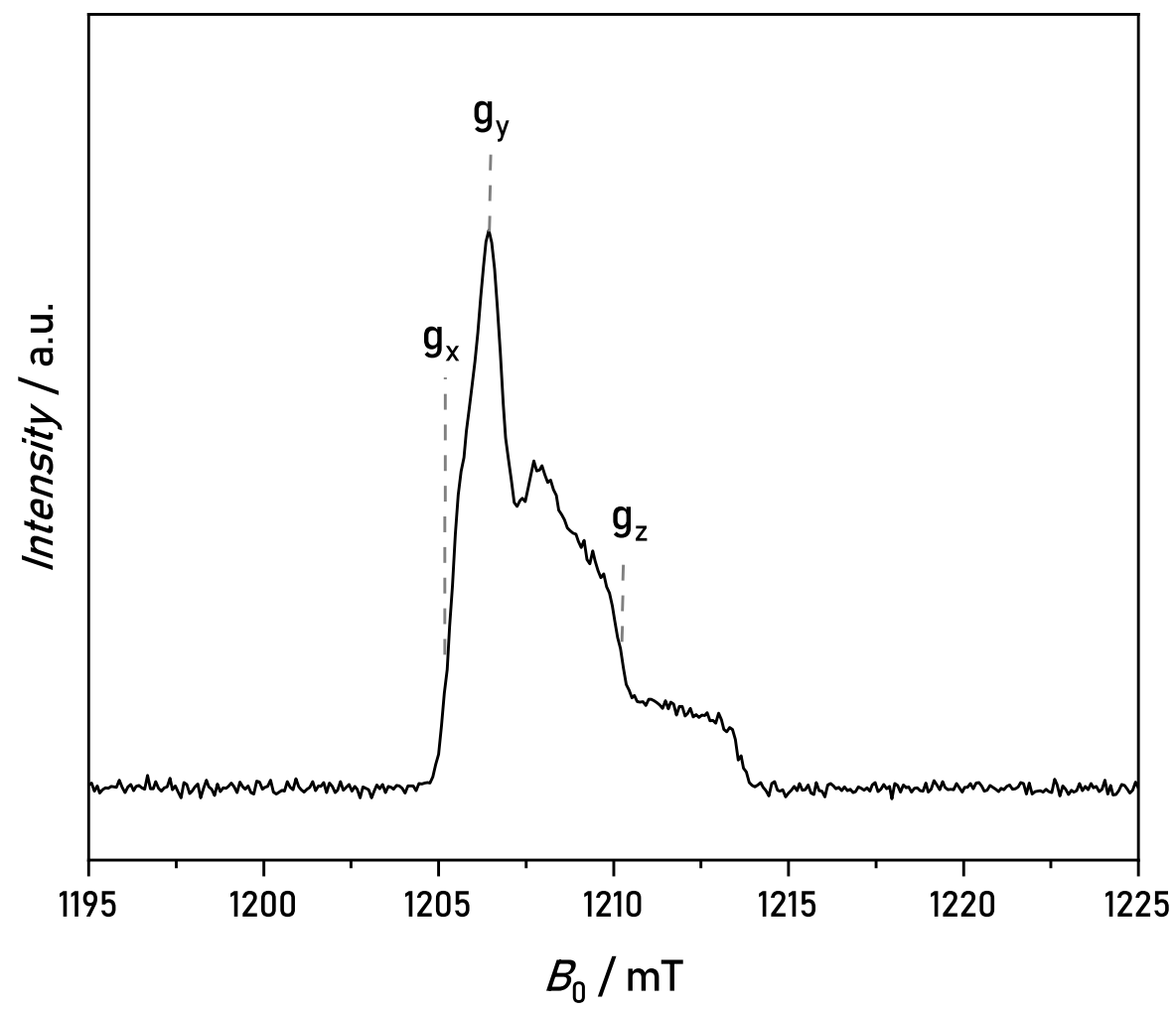

Figure S11: **Echo-detected field-swept EPR spectrum.** The measurement is performed at Q band, 50 K for model compound $\mathbf{RCF_3}$ with $\tau = 450$ ns. Three different observer positions are marked.

S4.2 Relaxation measurements

Relaxation characterization for the electron spin included the following experiments:

- Hahn echo decay: $\pi/2 - \tau - \pi - \tau -$ echo (τ is varied);
- Stimulated echo decay: $\pi/2 - \tau - \pi/2 - T - \pi/2 - \tau -$ echo (T is fixed, τ is varied);
- Inversion recovery: $\pi - t - \pi/2 - \tau - \pi - \tau -$ echo (t is varied).

The length of pulses was same in all experiments: $t_{\pi/2} = 12$ ns, $t_\pi = 24$ ns. The data were fitted with the following model functions:

- Hahn echo decay: sum of stretched exponentials (*78*) $c_1 \exp(-(2\tau/\tau_{\text{fast}})^{\xi_1}) + c_2 \exp(-(2\tau/\tau_c)^{\xi_2})$, where we fixed $\xi_1 = 1$ to stabilize the fitting. The first term phenomenologically describes the tunneling contribution from the vicinal methyl groups (*77-78*). The second term with characteristic time T_c describes the general electron spin decoherence.
- Stimulated echo decay: monoexponential $a \exp(-\tau/T_m)$, where T_m is parameterized by T.
- Inversion recovery: stretched exponential $a - b \exp(-(t/T_{1,e})^{\xi})$, where $T_{1,e}$ is spin-lattice relaxation time.

Table S4: **Relaxation characterization of compounds $\mathbf{RF_2}$, RFF', $\mathbf{RCF_3}$.** The phase memory time T_m is reported for $T = 200\,\mu s$. The default measurement temperature is 50 K, unless stated otherwise.

Compound	Position	$[\tau_{\text{fast}}/\mu s, T_c/\mu s, \xi_2, c_1, c_2]$	$[T_{1,e}/\text{ms}, \xi]$	$T_m/\mu s (T = 200\,\mu s)$
$\mathbf{RF_2}$	g_x	–	[16.4, 0.84] (20 K)	
		[2.7, 27.5, 1.35, 0.17, 0.83] (50 K)	[1.2, 0.90] (50 K)	6.46
	g_y	–	[20.5, 0.81] (20 K)	
		[2.9, 19.9, 1.30, 0.23, 0.77] (50 K)	[1.6, 0.83] (50 K)	5.33
	g_z	–	[19.2, 0.80] (20 K)	
		[2.7, 24.8, 1.35, 0.24, 0.76] (50 K)	[2.1, 0.79] (50 K)	5.41
**RFF'**	g_x	[2.1, 24.8, 1.67, 0.19, 0.81]	[1.22, 0.85]	4.62
	g_y	[1.8, 22.3, 1.58, 0.25, 0.75]	[1.57, 0.80]	4.47
	g_z	[1.8, 22.1, 1.55, 0.25, 0.75]	[2.10, 0.79]	4.12
$\mathbf{RCF_3}$	g_x	–	[1.21, 0.85]	3.46
	g_y	–	[1.50, 0.78]	3.50
	g_z	–	[1.72, 0.75]	3.43

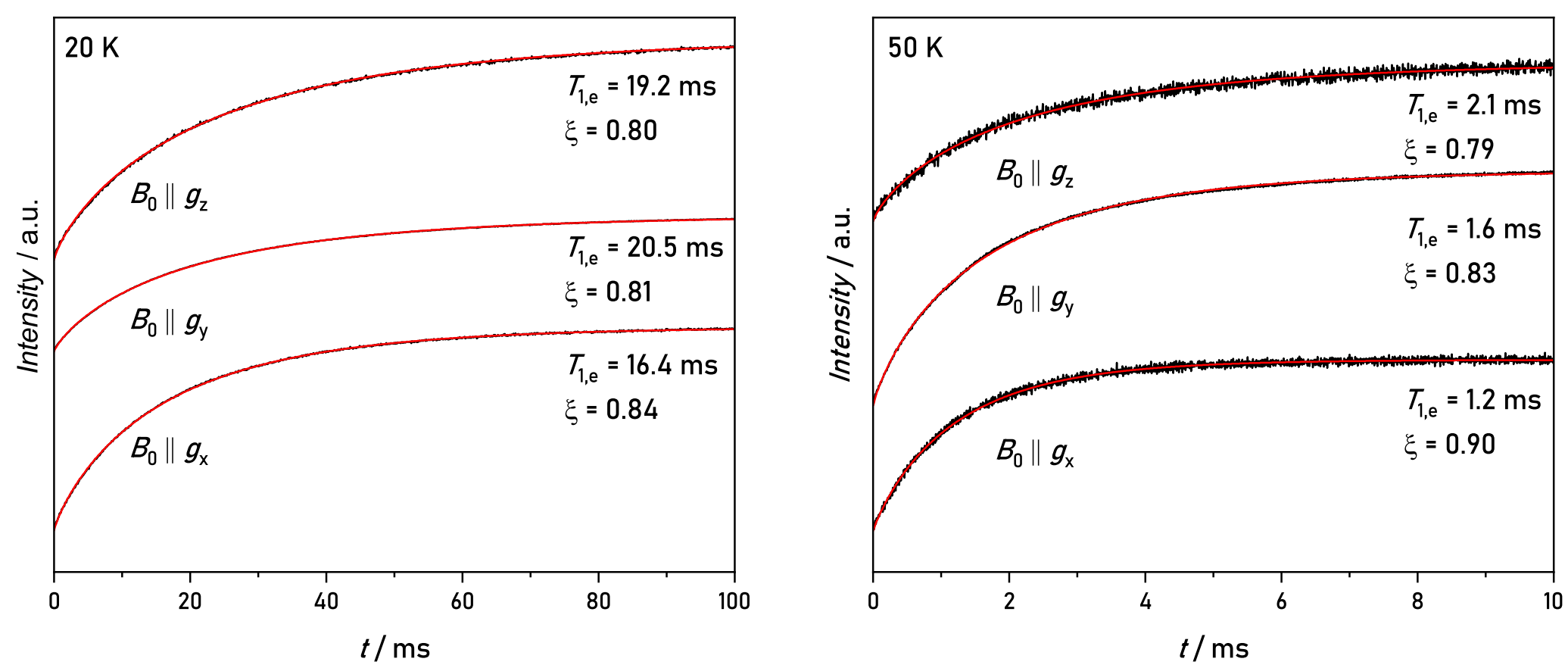

Figure S12: **Inversion recovery experiments.** The sequence $\pi - t - \pi/2 - \tau - \pi - \tau -$ echo was performed at 20 K (left panel) and 50 K (right panel) with compound $\mathbf{RF_2}$ at three different observer positions in the EPR line. The traces were fitted with a stretched exponential function $a - b\exp(-(t/T_{1,e})^{\xi})$.

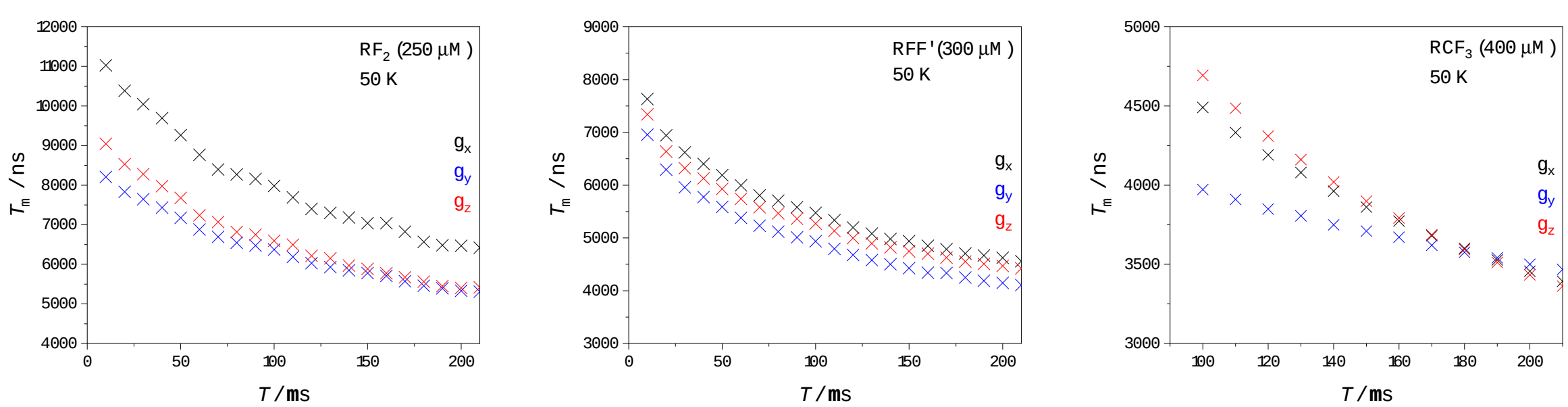

Figure S13: **Phase memory time of the nitroxide model compounds.** Measurements were performed at 50 K with variable time increment T. T_m was obtained by fitting the stimulated echo decay with a monoexponential function.

S5 Simulation

Coordinate-based simulation of NEEM data was performed as follows. Atomic coordinates were shifted to ensure the electron spin (approximated as the center of the NO bond) is at the origin. A unit origin-centered spherical grid was generated with the `sphgrid` function in EasySpin. The grid's symmetry is `'C1'`, and the number of nodes was 3366. The orientation of the external magnetic field was sampled over the grid as unit vectors $\mathbf{n}$. This direction was used as a spin quantisation axis (z-axis) for both the electron and nuclei. The secular coupling constant of any interaction represented by a tensor $\underline{K}$ in the new frame is computed as

$$K_{zz} = \mathbf{n}^{\mathrm{T}}\underline{K}\mathbf{n}\,. \tag{S68}$$

For tensors of axial symmetry, i.e. $\underline{K}^{\mathrm{PAS}} = \{K, K, -2K\}$, e.g. HFC and NDC tensors, this formula can be specified

$$K_{zz} = K(1 - 3\cos^2\theta) \tag{S69}$$

where θ is the angle between the tensor's symmetry axis and $\mathbf{n}$. For HFC, $K = -2\pi T = \mu_0/(4\pi\hbar)\cdot g_e\mu_{\mathrm{B}}g_n\mu_N/r^3$ and θ is denoted as θ_1 in Figure S14. For NDC, $K = 2\pi\nu_\perp = \mu_0/(4\pi\hbar)\cdot g_1g_2\mu_N^2/r_{1,2}^3$ and θ is denoted as $\theta_{1,2}$ in Figure S14.

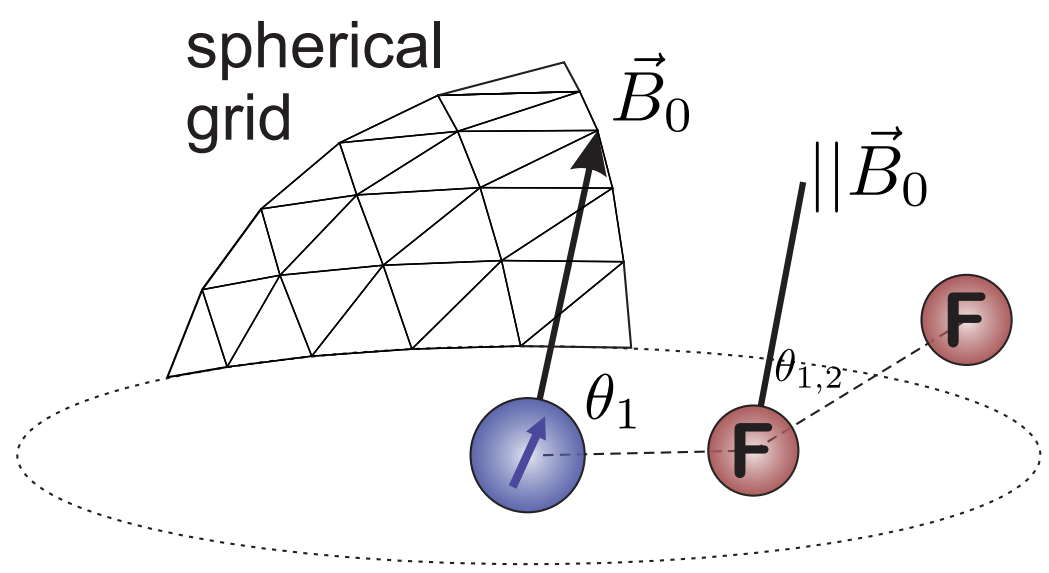

Figure S14: **Scheme of coordinate-based simulations of NEEM.** Nodes of the spherical grid (only a fragment is shown) served for sampling of the magnetic field's directions, $\mathbf{n}$. θ_1 is the dipolar angle for fluorine 1, $\theta_{1,2}$ is the fluorine-fluorine dipolar angle.

The computed coupling constants were either plugged into the analytical formulae for NEEM or used to set up the spin Hamiltonian for numeric spin dynamics computation (see Section S15).

Below, we list the Euler angles α and β (ZY'Z" convention) of the nitroxide-^{19}F HFC and ^{19}F-^{19}F NDC tensors for both rotamers of $\mathbf{RF_2}$ with respect to the g-frame. Angle γ is not used due to the axial symmetry of both tensors. Additionally, we specify the tensors' principal values in linear frequency units.

Rotamer		HFC	NDC
1	F1:	$[10.5°, -85.4°, 0°]$<br>$[27.1, 27.1, -54.2]$ kHz	$[-64.4°, -51.9°, 0°]$<br>$[1.01, 1.01, -2.02]$ kHz
	F2:	$[26.0°, -97.6°, 0°]$<br>$[29.6, 29.6, -59.2]$ kHz	
2	F1:	$[14.5°, -100.6°, 0°]$<br>$[28.1, 28.1, -56.2]$ kHz	$[-72.7°, -159.0°, 0°]$<br>$[1.01, 1.01, -2.02]$ kHz
	F2:	$[21.6°, -82.2°, 0°]$<br>$[28.6, 28.6, -57.2]$ kHz	

To simulate the orientation-selective experiments, EPR resonance fields were calculated for each orientation of the external magnetic field $\mathbf{n}$

$$B_{\mathrm{res}}(\mathbf{n}, m_I) = \frac{h\nu_{\mathrm{mw}} + m_I a_{\mathrm{eff}}(^{14}\mathrm{N})}{g_{\mathrm{eff}}\mu_B} \tag{S70}$$

where $m_I = -1, 0, +1$ (magnetic quantum numbers of ^{14}N) and $g_{\mathrm{eff}} = \mathbf{n}^{\mathrm{T}}\underline{g}\mathbf{n}$, $A_{\mathrm{eff}}(^{14}\mathrm{N}) = \mathbf{n}^{\mathrm{T}}\underline{A}(^{14}\mathrm{N})\mathbf{n}$. The principal values of g- and A-tensors are $[2.00211, 2.00610, 2.00886]$, respectively, $[0.55, 0.39, 3.4]$ mT (*29*). To simulate the effect of selective pulses, we computed in Section S5.1 the excitation profile of the MW observer

sequence (stimulated echo). After that, ENDOR signals from all orientations were summed with a weight function that approximates this profile

$$w(\mathbf{n}, m_I) = \exp\left(-\frac{(B_{\text{res}}(\mathbf{n}, m_I) - B_0)^2}{2s^2}\right) \tag{S71}$$

where B_0 is the experimental field position and $s = 0.6\,\text{mT}$ is a parameter determined in the additional simulation (see Section S5.1).

Simulation of NEEM data with $\mathbf{RCF_3}$ is described in Section S15.

S5.1 Excitation profile in the electron stimulated echo experiment

The excitation profile in the stimulated echo experiment was simulated as follows. We introduced a resonance offset $\Omega := \omega_e - \omega_{\text{mw}}$, hence, the spin Hamiltonians in rotating frame were

$$\hat{H}_{\text{free}} = \Omega \hat{S}_z \tag{S72}$$

for free evolution and

$$\hat{H}_{\text{p}} = \omega_1 \hat{S}_x + \Omega \hat{S}_z \tag{S73}$$

for the real pulse, where $\omega_1 = (\pi/2)/t_{\pi/2}$ is the pulse Rabi frequency. We propagated the reduced equilibrium density matrix $\hat{\sigma}_0 = -\hat{S}_z$ through a stimulated echo sequence $\pi/2 - \tau - \pi/2 - T - \pi/2 - \tau -$ det and detected the echo intensity as a function of Ω. Moreover, we simulated the coherence pathway $+1 \to 0 \to -1$ to reject interference from non-relevant pathways (Fig. S15a). The computation can be represented by the chain

$$-\hat{S}_z \xrightarrow{\hat{H}_{\text{p}} t_{\pi/2}} \xrightarrow{\text{proj.}\hat{S}^+} \xrightarrow{\hat{H}_{\text{free}}\tau} \xrightarrow{\hat{H}_{\text{p}} t_{\pi/2}} \xrightarrow{\text{proj.}\hat{S}_z} \xrightarrow{\hat{H}_{\text{free}}T} \xrightarrow{\hat{H}_{\text{p}} t_{\pi/2}} \xrightarrow{\text{proj.}\hat{S}^-} \xrightarrow{\hat{H}_{\text{free}}\tau} \sigma \tag{S74}$$

followed by signal detection $V = \text{tr}(\hat{S}^+\sigma)$. In this scheme, projections allow to select a specific coherence pathway. A projection on operator $\hat{X}$ is defined as

$$\sigma \xrightarrow{\text{proj.}\hat{X}} \sigma_X = \frac{\text{tr}(\hat{X}^\dagger \sigma)}{\text{tr}(\hat{X}^\dagger \hat{X})} \hat{X}\,. \tag{S75}$$

The simulation parameters were $t_{\pi/2} = 12\,\text{ns}$, $\tau = 3\,\mu\text{s}$, $\Omega \in [-1000, 1000]\text{Mrad}\,\text{s}^{-1}$. The result is given in Figure S15b. The profile can be approximated as a Gaussian shape with standard deviation $s = 0.60\,\text{mT} = 16.8\,\text{MHz}$. This width parameter scales linearly with the electron Rabi frequency.

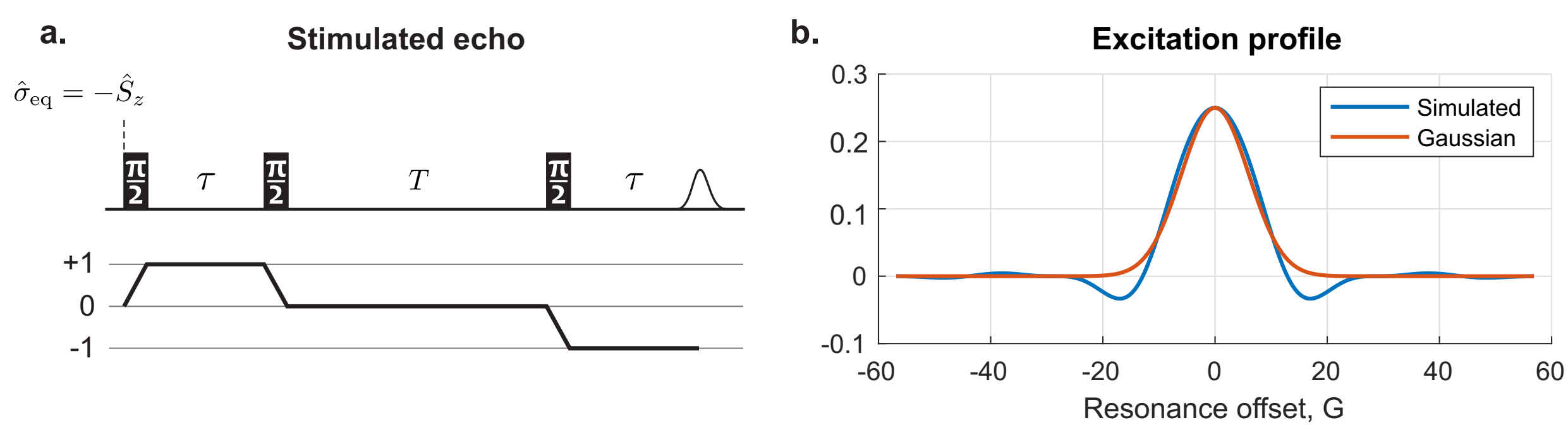

Figure S15: **Excitation profile in stimulated echo.** a. The stimulated echo sequence with three rectangular $\pi/2$ pulses and the coherence pathway chosen for simulation. b. Effective excitation profile in stimulated echo experiment computed for rectangular pulses with $t_{\pi/2} = 12\,\text{ns}$. Blue line is the exact result, and orange line is a Gaussian approximation with standard deviation $s = 0.60\,\text{mT}$. The conversion coefficient between $\text{Mrad}\,\text{s}^{-1}$ and G is $2\pi \cdot 2.8\,\text{Mrad/s/G}$

S5.2 Simulated NEEM with different relative orientation of HFC and NDC tensors

By performing a numerical simulation, we found that the powder-averaged NEEM signal with two nuclei has a slightly different shape depending on the relative orientation of hyperfine (HFC) and nuclear dipolar (NDC) tensors. This is expected because each nuclear signal is additionally scaled with the blind spot function that depends on A and τ. We assume that the two hyperfine tensors are almost collinear, which is valid if the electron-nuclear distances are much larger than the inter-nuclear distance. If, moreover, both tensors are of axial symmetry, their orientation is quantified by only one Euler angle Θ as shown in Figure S16. This Figure also contains simulated powder-averaged NEEM signals for Θ values from 0° to 90°. Our case of model compound $\mathbf{RF_2}$ with the experimental sum spectrum displayed in Figure 4B (main text), is close to $\Theta = 90°$.

Next, we comment on some predicted trends. The NEEM spectrum at $\Theta = 0°$ visually resembles a PDHS spectrum. In fact, angle $\Theta = 0°$ means that the three spins are placed on a single line; hence, HF and ND tensors become coaxial. Therefore, nuclear couplings with $\omega_{1,2} = 0$ correspond to the orientations where $A_1 = A_2 = 0$. Consequently, they are not detected, since in these orientations the blind spot function is also zero. This manifests as the zero intensity at $f = 0$. In other configurations, $\omega_{1,2} = 0$ does not vanish, and a non-zero intensity at $f = 0$ is observed.

Similar blind spot effects, marked with arrows in Figure S16, are prominent at $\Theta = 90° - \Theta_M \approx 36°$ and $\Theta = \Theta_M \approx 54°$ (Θ_M is magic angle). In the first case, the hyperfine blind spot function suppresses $f = \nu_\perp$ (i.e. "horns" of the Pake pattern). The second case reveals suppression of the pattern shoulders, i.e. $f = \nu_\parallel = 2\nu_\perp$.

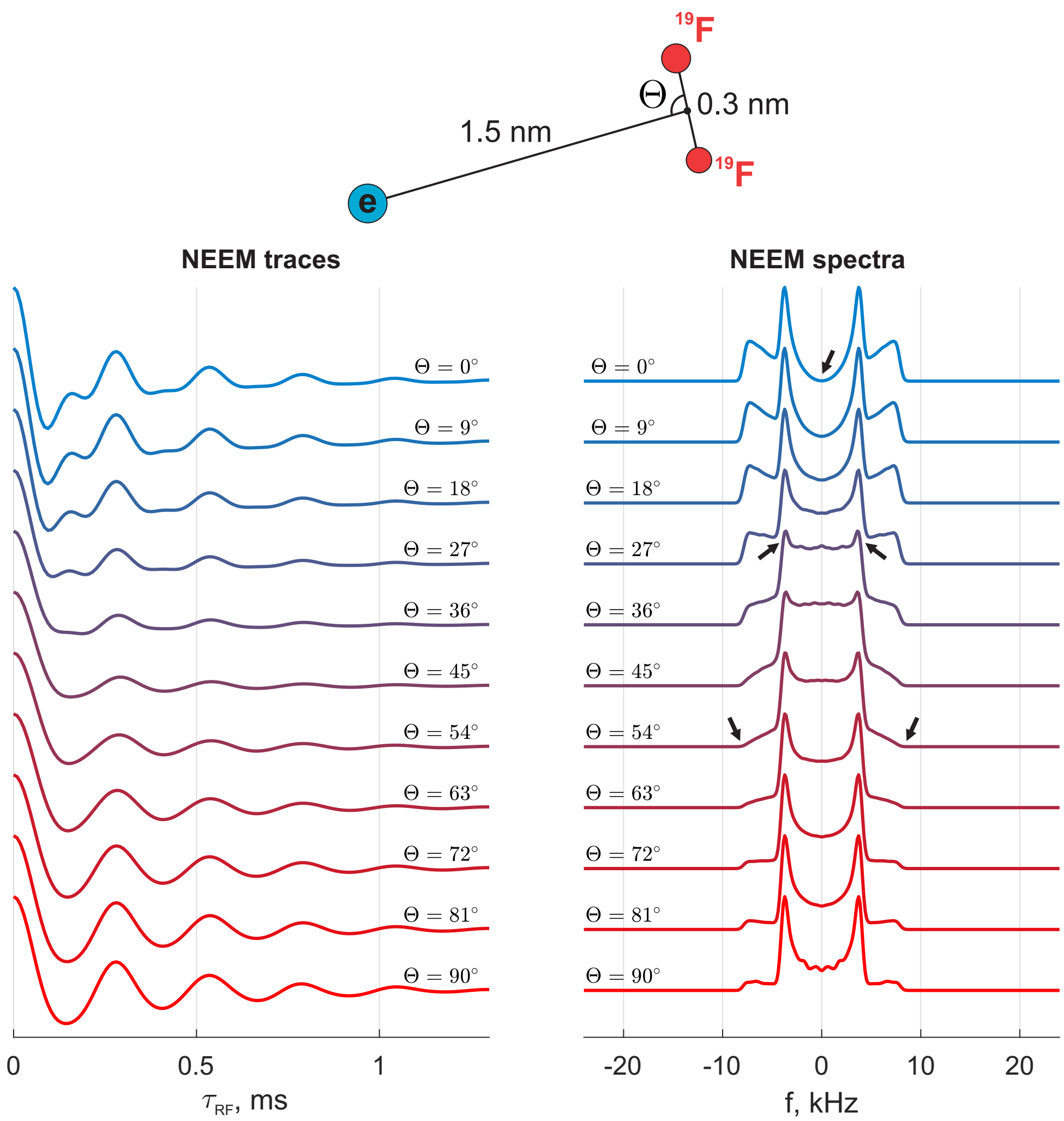

Figure S16: **Dependence of NEEM on relative orientation of HF and ND tensors.** Simulated powder-averaged NEEM traces (left) and their Fourier transforms (right) computed for a pair of ^{19}F nuclei. The simulation was repeated for different angles Θ (see top panel), which defines the angle between the hyperfine and nuclear dipolar symmetry axes. The spectra resemble dipolar Pake patterns, and the pronounced deviations, marked by arrows, are explained in the main text. Overall, this demonstrates that NEEM data contain information on the nuclear pair orientation relative to the electron. Simulation was performed with analytical formula (18) in the main text with the following parameters parameters: $\tau = 3\,\mu s$, electron-^{19}F distance 1.5 nm, ^{19}F-^{19}F distance 0.3 nm ($\nu_\perp = 3.94$ kHz).

S6 Background signal in NEEM experiment

In the NEEM data, a monoexponential background was observed and subtracted prior to the Fourier transform. The experimental NEEM traces of all measurements presented in the main text are shown in Figure S17.

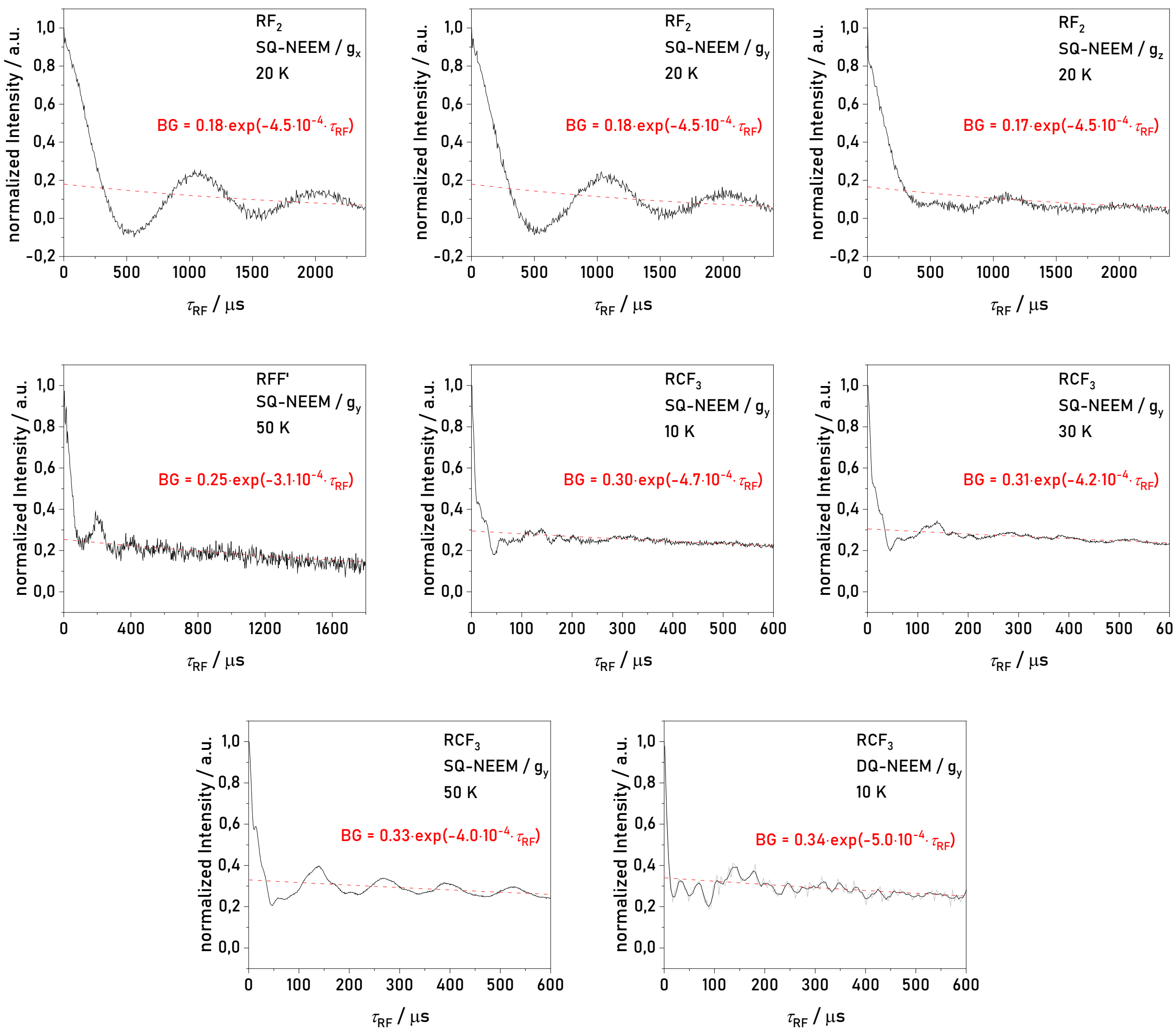

Figure S17: **Raw NEEM traces of all measurements presented in the main text.** Experimental parameters for the respective measurements are given in Figures 4, 7, and 8 (main text). The grey trace for DQ-NEEM of $\mathbf{RCF_3}$ is the raw data, and the black trace was smoothed using a Savitzky-Golay filter (window size: 6 points). The subtracted exponential backgrounds are shown as red dashed lines with their respective formulas.

S7 Nuclear echo decay at 20 K for a monofluorinated compound

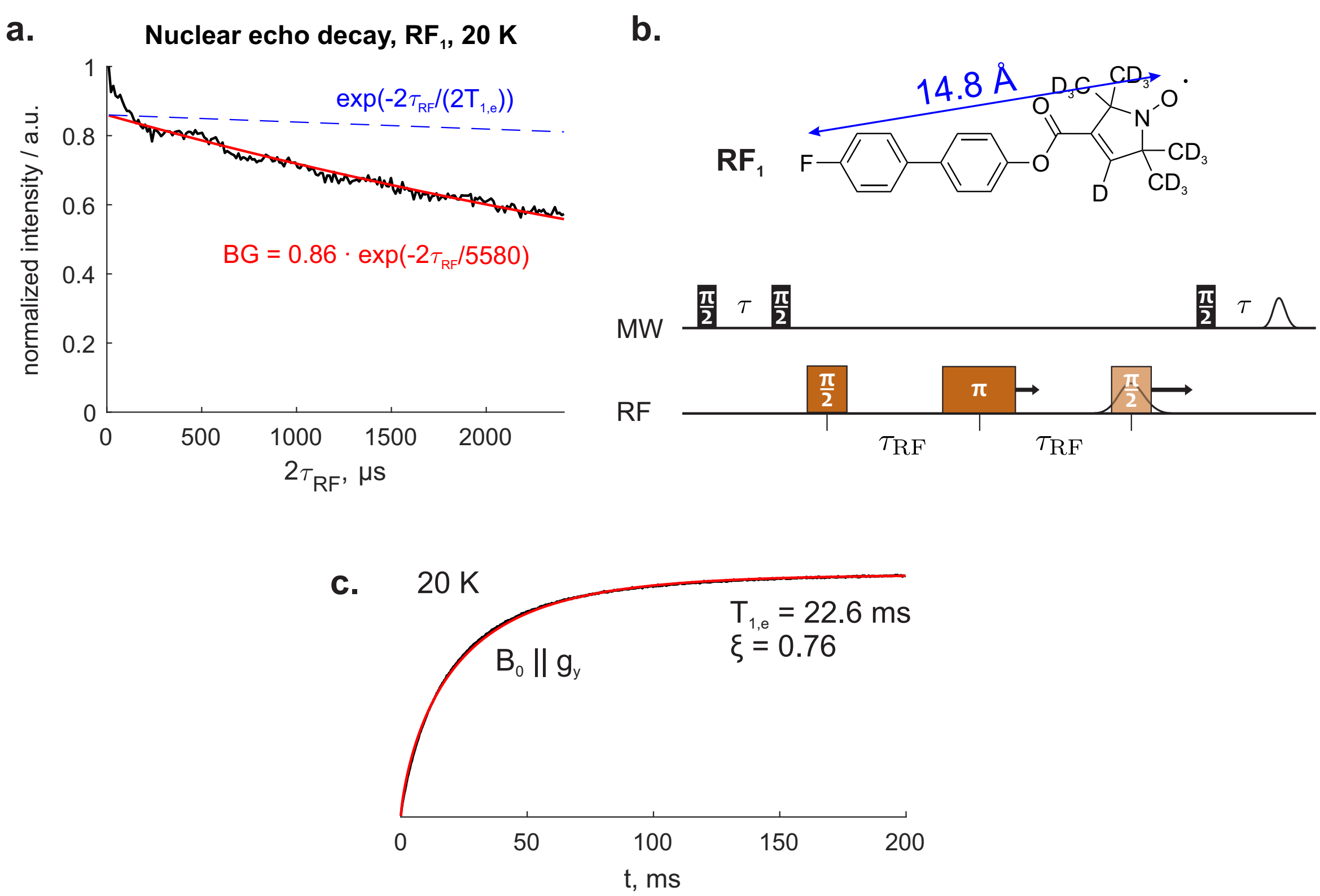

Figure S18: **Singly-fluorinated compound.** a. Nuclear echo decay in singly-fluorinated compound $\mathbf{RF_1}$ at 20 K. Black line is an experimental trace, red solid line is a monoexponential fit, blue dashed line is the predicted decay in the model $T_{2,n} = 2T_{1,e}$. Experimental parameters: 12 ns MW π/2 pulses, 4 µs RF π/2 pulses, τ = 3 µs, g_y observer position, τ_{RF} was incremented in 6 µs steps. b. Top: Chemical structure of the compound $\mathbf{RF_1}$, which features nitroxide-^{19}F distance close to that in $\mathbf{RF_2}$. This compound was previously characterized in Ref. (*31*). Bottom: Pulse sequence used to measure the nuclear echo decay. It is equivalent to SQ-NEEM in this work. c. Inversion recovery trace of $\mathbf{RF_1}$ measured at g_y observer position at 20 K. The red trace is the fit with a stretched exponential function $a - b\exp(-(t/T_{1,e})^{\xi})$. The fitted $T_{1,e}$ was used to calculate the blue line in panel (a).

S8 NEEM of RF_2 at 50 K with low sample concentration (250 µM and 10 µM)

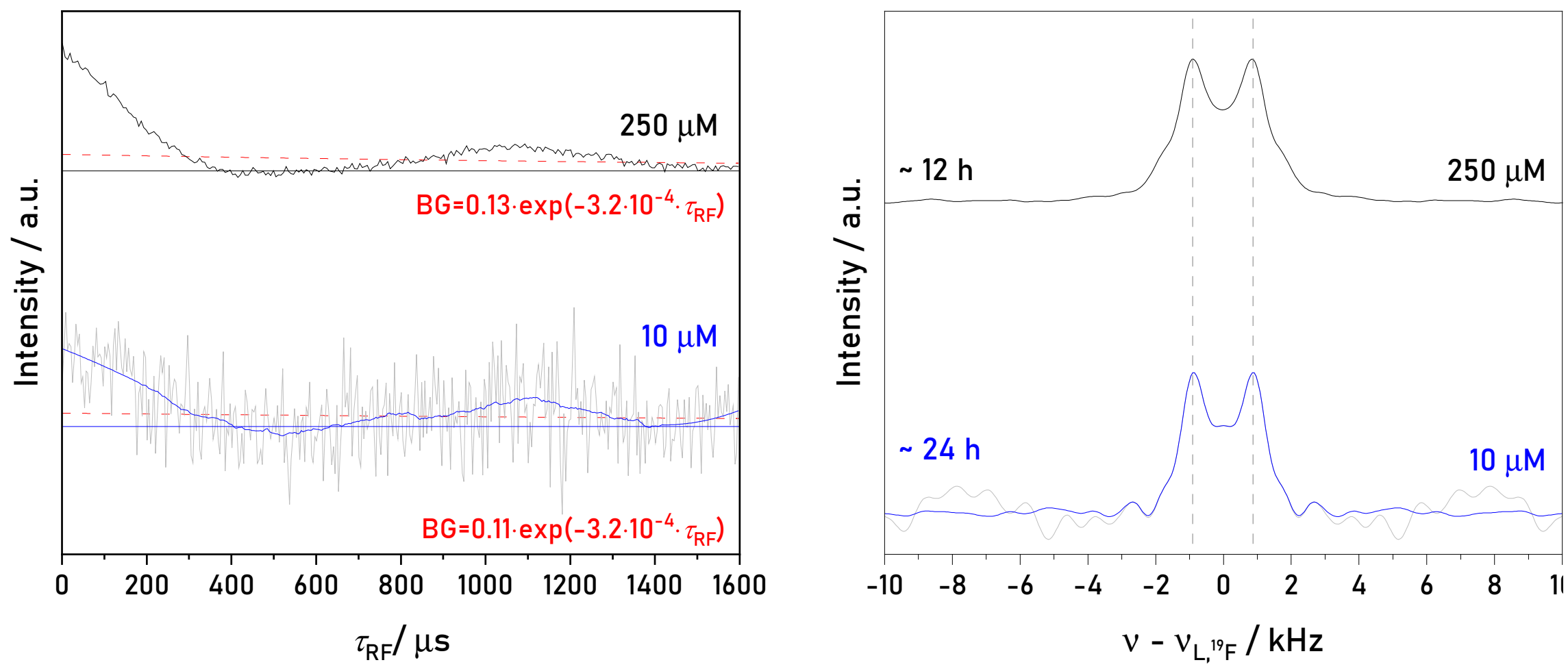

Figure S19: **Concentration series of RF_2.** Left: SQ-NEEM traces of $\mathbf{RF_2}$ measured at 50 K with sample concentrations of 250 µM (black) and 10 µM (blue) with 12 ns MW π/2 pulses, 4 µs RF π/2 pulses, $\tau = 3$ µs, 6 ms (10 µM) and 8 ms (250 µM) shot-repetition time (srt) and 10 shots-per-point (spp) at the g_y observer position of the EPR spectrum. The grey trace is the raw data, and the blue trace was smoothed using a Savitzky-Golay filter (window size: 90 points). Zero lines of both traces are indicated. Exponential background subtracted prior to the Fourier transform is indicated as dashed red lines. Right: Fourier transform of the corresponding (background-corrected) NEEM traces.

S9 Quadrature detection of nuclear echo in NEEM and PDHS with RF offset

Theoretical analysis of the NEEM and PDHS experiments predicts that the nuclear resonance offsets are refocused at the position of the nuclear echo detection. We explicitly tested this with a model compound $\mathbf{RF_2}$. To this end, we performed these experiments at the center of the ^{19}F-ENDOR spectrum and at one (SQ-NEEM) or several (SQ-PDHS) offset frequencies (Figure S20a and Figure S21a). We acquired both real ($V_{\rm re}$) and imaginary ($V_{\rm im}$) components of the nuclear echo. The latter was done by shifting the phase of the read-out RF $\pi/2$ pulse by 90°. We also computed the real Fourier transform $\mathcal{F}[V_{\rm re}]$ and the complex Fourier transform $\mathcal{F}[V_{\rm re} + iV_{\rm im}]$ for each dataset. The results are presented in Figures S20(b,c) and S21(b,c).

The resulting NEEM and PDHS traces and spectra are close. The shape match of the real FT spectra is within the experimental error. We observe a weak asymmetry in complex FT PDHS spectra for $\Delta\nu = 20\,\text{kHz}$, however, without a peak shift. While the physical reason for the asymmetry is likely the limited RF excitation bandwidth, these data show that small frequency offsets are tolerated in experiments with nuclear echo detection.

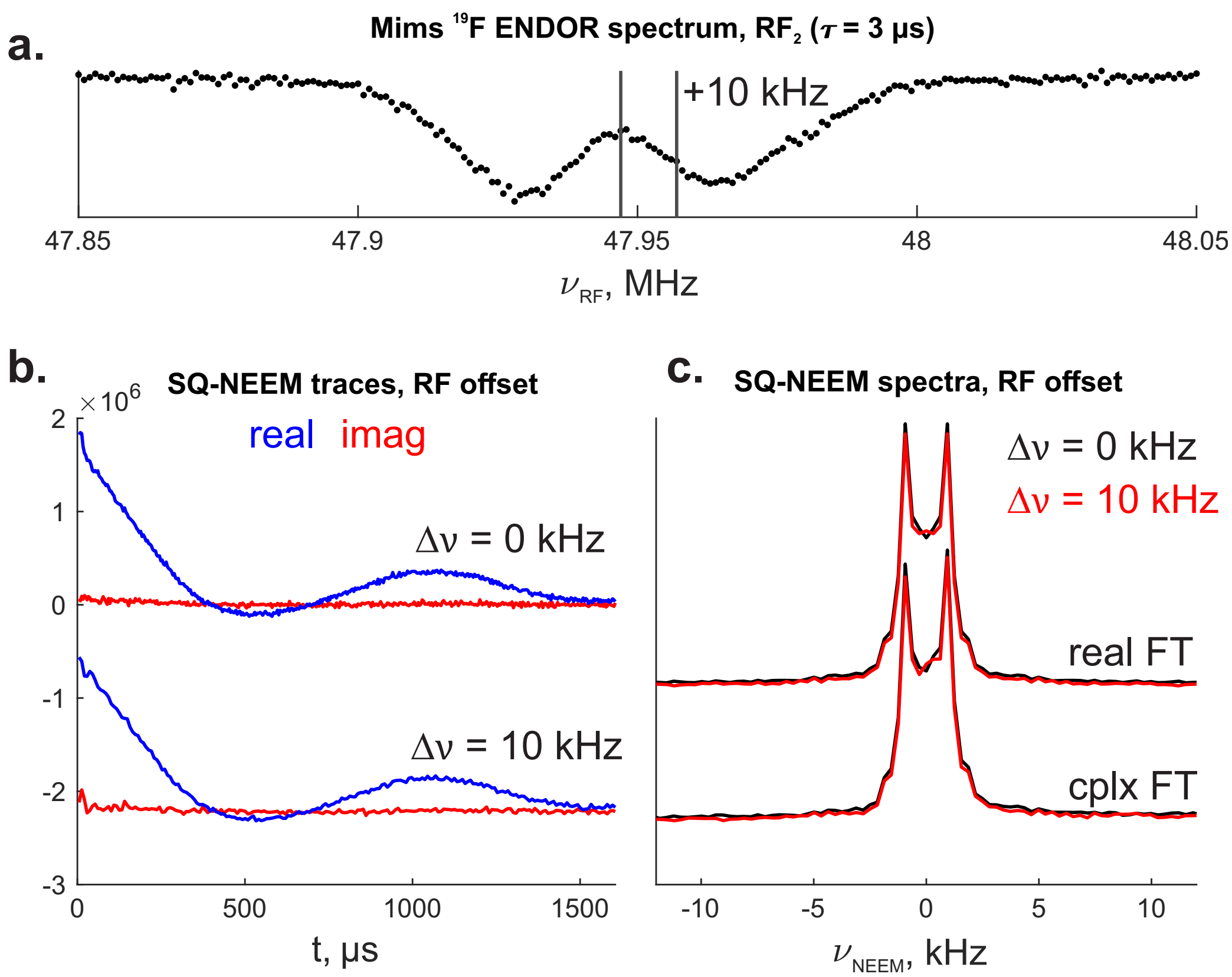

Figure S20: **Quadrature nuclear echo detection in NEEM with model compound $\mathbf{RF_2}$.** a. Mims FD ENDOR spectrum acquired with 12 ns MW pulses, 50 µs RF pulse, $\tau = 3\,\mu$s. Vertical lines mark the center of the spectrum (47.947 MHz) and the offset frequency by 10 kHz. b. NEEM traces (real and imaginary components of the nuclear echo) acquired at the center frequency (top) and at the offset frequency (bottom). c. Comparison of real (top) and complex (bottom) Fourier spectra of NEEM traces acquired at the center (black) and the offset (red) frequency. All measurements were performed at 50 K.

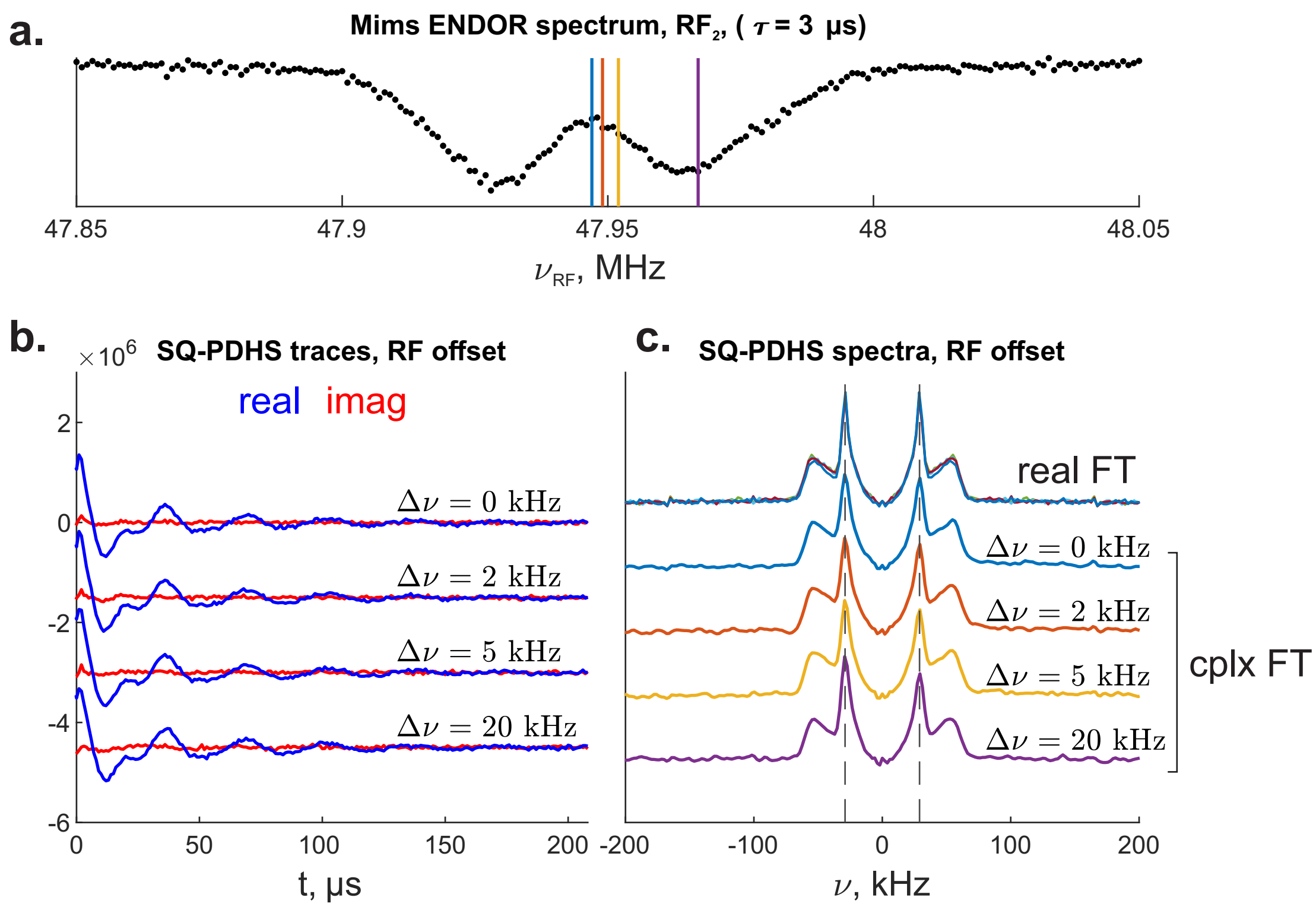

Figure S21: **Quadrature nuclear echo detection in PDHS with model compound $\mathbf{RF_2}$.** a. Mims FD ENDOR spectrum acquired with 12 ns MW pulses, 50 µs RF pulse, $\tau = 3$ µs. Vertical lines mark the center of the spectrum (47.947 MHz) and the offset frequencies by 2, 5 and 20 kHz. b. SQ-PDHS traces (real and imaginary components of the nuclear echo) acquired at the center frequency and at the offset frequencies. c. Comparison of real (top) and complex Fourier spectra of SQ-PDHS traces acquired at the center and the offset frequencies. All measurements were performed at 50 K.

S10 Ratio of SQ- and DQ-PDHS intensities with $\mathbf{RF_2}$

Eq. (21) in the main text predicts the τ dependence of SQ- and DQ- contributions in PDHS as follows:

$$V_{\mathrm{SQ}}(\tau,0) = \left(\sin^2\frac{A_1\tau}{2}\cos^2\frac{A_2\tau}{2} + \cos^2\frac{A_1\tau}{2}\sin^2\frac{A_2\tau}{2}\right)\cos\omega_{1,2}\tau_{\mathrm{RF}} \quad \text{(S76)}$$

$$V_{\mathrm{DQ}}(\tau,0) = \frac{1}{2}\sin^2\frac{A_1\tau}{2}\sin^2\frac{A_2\tau}{2}\,. \quad \text{(S77)}$$

These are the intensities at $t = 0$, which corresponds to the integral of the PDHS spectrum. Experimental values are additionally scaled by the τ-dependence of the electron stimulated echo, which is why we focus on their ratios. We measured SQ- and DQ-PDHS of $\mathbf{RF_2}$ with τ 5 µs and 6 µs and otherwise identical experimental conditions (same number of scans, observer position, RF frequency and $\tau_{\mathrm{RF}} = 220$ µs). We chose the g_y observer position because $\omega_{1,2}$ can be well approximated by $2\pi\nu_\perp$ (see Figure 4C) in the main text. Next, we estimated theoretical values for $V_{\mathrm{SQ}}(\tau,0)$ and $V_{\mathrm{DQ}}(\tau,0)$ using the expression above and the following values: $A_1 = A_{1,\perp} = 2\pi C(^{19}\mathrm{F})/(1.36\,\mathrm{nm})^3 \approx 2\pi\cdot 29.6\,\mathrm{kHz}$, $A_2 = A_{2,\perp} = 2\pi C(^{19}\mathrm{F})/(1.42\,\mathrm{nm})^3 \approx 2\pi\cdot 26.0\,\mathrm{kHz}$, $\omega_{1,2} = 2\pi\nu_\perp = 2\pi\cdot 1.0\,\mathrm{kHz}$ and $\tau_{\mathrm{RF}} = 220$ µs, where the distances were taken from the DFT-optimized structures. The experimental and theoretical values are compared in Figure S22. We note good agreement between our simple estimate and the experimental values for both τ's. The increasing DQ-contribution with increased τ is also reproduced.

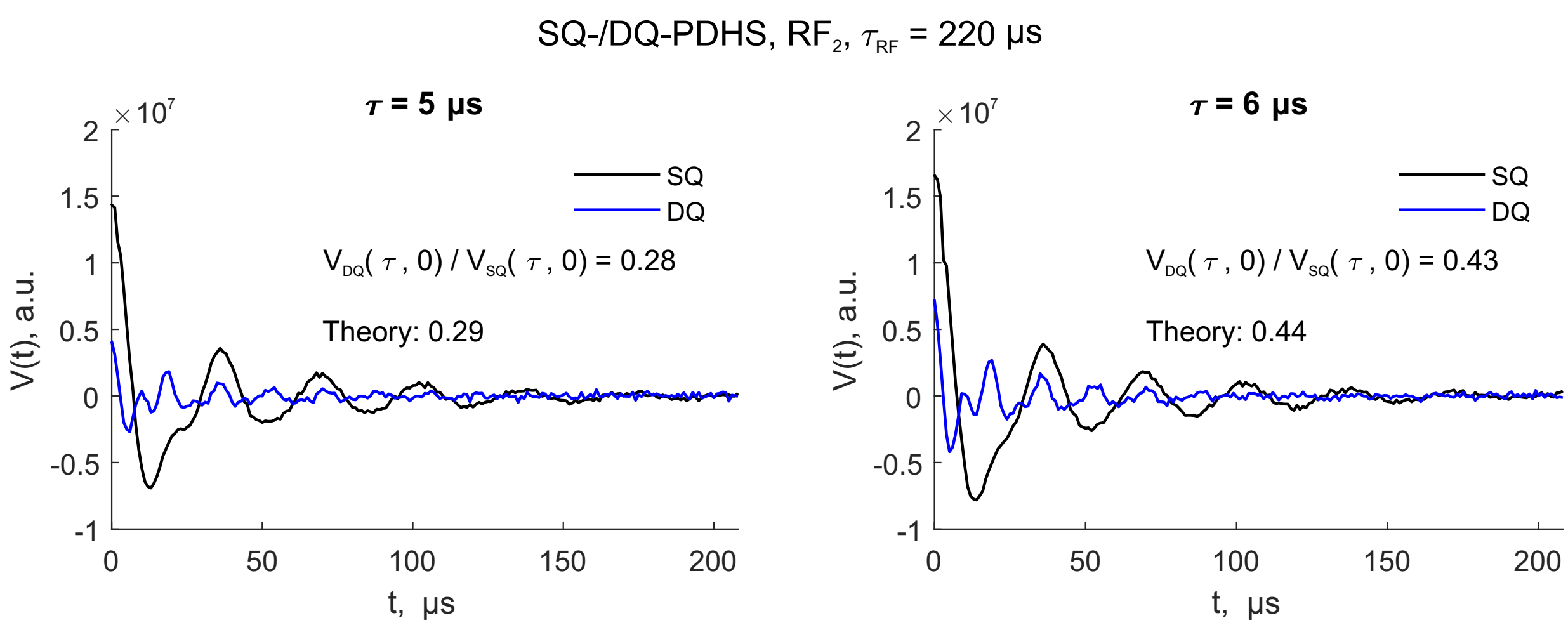

Figure S22: **Experimental evaluation of ratio of SQ- and DQ-contributions in PDHS with compound $\mathbf{RF_2}$.** The traces were recorded at 50 K with $\tau_{\mathrm{RF}} = 220$ µs at the g_y observer position, using the same number of scans. The uncertainties of experimental values are ± 0.01. The theoretical estimation is introduced in the text.

S11 PDHS measurements with variable τ_{RF} of compound RF_2

NEEM data on model compound $\mathbf{RF_2}$ demonstrated that specific values of τ_{RF} can lead to a strong depletion of the nuclear spin echo due to nuclear interaction rather than nuclear relaxation. We measured an SQ-filtered and DQ-filtered PDHS with different τ_{RF} values (Fig. S23).

As expected, we observe no signal in SQ-PDHS. Additionally, we demonstrate that DQ-filtered PDHS still shows a signal at this specific τ_{RF} value, as expected from Eq. (21) in the main text.

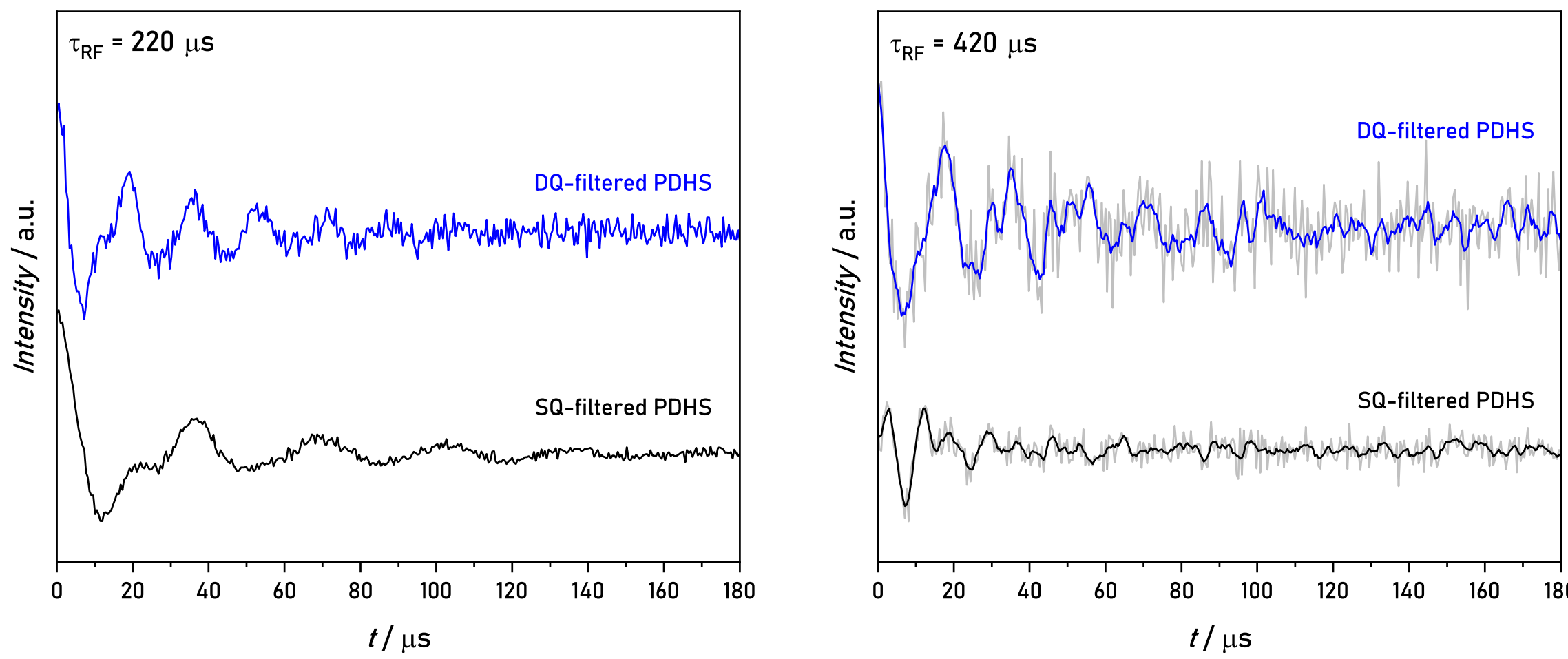

Figure S23: **PDHS experiments of model compound $\mathbf{RF_2}$.** The measurements were performed with a SQ- or DQ-filter phase cycle at 50 K with 12 ns MW $\pi/2$ pulses, 4 µs RF $\pi/2$ pulses, $\tau = 3$ µs (SQ), $\tau = 9$ µs (DQ), 6 ms srt, 10 spp and the MW π pulse was stepped in 960 ns steps for SQ-PDHS with $\tau_{RF} = 420$ µs. In all other cases, 480 ns steps were used. Left panel: Experiments performed with $\tau_{RF} = 220$ µs. Right panel: Experiments performed with $\tau_{RF} = 420$ µs. Grey traces indicate raw data. Colored traces were smoothed with a Savitzky-Golay filter (window size: 10 points)

S12 Fit parameters of SQ- and DQ-PDHS of compound RF_2

phase cycle	$\langle r \rangle$/Å	σ/Å	FWHM / Å	r_{DFT}/Å
SQ	13.54 ± 0.01	0.34 ± 0.01	0.80 ± 0.02	13.6, 14.2
DQ	13.72 ± 0.01	0.17 ± 0.01	0.40 ± 0.02	13.6, 14.2

Table S5: **PDHS distance distributions of $\mathbf{RF_2}$.** Fitting parameters of SQ- and DQ-filtered PDHS acquired for model compound RF_2. $\langle r \rangle$ and σ are the mean and the standard deviation of the Gaussian model for $P(r)$. FWHM is full width at half maximum. r_{DFT} are the distances extracted from the DFT-optimized structure.

S13 FD ENDOR spectrum of RFF'

PDHS spectrum of **RFF'** presented in the main text clearly resolves two distances. Two underline the resolution advantage of PDHS, FD Mims ENDOR experiment with **RFF'** was additionally performed. Figure S24 compares the FD Mims ENDOR and PDHS spectra of **RFF'**.

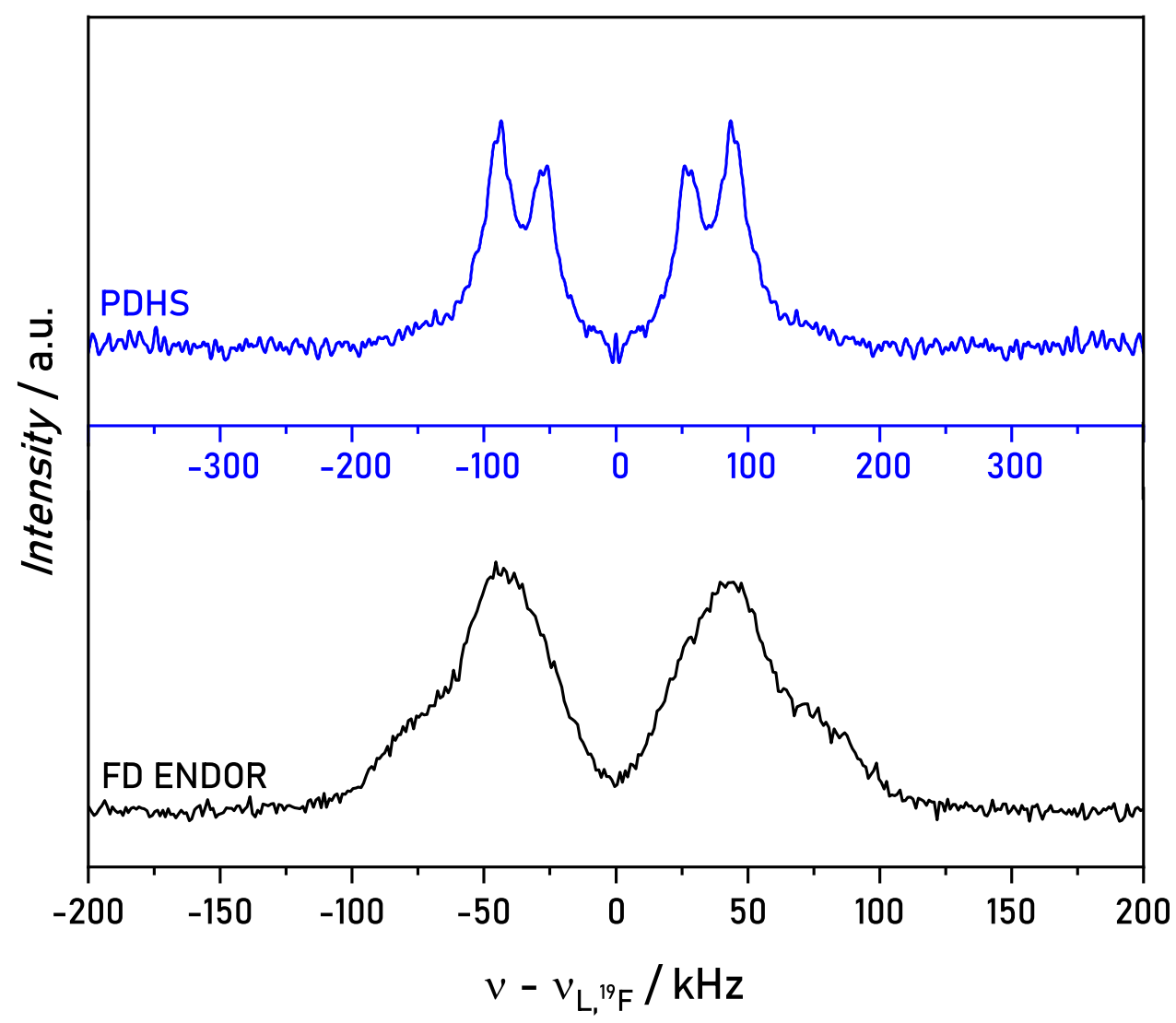

Figure S24: **Comparison of PDHS and FD Mims ENDOR with RFF'.** PDHS and FD ENDOR were recorded at 50 K with 12 ns MW $\pi/2$ pulses, 4 µs RF $\pi/2$ pulses (PDHS), 50 µs RF π pulse (FD Mims ENDOR), $\tau = 2$ µs (PDHS), $\tau = 3$ µs (FD Mims ENDOR), 6 ms srt, 10 spp at the g_y observer position.

S14 DQ-PDHS of RFF’

DQ-PDHS allows distinguishing between two distances arising from different molecular conformations of a single fluorine nucleus or from two different fluorine nuclei in the same molecule. To highlight this advantage, we conducted a DQ-PDHS experiment with **RFF’** (Figure S25).

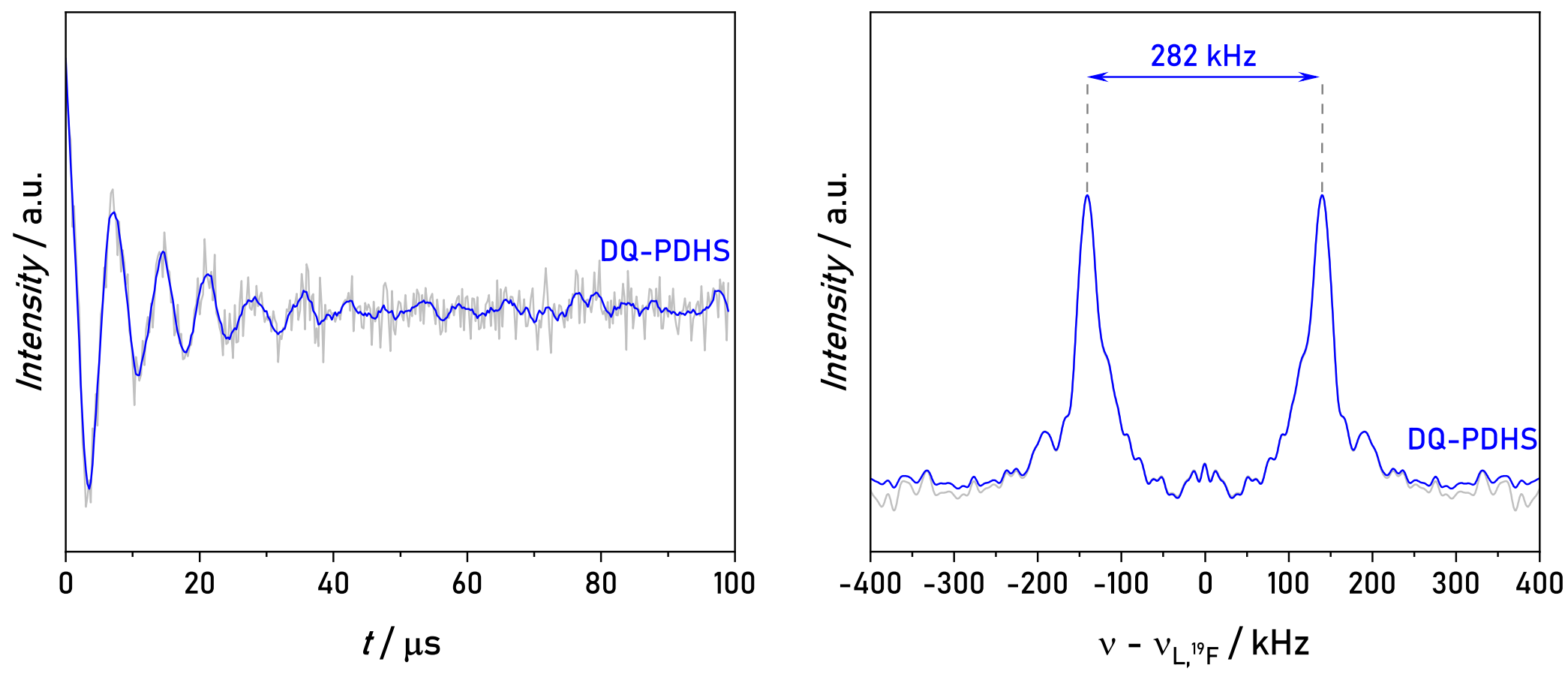

Figure S25: **DQ-PDHS experiment of RFF’.** DQ-PDHS was recorded at 50 K with 12 ns MW π/2 pulses, 4 µs RF π/2 pulses, τ = 5 µs, 6 ms srt, 10 spp and 250 ns steps at the g_y observer position. Left: DQ-PDHS time trace. Grey traces indicate raw data. Colored traces were smoothed with a Savitzky-Golay filter (window size: 15 points). Right: Fourier transform of the DQ-PDHS time trace.

We observe a single splitting of $A_1 + A_2 = 282$ kHz in agreement with a sum of the small ($A_1 = 110$ kHz) and large hyperfine coupling ($A_2 = 176$ kHz) observed in SQ-PDHS (see Figure 7 in the main text).

S15 Simulation of NEEM with a CF_3 group

S15.1 Simulation of NEEM in HFA

We derived the analytical expression for NEEM in nuclear high-field approximation in Eq. (S58). This expression applies if the condition of the weak homonuclear coupling is satisfied, which is $|\omega_{j,k}| < |A_j - A_k|$. The simulations with this formula for compound $\mathbf{RCF_3}$ are shown in Figure S26 (left). Only the SQ-contribution from Eq. (S58) was considered in the simulation. We found the agreement with the experiment not satisfactory, which motivated us to try the strategy described in the next Section.

S15.2 Simulation using an effective nuclear dipolar coupling

If the weak homonuclear coupling condition is not fulfilled, as in a distant CF_3 group, the flip-flop (pseudo-secular term) must be added to the nuclear dipolar coupling Hamiltonian:

$$\hat{H}_{\text{hom.nd.}} = \sum_{j<k} \omega_{j,k} \left(\hat{I}_{j,z}\hat{I}_{k,z} - \frac{\hat{I}_j^+\hat{I}_k^- + \hat{I}_j^-\hat{I}_k^+}{4} \right) . \tag{S78}$$

For two nuclei, the solution with this Hamiltonian is known (*73*). In the weak coupling limit ($|\omega_{1,2}| \ll |A_1 - A_2|$), this Hamiltonian leads to NMR signal splitting with $\omega_{1,2}$, and in the strong coupling limit ($|\omega_{1,2}| \gg |A_1 - A_2|$), with $\frac{3}{2}\omega_{1,2}$. For more than two hyperfine-coupled nuclei with different couplings, there is no analytical solution. We attempted to treat this case approximately by assuming that the flip-flop term leads to a weak level shift. The shift of the hyperfine levels was neglected, and only the change in nuclear dipolar energy was considered. We used the following *ad hoc* expression for the effective nuclear couplings

$$\tilde{\omega}_{j,k} = \omega_{j,k} \left(1 + \frac{1}{2} \frac{\omega_{j,k}^2}{(A_j - A_k)^2 + \omega_{j,k}^2} \right) \tag{S79}$$

which satisfies both the weak- and strong-coupling limits. The introduced correction indeed improves the agreement between simulation and experiment (see more details in Figure S26). With this simplified approach, we were able to reproduce the total width and several shape features; however, quantitative agreement was not achieved.

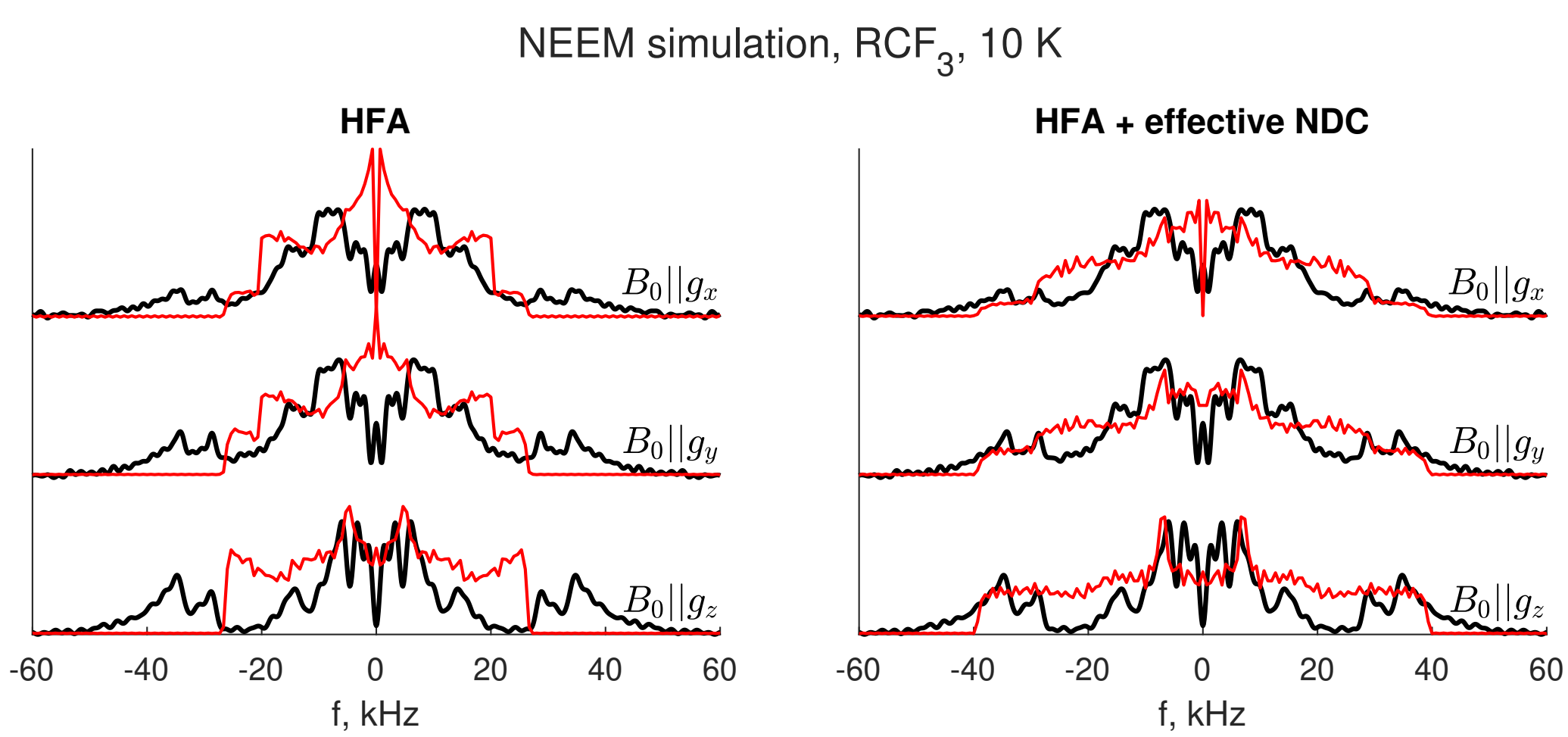

Figure S26: **Experimental and simulated SQ-NEEM spectra for $\mathbf{RCF_3}$.** Experimental spectra (black) were obtained at 10 K. Left: simulation (red) in high-field approximation with Eq. (S58) with no correction for strong nuclear coupling; right: simulation (red) in high-field approximation with an *ad hoc* correction in Eq. (S79). Experimental parameters are given in the main text.

Analysis of simulated data also outlined why the model (S58) is not complete for CF_3. Indeed, as pointed out in Section *'Mixing block and principles of multiple quantum ENDOR spectroscopy'* in the main text, after

the RF excitation of a three-nuclear spin order $\hat{S}_z\hat{I}_{1,z}\hat{I}_{2,z}\hat{I}_{3,z}$, coherences of order three and one are generated. The latter are mixed by the homonuclear coupling as in Eq. (S78), hence, the last term in Eq. (S58) becomes τ_{RF}-dependent. Spin dynamics simulations with four spins are still computationally cheap; however, we express hope that an approximate analytical model and a better approximation than in Eq. (S79) can be found.

S15.3 Simulation with density matrix propagation

Since the analytical solution for NEEM with the Hamiltonian (S78) is unavailable and the heuristic correction did not work immediately, we proceeded with simulations based on the numerical propagation of the density matrix during the mixing block.

Briefly, the numerical simulation was performed by setting the spin Hamiltonian (in angular frequency units)

$$\hat{H}_{\mathrm{free}} = \hat{H}_{\mathrm{hf}} + \hat{H}_{\mathrm{hom.\ nd.}} = \sum_{j=1}^{3} A_j \hat{S}_z \hat{I}_{j,z} + \sum_{j=1}^{3}\sum_{k=j+1}^{3} \omega_{j,k}\left(\hat{I}_{j,z}\hat{I}_{k,z} - \frac{\hat{I}_j^+\hat{I}_k^- + \hat{I}_j^-\hat{I}_k^+}{4}\right) \tag{S80}$$

where the coupling constants were computed in the point-dipole approximation, and thus, they depend on the orientation of the magnetic field in the molecular frame. We used a density matrix after the preparation block (see Eq. (S27)) as the initial state

$$\sigma_{\mathrm{st}} = \sum_{X\subseteq\{1,2,3\}} 2^{|X|} c_g(X,\tau)\hat{S}_z\hat{I}_{X,z} \tag{S81}$$

whereby only $|X| = 1$ and 3 were used for SQ-NEEM and $|X| = 2$ were used for DQ-NEEM. We validated separately that the pseudo-secular homonuclear interaction is negligible during the preparation and detection blocks, so the analytical expressions for $c_g(X,\tau)$ derived in this work can be used up to $\tau = 9\,\mu\mathrm{s}$ in these simulations. The evolution of σ_{st} in the mixing block was calculated by iteratively computing the time propagation as

$$\sigma(t+\Delta t) = U(\Delta t)\sigma(t)U(\Delta t)^\dagger \tag{S82}$$

where $U(\Delta t) = \exp(-i\hat{H}_{\mathrm{free}}\Delta t)$ for free evolution and $U(\Delta t) = \exp(-i\hat{H}_{\mathrm{RF,p}}\Delta t)$ for an RF pulse. In the ideal pulse approximation,

$$\hat{H}_{\mathrm{RF,p}} = \nu_2\hat{F}_\phi\ , \tag{S83}$$

where $\nu_2 = 62.5\,\mathrm{kHz}$ is the Rabi frequency corresponding to $8\,\mu\mathrm{s}$ π pulse and $\hat{F}_\phi = \hat{I}_{1,\phi} + \hat{I}_{2,\phi} + \hat{I}_{3,\phi}$. We used $\Delta t = 4\,\mu\mathrm{s}$ for RF $\pi/2$ pulses and $\Delta t = 8\,\mu\mathrm{s}$ for the RF π pulses, in agreement with experimental values. Importantly, we found that for $\mathbf{RCF_3}$ with the relatively short nitroxide-^{19}F distance, it was necessary to treat the finite bandwidth of the RF pulses, i.e. the free Hamiltonian was added to the pulse Hamiltonian,

$$\hat{H}_{\mathrm{RF,p}} = \nu_2\hat{F}_\phi + \hat{H}_{\mathrm{free}}\ . \tag{S84}$$

The EPR signal was computed as $V_{\phi_1,\psi,\phi_2}(\tau_{\mathrm{RF}}) = \mathrm{tr}(\sigma(\phi_1,\psi,\phi_2,\tau_{\mathrm{RF}})\sigma'_{\mathrm{det}})$ (see Section S1.8), where ϕ_1, ψ and ϕ_2 are the phases of the RF pulses. The NEEM traces were computed as functions of the delay τ_{RF} and magnetic field orientation $\mathbf{n}$. The traces for different orientations were summed with weights computed in Eq. (26) in the main text, which accounts for the orientation selection. The computation was repeated for $\phi_1 = [0, \pi/2, \pi, 3\pi/2]$ and summed with weights $[+1,+1,-1,-1]$ for SQ-NEEM and $[+1,-1,+1,-1]$ for DQ-NEEM. The redundant phase cycling for the SQ-NEEM allowed for better elimination of unwanted coherence pathways. In our simulations, we used $\psi = \phi_2 = 0$.

The initial simulation of the SQ-NEEM traces/spectra (g_z observer position, 10 K) is shown in Figure S27. The resolution of our experimental data is sufficient to infer that the $^{19}\mathrm{F}-^{19}\mathrm{F}$ coupling calculated from the DFT-optimised structure underestimates the true value by 3-4%. This can arise from a slightly offset distance (the real distance can be $\approx 1\%$ shorter than that predicted by DFT) or from vibrational averaging of the coupling constant that depends non-linearly on the distance (r^{-3}).

The simulations of the orientation-selective SQ- and DQ-NEEM data (10 K) with the adjusted nuclear coupling are shown in Figures S28 and S29.

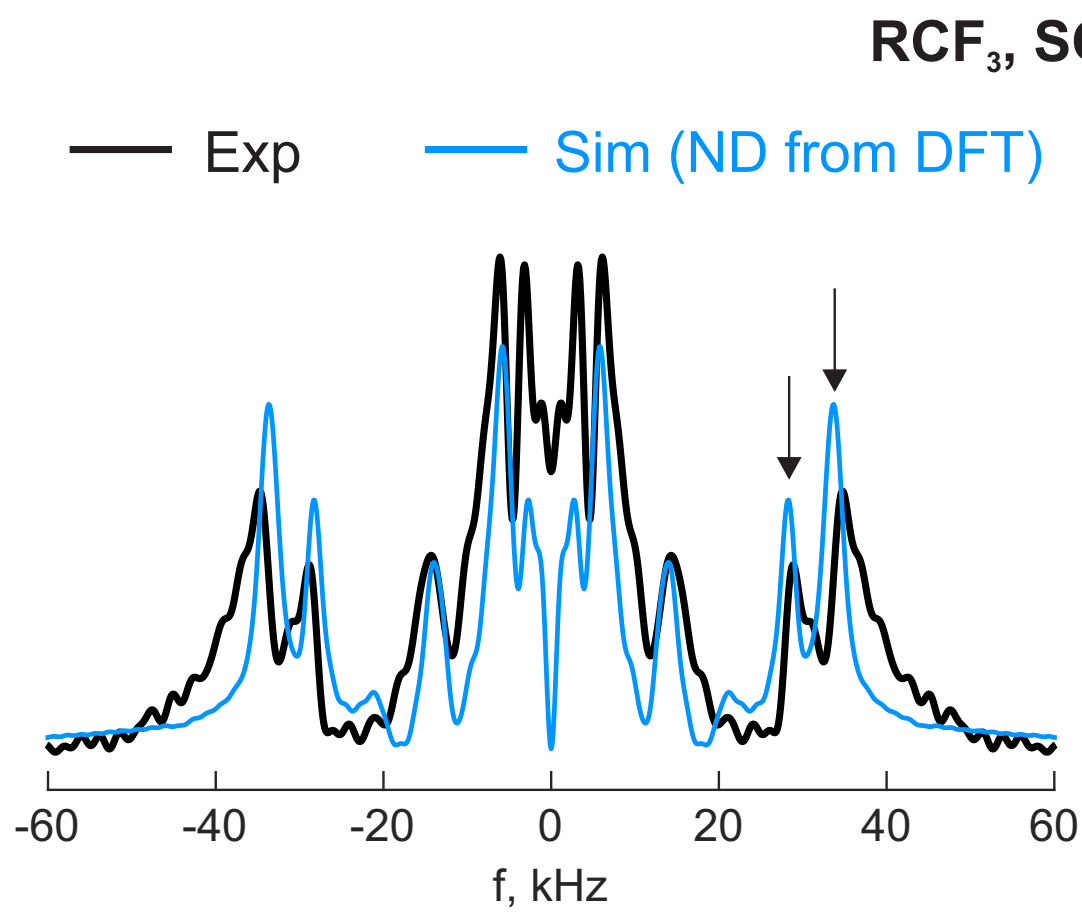

Figure S27: **Simulation of SQ-NEEM for compound RCF$_3$.** Here, the extended homonuclear coupling Hamiltonian (Eq. (S78)) was used. Black line is experimental data (10 K, detection at g_z), blue line – simulation with F-F distance taken from DFT-optimised structure (2.187 Å). Red line – simulation with adjusted F-F distance (2.152 Å), which fits the position of two outer peaks.

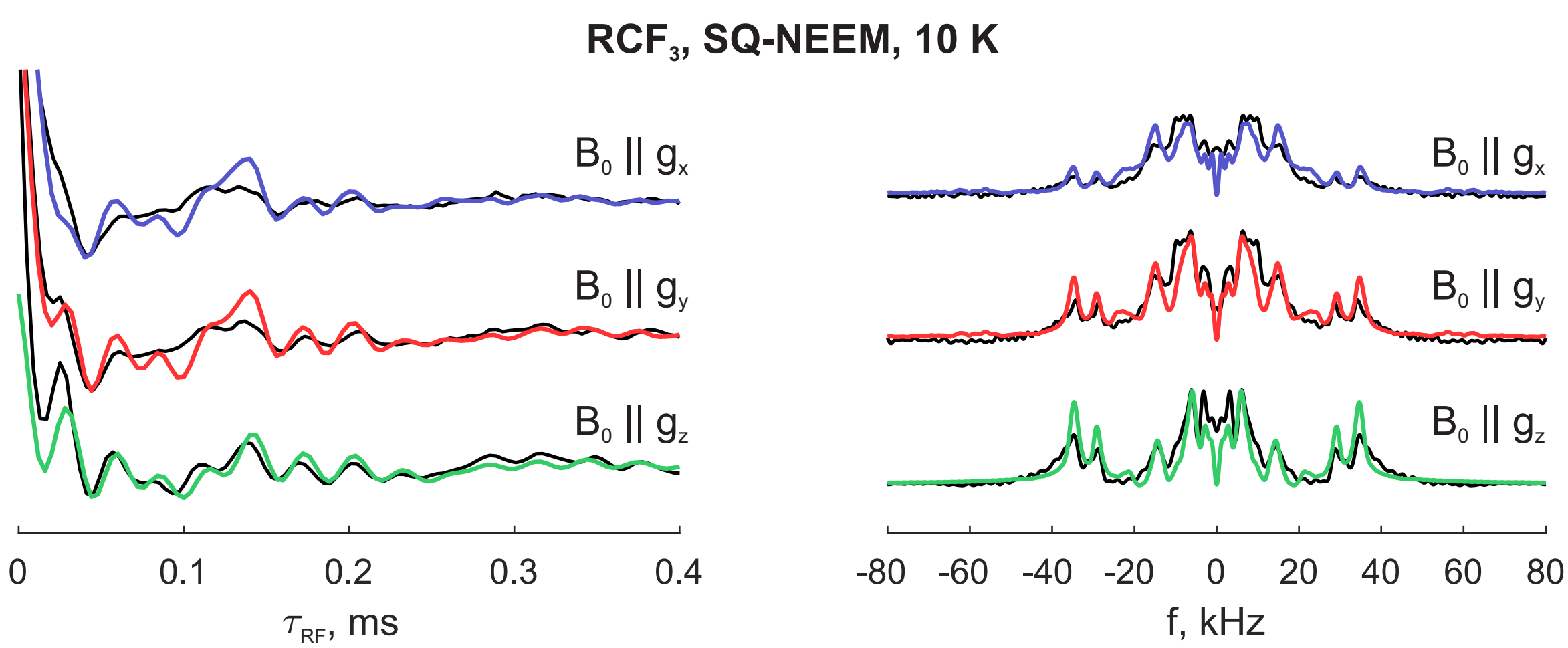

Figure S28: **Numerical simulation of SQ-NEEM for compound RCF$_3$.** The data ($\tau = 3\,\mu s$) are shown at three observer positions in the EPR spectrum (colored lines) and compared with experiment (black lines, background-corrected). Experimental data were acquired at 10 K.

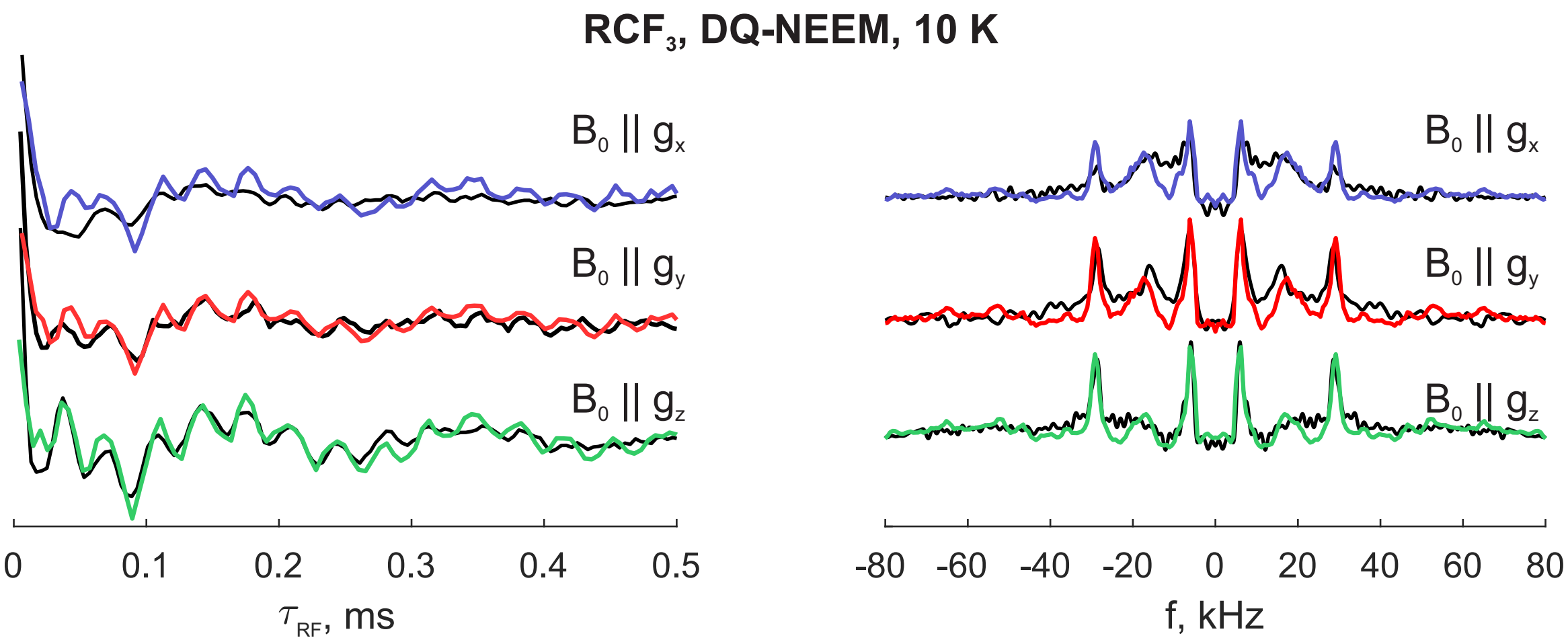

Figure S29: **Numerical simulation of DQ-NEEM for compound RCF$_3$.** The data ($\tau = 5\,\mu s$) are shown at three observer positions in the EPR spectrum (colored lines) and compared with experiment (black lines, background-corrected). Experimental data were acquired at 10 K.

S16 SQ-NEEM spectrum of CF_3 group at 50 K

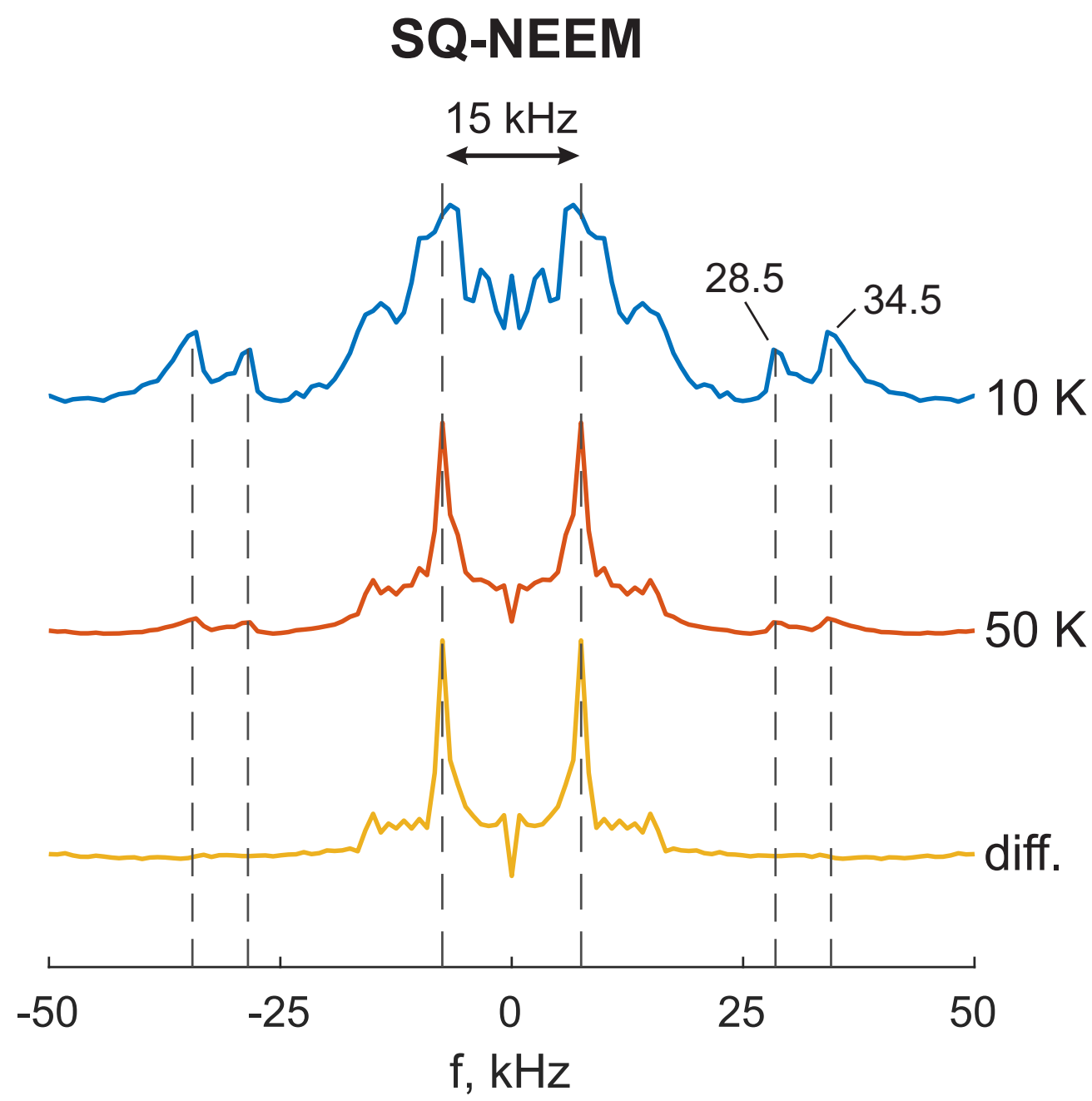

Figure S30: **Temperature dependence of SQ-NEEM with $\mathbf{RCF_3}$.** SQ-NEEM spectrum of $\mathbf{RCF_3}$ ($\tau = 3\,\mu s$, detection at g_y) measured at 10 K (top blue line), 50 K (middle orange line), and their difference (bottom yellow line). At temperatures above 10 K, a sharp feature appears, whose weight gradually increases, and is assigned to mobile CF_3 groups. At 50 K, the spectrum of the frozen CF_3-group is still present, as seen by peaks at 28.5 kHz and 34.5 kHz. The emerging spectrum resembles a Pake pattern with a splitting of 15 kHz.

S17 PDHS of RCF_3 with short τ_{RF} at 10 K and 50 K

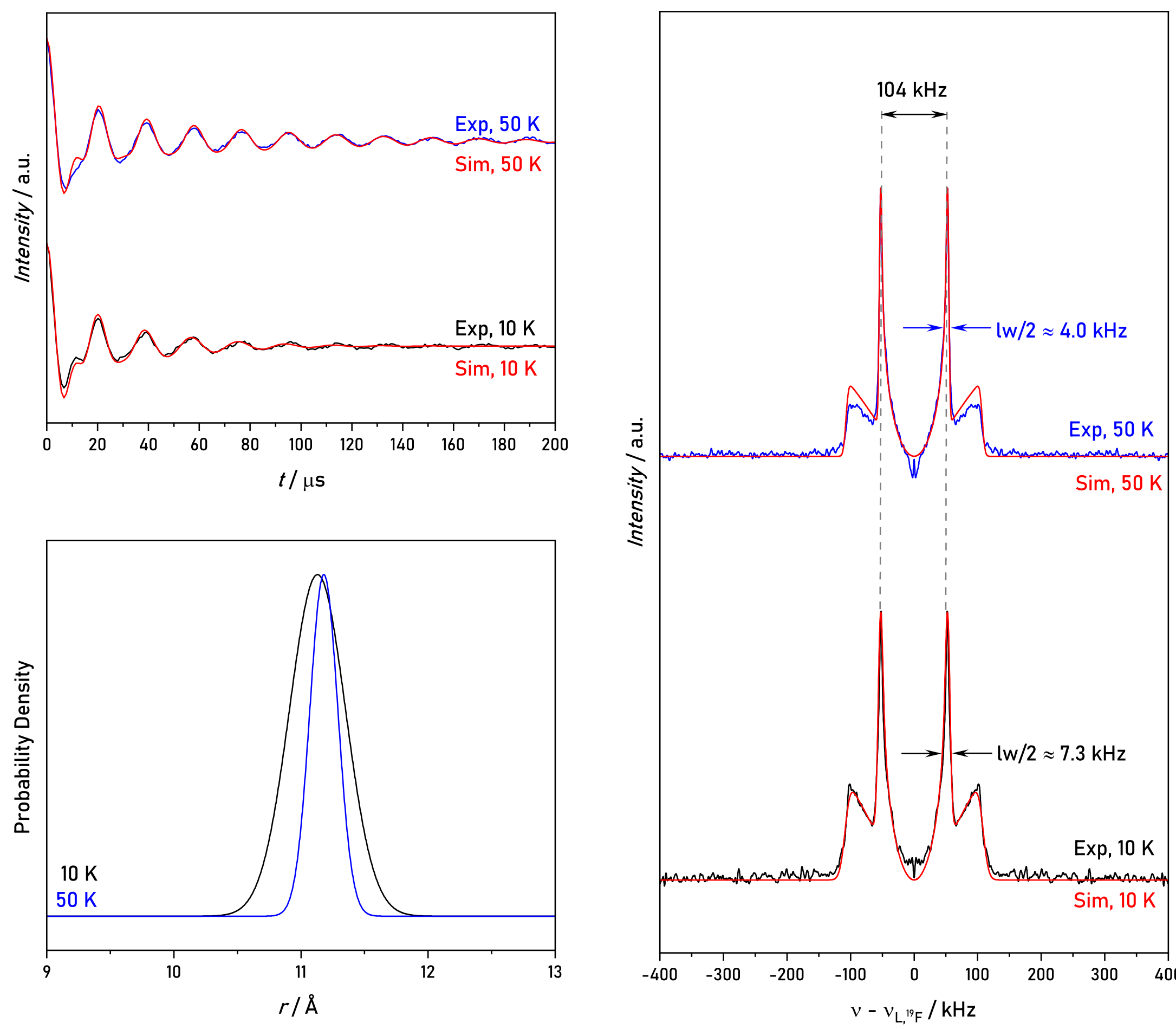

Figure S31: **Temperature dependence of PDHS with $\mathbf{RCF_3}$.** Left: SQ/TQ-filtered PDHS traces of model compound $\mathbf{RCF_3}$ measured at 10 K and 50 K ($\tau = 3\,\mu s$). Simulations are shown as solid red lines, and the resulting distance distributions are shown in the panel below. Simulation parameters are summarized in Table S6. Right: Fourier transform of the time-domain data. Experimental parameters: 12 ns MW $\pi/2$ pulses, 4 μs RF $\pi/2$ pulses, $\tau_{RF} = 220\,\mu s$, srt 500 ms (10 K) and 5 ms (50 K) and the MW π pulse was stepped in 480 ns steps.

(SQ-)PDHS spectra (Figure S31) exhibit a main splitting of ≈ 104 kHz which is expected for fluorines at a distance $r \approx 11.3$ Å. Temperature has a pronounced effect on the PDHS line width: increasing the temperature to 50 K decreases the line width by $\approx 45\%$, as evidenced by weaker damping in the corresponding time traces. We attribute this narrowing to motional averaging of the hyperfine couplings caused by rotation of the CF_3 group. Following the additive approximation, the PDHS traces were simulated using the dipolar kernel previously employed for mono-fluorinated compounds (see Table S6). DFT-predicted distances of $\mathbf{RCF_3}$ shown in Table S6 are based on the A tensors of each ^{19}F in a single rotamer/structure of the CF_3 group. However, rotation around the C-C bond of the CF_3 group merely changes the fluorine distances. We rotated the CF_3 group in steps of 10° and found fluorine distances in a range of $11.1 - 11.5$ Å. We additionally tested that the multispin contributions have a weak effect on simulations for $\tau = 3\,\mu s$ (Figure S33). Under these conditions, experiment and simulation show good agreement. In this model, the line broadening at 10 K encodes a correspondingly broader distance distribution in the fit (see Table S6). Consequently, the extracted distance distributions are influenced by the

internal motion of the CF_3 group. This demonstrates that PDHS is also a suitable tool to investigate the rotational dynamics of CF_3.

Table S6: **Simulation parameters of SQ- and DQ-filtered PDHS acquired for compound $\mathbf{RCF_3}$.** $\langle r \rangle$ and σ denote the mean and standard deviation of the Gaussian model for $P(r)$. Kernel refers to the specific analytical form of the dipolar kernel (see Eqs. (30)–(37) in the main text).

phase cycle	τ / µs	kernel	$\langle r \rangle$ / Å	σ / Å	FWHM / Å	r_{DFT} / Å
SQ (10 K)	3	$K_{\mathrm{SQ}}^{(1)}(r,t,\tau)$	11.13 ± 0.01	0.22 ± 0.02	0.52 ± 0.04	11.1–11.5
SQ (50 K)	3	$K_{\mathrm{SQ}}^{(1)}(r,t,\tau)$	11.18 ± 0.01	0.11 ± 0.01	0.26 ± 0.02	
SQ (10 K)	9	$K_{\mathrm{SQ}}^{(3)}(r,t,\tau,f)$	11.00 ± 0.01	0.20 ± 0.02	0.47 ± 0.04	
DQ (10 K)	9	$K_{\mathrm{DQ}}^{(3)}(r,t,\tau)$	11.00 ± 0.01	0.14 ± 0.01	0.40 ± 0.02	

S18 PDHS simulations of RCF_3 with different kernel functions

To simulate the DQ-PDHS data for $\mathbf{RCF_3}$, we had to extend our dipolar kernel by including the geometry of the CF_3 group. The explicit consideration of molecular geometry is essential in this case. If the three fluorine nuclei are treated as placed colinearly or isotropically (fully uncorrelated) without accounting for the trigonal arrangement, the simulation predicts incorrect modulation frequencies and blind spot patterns. Figure S32 compares the simulation of DQ-filtered PDHS using a kernel with the approximation of CF_3 as a point object:

$$K^{(3)}_{\text{DQ, point object}}(r,t,\tau) = \int_0^{\pi/2} \sin^4 \frac{A\tau}{2} \cos^2 \frac{A\tau}{2} \cdot \sin\theta \cdot \cos 2At \, d\theta\,. \tag{S85}$$

and the kernel used in the main text (Eq. (35)).

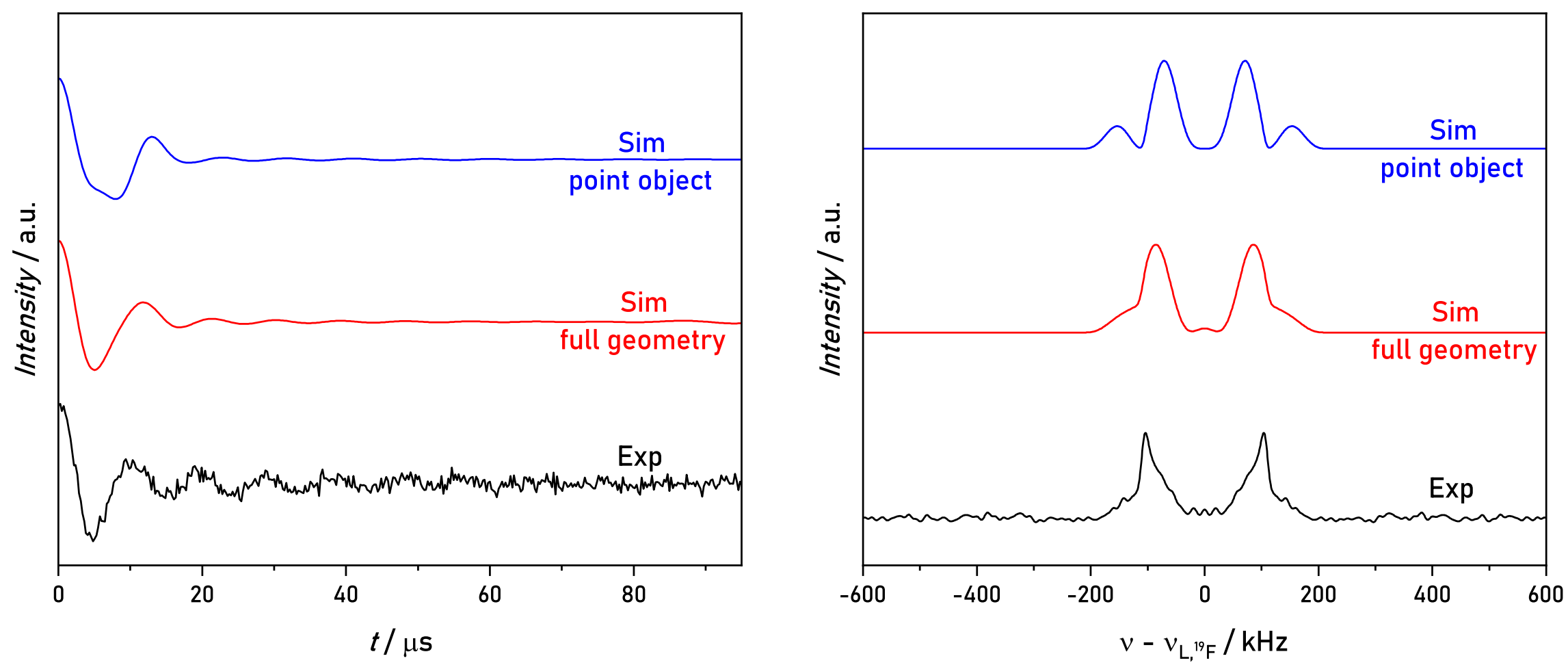

Figure S32: **Different simulation approaches for DQ-PDHS with $\mathbf{RCF_3}$.** PDHS experiment of $\mathbf{RCF_3}$ (10 K, $\tau = 9\,\mu s$) performed with a DQ-filter phase cycle. Additional experimental parameters are provided in Figure 9 in the main text. The computed traces were based on the same Gaussian distance distribution with a mean of 11 Å and a standard deviation of 0.14 Å (see Table S6). The simulation using a kernel that accounts for the geometry of the CF_3 group is shown in red. The simulation using an approximation of the CF_3 group as a point object is shown in blue.

The approximation of CF_3 as a point object leads to blind spots at $\approx$ 115 kHz, which is not observed in the experimental spectrum. The explicit consideration of the geometry of the CF_3 group complicates the extraction of distance distributions at high τ values. Using shorter τ values suppresses multispin effects in PDHS, thereby allowing us to approximate the CF_3 group as a point object again. Figure S33 compares a PDHS experiment and simulations with short τ values using a kernel that accounts for the CF_3 geometry (Eq. (35) in the main text) and a simple single-spin kernel (see Eq. (30)).

For such a short τ value, both kernels show good agreement between simulation and experiment.

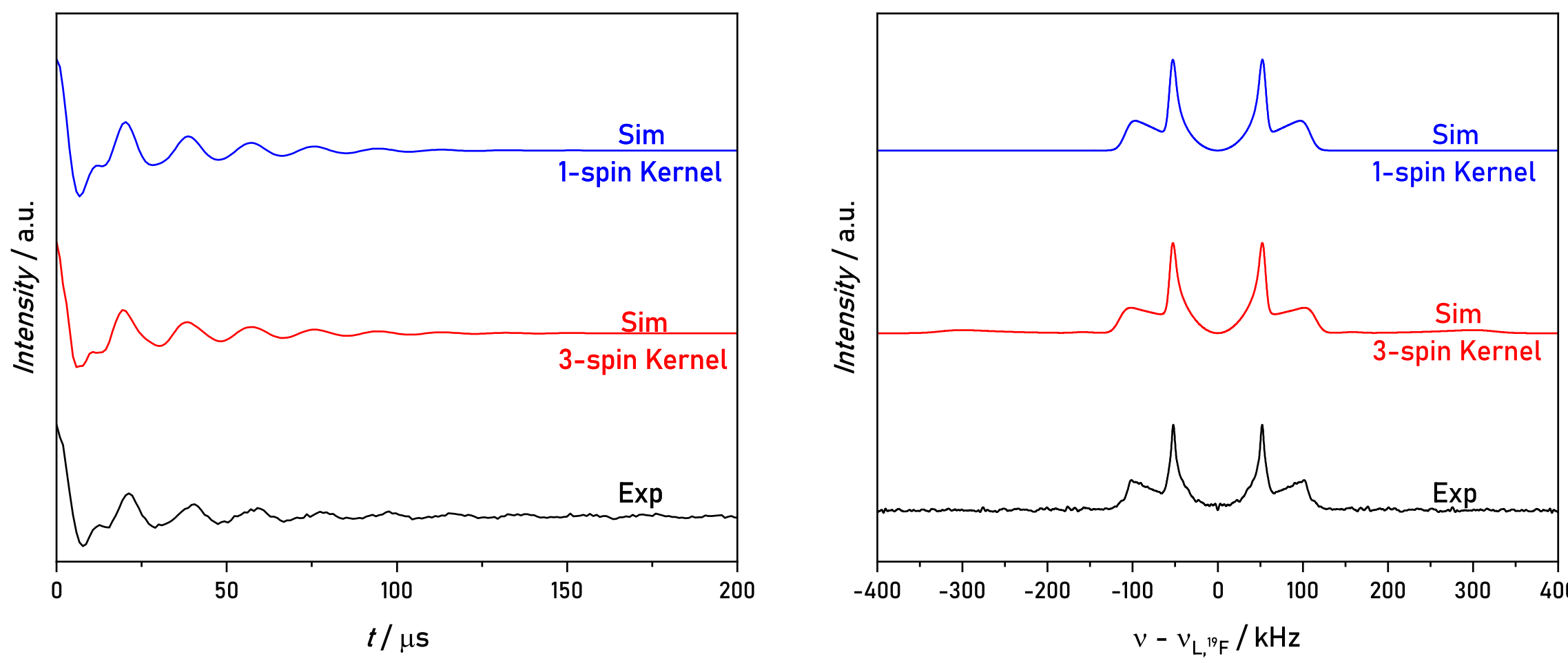

Figure S33: **Different simulation approaches for SQ/TQ-PDHS with $\mathbf{RCF_3}$.** PDHS experiments of $\mathbf{RCF_3}$ acquired at 10 K with $\tau = 3\,\mu s$ with a SQ-filter phase cycle (black line) and their simulations. Additional experimental parameters are provided in Figure 9 in the main text. Simulation using a one-spin kernel is shown in blue. Simulation using a three-spin kernel is shown as solid red lines. Left panel: PDHS time traces. Right panel: corresponding PDHS spectra.

REFERENCES

1. W. Mims, Pulsed ENDOR experiments. *Proc. R. Soc. Lond. A Math. Phys. Sci.* **283**, 452–457 (1965).

2. E. Davies, A new pulse ENDOR technique. *Phys. Lett. A* **47**, 1–2 (1974).

3. J. R. Harmer, Hyperfine spectroscopy–ENDOR in*EPR Spectroscopy: Fundamentals and Methods*, D. Goldfarb, S. Stoll, Eds. (Wiley & Sons, 2018).

4. M. O. Ross, F. MacMillan, J. Wang, A. Nisthal, T. J. Lawton, B. D. Olafson, S. L. Mayo, A. C. Rosenzweig, B. M. Hoffman, Particulate methane monooxygenase contains only mononuclear copper centers. *Science* **364**, 566–570 (2019).

5. J. M. Kuchenreuther, W. K. Myers, D. L. Suess, T. A. Stich, V. Pelmenschikov, S. A. Shiigi, S. P. Cramer, J. R. Swartz, R. D. Britt, S. J. George, The HydG enzyme generates an $Fe(CO)_2(CN)$ synthon in assembly of the FeFe hydrogenase H-cluster. *Science* **343**, 424–427 (2014).

6. N. Cox, M. Retegan, F. Neese, D. A. Pantazis, A. Boussac, W. Lubitz, Electronic structure of the oxygen-evolving complex in photosystem II prior to OO bond formation. *Science* **345**, 804–808 (2014).

7. A. A. Malär, N. Wili, L. A. Völker, M. I. Kozlova, R. Cadalbert, A. Däpp, M. E. Weber, J. Zehnder, G. Jeschke, H. Eckert, Spectroscopic glimpses of the transition state of ATP hydrolysis trapped in a bacterial DnaB helicase. *Nat. Commun.* **12**, 5293 (2021).

8. P. J. Carl, D. E. Vaughan, D. Goldfarb, High field 27Al ENDOR reveals the coordination mode of Cu2+ in low Si/Al zeolites. *J. Am. Chem. Soc.* **128**, 7160–7161 (2006).

9. F. Hecker, J. Stubbe, M. Bennati, Detection of water molecules on the radical transfer pathway of ribonucleotide reductase by ^{17}O electron–nuclear double resonance spectroscopy. *J. Am. Chem. Soc.* **143**, 7237–7241 (2021).

10. S. B. Orlinskii, J. Schmidt, P. G. Baranov, M. Bickermann, B. M. Epelbaum, A. Winnacker, Observation of the triplet metastable state of shallow donor pairs in aln crystals with a negative-u behavior: A high-frequency epr and endor study. *Phys. Rev. Lett.* **100**, 256404 (2008).

11. G. Rao, A. B. Altman, A. C. Brown, L. Tao, T. A. Stich, J. Arnold, R. D. Britt, Metal bonding with 3d and 6d orbitals: An EPR and ENDOR spectroscopic investigation of Ti3+–Al and Th3+–Al heterobimetallic complexes. *Inorg. Chem.* **58**, 7978–7988 (2019).

12. E. Schleicher, S. Rein, B. Illarionov, A. Lehmann, T. Al Said, S. Kacprzak, R. Bittl, A. Bacher, M. Fischer, S. Weber, Selective 13C labelling reveals the electronic structure of flavocoenzyme radicals. *Sci. Rep.* **11**, 18234 (2021).

13. P. Faller, T. Maly, A. W. Rutherford, F. MacMillan, Chlorophyll and carotenoid radicals in photosystem II studied by pulsed ENDOR. *Biochemistry* **40**, 320–326 (2001).

14. A. Meyer, S. Dechert, S. Dey, C. Höbartner, M. Bennati, Measurement of angstrom to nanometer molecular distances with ^{19}F nuclear spins by EPR/ENDOR spectroscopy. *Angew. Chem. Int. Ed. Engl.* **59**, 373–379 (2020).

15. N. Asanbaeva, A. Sukhanov, A. Diveikina, O. Rogozhnikova, D. Trukhin, V. Tormyshev, A. Chubarov, A. Maryasov, A. Genaev, A. Shernyukov, G. Salnikov, A. Lomzov, D. Pyshnyi, E. Bagryanskaya, Application of W-band ^{19}F electron nuclear double resonance (ENDOR) spectroscopy to distance measurement using a trityl spin probe and a fluorine label. *Phys. Chem. Chem. Phys.* **24**, 5982–6001 (2022).

16. M. Judd, E. H. Abdelkader, M. Qi, J. R. Harmer, T. Huber, A. Godt, A. Savitsky, G. Otting, N. Cox, Short-range ENDOR distance measurements between Gd (iii) and trifluoromethyl labels in proteins. *Phys. Chem. Chem. Phys.* **24**, 25214–25226 (2022).

17. N. Asanbaeva, D. S. Novopashina, O. Y. Rogozhnikova, V. M. Tormyshev, A. Kehl, A. Sukhanov, A. V. Shernyukov, A. Genaev, A. A. Lomzov, M. Bennati, A. Meyer, E. G. Bagryanskaya, ^{19}F electron nuclear double resonance (ENDOR) spectroscopy for distance

measurements using trityl spin labels in DNA duplexes. *Phys. Chem. Chem. Phys.* **25**, 23454–23466 (2023).

18. S. L. Schumann, S. Kotnig, Y. Kutin, M. Drosou, L. M. Stratmann, Y. Streltsova, A. Schnegg, D. A. Pantazis, G. H. Clever, M. Kasanmascheff, Structure and flexibility of copper-modified DNA G-quadruplexes investigated by ^{19}F ENDOR experiments at 34 GHz. *Chemistry* **29**, e202302527 (2023).

19. A. Bogdanov, L. Gao, A. Dalaloyan, W. Zhu, M. Seal, X.-C. Su, V. Frydman, Y. Liu, A. M. Gronenborn, D. Goldfarb, Spin labels for ^{19}F ENDOR distance determination: Resolution, sensitivity and distance predictability. *Phys. Chem. Chem. Phys.* **26**, 26921–26932 (2024).

20. L. Remmel, A. Meyer, K. Ackermann, G. Hagelueken, M. Bennati, B. E. Bode, Pulsed EPR methods in the angstrom to nanometre scale shed light on the conformational flexibility of a fluoride riboswitch. *Angew. Chem. Int. Ed. Engl.* **63**, e202411241 (2024).

21. M. Gauger, M. Heinz, A.-L. J. Halbritter, L. S. Stelzl, N. Erlenbach, G. Hummer, S. T. Sigurdsson, T. F. Prisner, Structure and internal dynamics of short RNA duplexes determined by a combination of pulsed EPR methods and MD simulations. *Angew. Chem. Int. Ed. Engl.* **136**, e202402498 (2024).

22. M. Judd, M. Qi, E. H. Abdelkader, H. Qianzhu, A. Savitsky, T. Huber, J. R. Harmer, A. Godt, G. Otting, N. Cox, Measuring nanometer distances in proteins and rigid rulers between 19F and Gd3+ by integration of 19F-ENDOR signal intensities. *J. Am. Chem. Soc.* **147**, 16826–16835 (2025).

23. M. Judd, E. H. Abdelkader, H. Qianzhu, E. Habel, C. Lindsay, A. Brancewicz, T. Huber, G. Otting, N. Cox, Characterising side chain conformations in proteins using 19F-ENDOR and new fluorinated amino acid spin labels. *Chemistry* **32**, e02647 (2025).

24. A. Meyer, A. Kehl, C. Cui, F. A. Reichardt, F. Hecker, L.-M. Funk, M. K. Ghosh, K.-T. Pan, H. Urlaub, K. Tittmann, J. Stubbe, M. Bennati, ^{19}F electron-nuclear double resonance reveals interaction between redox-active tyrosines across the α/β interface of E. coli ribonucleotide reductase. *J. Am. Chem. Soc.* **144**, 11270–11282 (2022).

25. M. Seal, W. Zhu, A. Dalaloyan, A. Feintuch, A. Bogdanov, V. Frydman, X.-C. Su, A. M. Gronenborn, D. Goldfarb, Gd(III)-^{19}F distance measurements for proteins in cells by electron-nuclear double resonance. *Angew. Chem. Int. Ed. Engl.* **62**, e202218780 (2023).

26. P. Höfer, A. Grupp, M. Mehring, High-resolution time-domain electron-nuclear-sublevel spectroscopy by pulsed coherence transfer. *Phys. Rev. A* **33**, 3519–3522 (1986).

27. P.-P. Zänker, G. Jeschke, D. Goldfarb, Distance measurements between paramagnetic centers and a planar object by matrix Mims electron nuclear double resonance. *J. Chem. Phys.* **122**, 024515 (2005).

28. N. Dayan, Y. Artzi, M. Jbara, D. Cristea, A. Blank, Pulsed electron-nuclear double resonance in the Fourier regime. *ChemPhysChem* **24**, e202200624 (2023).

29. A. Kehl, L. Sielaff, L. Remmel, M. L. Rämisch, M. Bennati, A. Meyer, Frequency and time domain ^{19}F ENDOR spectroscopy: Role of nuclear dipolar couplings to determine distance distributions. *Phys. Chem. Chem. Phys.* **27**, 1415–1425 (2025).

30. O. Schiemann, C. A. Heubach, D. Abdullin, K. Ackermann, M. Azarkh, E. G. Bagryanskaya, M. Drescher, B. Endeward, J. H. Freed, L. Galazzo, D. Goldfarb, H. Tobias, L. E. Hofer, L. F. Ibáñez, E. J. Hustedt, S. Kucher, I. Kuprov, J. E. Lovett, A. Meyer, S. Ruthstein, S. Saxena, S. Stoll, C. R. Timmel, M. Di Valentin, H. Mchaourab, T. F. Prisner, B. E. Bode, E. Bordignon, M. Bennati, G. Jeschke, Benchmark test and guidelines for DEER/PELDOR experiments on nitroxide-labeled biomolecules. *J. Am. Chem. Soc.* **143**, 17875–17890 (2021).

31. L. Sielaff, A. Kehl, A. Aden, A. Meyer, M. Bennati, Pulsed dipolar hyperfine spectroscopy for molecular distance measurements in the angstrom to nanometer scale. *Sci. Adv.* **11**, eady5665 (2025).

32. A. Grupp, M. Mehring, Pulsed ENDOR spectroscopy in solids in *Modern Pulsed and CW Electron Spin Resonance* (Wiley: New York, 1990), pp. 195–229.

33. C. Gemperle, A. Schweiger, Pulsed electron-nuclear double resonance methodology. *Chem. Rev.* **91**, 1481–1505 (1991).

34. M. Mehring, P. Höfer, H. Käß, A. Grupp, Multiple-quantum ENDOR-spectroscopy of protons in trans-polyacetylene. *Europhys. Lett.* **6**, 463–468 (1988).

35. H. Thomann, M. Bernardo, Multiple quantum nuclear coherences in pulsed ENDOR spectroscopy. *Isr. J. Chem.* **32**, 323–337 (1992).

36. C. Gemperle, A. Schweiger, R. Ernst, ESR-detected nuclear transient nutations: Detection schemes and applications. *Chem. Phys. lett.* **145**, 1–8 (1988).

37. A. Wokaun, R. R. Ernst, Selective detection of multiple quantum transitions in NMR by two-dimensional spectroscopy. *Chem. Phys. lett.* **52**, 407–412 (1977).

38. M. Munowitz, A. Pines, Multiple-quantum nuclear magnetic resonance spectroscopy. *Science* **233**, 525–531 (1986).

39. M. Munowitz, A. Pines, Principles and applications of multiple-quantum NMR. *Adv. Chem. Phys.* **66**, 1–152 (1987).

40. S. Saxena, J. H. Freed, Double quantum two-dimensional Fourier transform electron spin resonance: Distance measurements. *Chem. Phys. Lett.* **251**, 102–110 (1996).

41. M. Bretschneider, P. E. Spindler, O. Y. Rogozhnikova, D. V. Trukhin, B. Endeward, A. A. Kuzhelev, E. Bagryanskaya, V. M. Tormyshev, T. F. Prisner, Multiquantum counting of trityl radicals. *J. Phys. Chem. Lett.* **11**, 6286–6290 (2020).

42. A. Sinha Roy, J. A. Marohn, J. H. Freed, An analysis of double-quantum coherence ESR in an N-spin system: Analytical expressions and predictions. *J. Chem. Phys.* **160**, (2024).

43. A. Bogdanov, V. Frydman, X.-C. Su, D. Goldfarb, Decoherence of hyperfine coupled 19F and 1H nuclei in gadolinium (III) model complexes. *J. Phys. Chem. B* **129**, 6604–6612 (2025).

44. A. Kehl, M. Hiller, F. Hecker, I. Tkach, S. Dechert, M. Bennati, A. Meyer, Resolution of chemical shift anisotropy in ^{19}F ENDOR spectroscopy at 263 GHz/9.4 T. *J. Magn. Reson.* **333**, 107091 (2021).

45. T. Nakashima, R. McClung, Simulation of two-dimensional NMR spectra using product operators in the spherical basis. *J. Magn. Reson.* **70**, 187–203 (1986).

46. U. Piantini, O. Sorensen, R. R. Ernst, Multiple quantum filters for elucidating NMR coupling networks. *J. Am. Chem. Soc.* **104**, 6800–6801 (1982).

47. P. Höfer, Multiple quantum pulsed ENDOR spectroscopy by time proportional phase increment detection. *Appl. Magn. Reson.* **11**, 375–389 (1996).

48. D. Sakellariou, A. Lesage, L. Emsley, Spectral editing in solid-state NMR using scalar multiple quantum filters. *J. Magn. Reson.* **151**, 40–47 (2001).

49. A. Schweiger, G. Jeschke, *Principles of Pulse Electron Paramagnetic Resonance* (Oxford University Press, 2001).

50. S. Van Doorslaer, D. Goldfarb, S. Stoll, Hyperfine Spectroscopy-ESEEM. *EPR Spectroscopy: Fundamentals and Methods*, (Wiley, 2018), pp. 377–400.

51. S. A. Dikanov, Y. Tsvetkov, *Electron Spin Echo Envelope Modulation (ESEEM) Spectroscopy* (CRC Press, 2024).

52. J. Keeler, *Understanding NMR Spectroscopy* (John Wiley & Sons, 2010).

53. M. Rubio, M. Merchán, E. Ortí, The internal rotational barrier of biphenyl studied with multiconfigurational second-order perturbation theory (CASPT2). *Theoret. Chim. Acta* **91**, 17–29 (1995).

54. A. Göller, U.-W. Grummt, Torsional barriers in biphenyl, 2, 2′-bipyridine and 2-phenylpyridine. *Chem. Phys. Lett.* **321**, 399–405 (2000).

55. G. E. Pake, Nuclear resonance absorption in hydrated crystals: Fine structure of the proton line. *J. Chem. Phys.* **16**, 327–336 (1948).

56. J. J. Morton, A. M. Tyryshkin, R. M. Brown, S. Shankar, B. W. Lovett, A. Ardavan, T. Schenkel, E. E. Haller, J. W. Ager, S. Lyon, Solid-state quantum memory using the 31P nuclear spin. *Nature* **455**, 1085–1088 (2008).

57. S. Kuzin, G. Jeschke, M. Yulikov, Diffusion equation for the longitudinal spectral diffusion: The case of the RIDME experiment. *Phys. Chem. Chem. Phys.* **24**, 23517–23531 (2022).

58. M. Abobeih, J. Randall, C. Bradley, H. Bartling, M. Bakker, M. Degen, M. Markham, D. Twitchen, T. Taminiau, Atomic-scale imaging of a 27-nuclear-spin cluster using a quantum sensor. *Nature* **576**, 411–415 (2019).

59. G. Van de Stolpe, D. Kwiatkowski, C. Bradley, J. Randall, M. Abobeih, S. Breitweiser, L. Bassett, M. Markham, D. Twitchen, T. Taminiau, Mapping a 50-spin-qubit network through correlated sensing. *Nat. Commun.* **15**, 2006 (2024).

60. M. Flämig, L. Gabrielyan, R. Minikejew, S. Markarian, E. A. Rössler, Dielectric relaxation and proton field-cycling NMR relaxometry study of dimethyl sulfoxide/glycerol mixtures down to glass-forming temperatures. *Phys. Chem. Chem. Phys.* **22**, 9014–9028 (2020).

61. A. Bogdanov, M. Seal, E. Goren, A. Bar-Shir, D. Goldfarb, Host–guest geometry in paramagnetic cavitands elucidated by 19 F electron-nuclear double resonance. *Phys. Chem. Chem. Phys.* **27**, 3885–3896 (2025).

62. E. R. Andrew, R. Bersohn, Nuclear magnetic resonance line shape for a triangular configuration of nuclei. *J. Chem. Phys.* **18**, 159–161 (1950).

63. S. Sternhell, Rotation about Single Bonds in Organic Molecules in *Dynamic Nuclear Magnetic Resonance Spectroscopy* (Academic Press New York, 1975), pp. 163–201.

64. G. Jeschke, L. Esteban-Hofer, "Integrative ensemble modeling of proteins and their complexes with distance distribution restraints" in *Methods in Enzymology* (Elsevier, 2022), vol. 666, pp. 145–169.

65. G. Jeschke, Characterization of conformationally heterogeneous proteins by electron paramagnetic resonance spectroscopy. *Curr. Opin. Struct. Biol.* **92**, 103046 (2025).

66. T. Wu, R. A. Stein, T.-Y. Kao, B. Brown, H. S. Mchaourab, Modeling protein conformational ensembles by guiding AlphaFold2 with double electron electron resonance (DEER) distance distributions. *Nat. Commun.* **16**, 7107 (2025).

67. S. Simmons, R. M. Brown, H. Riemann, N. V. Abrosimov, P. Becker, H.-J. Pohl, M. L. Thewalt, K. M. Itoh, J. J. Morton, Entanglement in a solid-state spin ensemble. *Nature* **470**, 69–72 (2011).

68. K. Herb, T. F. Segawa, L. A. Völker, J. M. Abendroth, E. Janitz, T. Zhu, C. L. Degen, Multidimensional spectroscopy of nuclear spin clusters in diamond. *Phys. Rev. Lett.* **132**, 133002 (2024).

69. S. Stoll, A. Schweiger, EasySpin, a comprehensive software package for spectral simulation and analysis in EPR. *J. Magn. Reson.* **178**, 42–55 (2006).

70. S. Pribitzer, L. Fábregas Ibáñez, C. Gmeiner, I. Ritsch, D. Klose, M. Sajid, M. Hülsmann, A. Godt, G. Jeschke, Two-dimensional distance correlation maps from pulsed triple electron resonance (TRIER) on proteins with three paramagnetic centers. *Appl. Magn. Reson.* **49**, 1253–1279 (2018).

71. O. Sørensen, G. Eich, M. H. Levitt, G. Bodenhausen, R. Ernst, Product operator formalism for the description of NMR pulse experiments. *Prog. Nucl. Magn. Reson. Spectrosc.* **16**, 163–192 (1984).

72. J.-N. Dumez, M. C. Butler, L. Emsley, Numerical simulation of free evolution in solid-state nuclear magnetic resonance using low-order correlations in Liouville space. *J. Chem. Phys.* **133**, 224501 (2010).

73. A. Abragam, *The Principles of Nuclear Magnetism* (Oxford University Press, 1961).

74. M. M. Haugland, A. H. El-Sagheer, R. J. Porter, J. Pena, T. Brown, E. A. Anderson, J. E. Lovett, 2′-Alkynylnucleotides: A sequence-and spin label-flexible strategy for EPR spectroscopy in DNA. *J. Am. Chem. Soc.* **138**, 9069–9072 (2016).

75. G. Úr, T. Kalai, K. Hideg, Facile syntheses of 3, 4-disubstituted pyrroline nitroxides and their further synthetic applications. *Tetrahedron Lett.* **57**, 778–780 (2016).

76. J. Pirrwitz, D. Schwarz, Verfahren zur Herstellung von 2,2,6,6-Tetramethyl-4-oxopiperidin. *DDR patent: DD222017A1 (WP C 07 D/260 901 6), German Democratic Republic* (1984).

77. A. Eggeling, J. Soetbeer, L. Fábregas-Ibáñez, D. Klose, G. Jeschke, Quantifying methyl tunneling induced (de) coherence of nitroxides in glassy ortho-terphenyl at low temperatures. *Phys. Chem. Chem. Phys.* **25**, 11145–11157 (2023).

78. J. Soetbeer, L. F. Ibáñez, Z. Berkson, Y. Polyhach, G. Jeschke, Regularized dynamical decoupling noise spectroscopy – A decoherence descriptor for radicals in glassy matrices. *Phys. Chem. Chem. Phys.* **23**, 21664–21676 (2021).
